# Targeting the NTSR2/TrkB oncogenic pathway in chronic lymphocytic leukemia

**DOI:** 10.1038/s41598-024-56663-5

**Published:** 2024-03-13

**Authors:** Léa Ikhlef, May Yassine, Boutaîna Chandouri, Léa Rivière, Thomas Naves, Natalya Dmytruk, Nathalie Gachard, Marie-Odile Jauberteau, Paul-François Gallet

**Affiliations:** 1 UMR INSERM 1308, CAPTuR, University of Limoges, 2 rue du Docteur Marcland, 87025 Limoges, France; 2 Department of Clinical Hematology, University Hospital of Limoges, Limoges, France; 3 Hematology Laboratory, UMR CNRS7276/INSERM 1262, University Hospital of Limoges, Limoges, France; 4 Immunology Laboratory, University Hospital of Limoges, Limoges, France

**Keywords:** Target identification, Chronic lymphocytic leukaemia

## Abstract

Current therapies that target the B-cell receptor pathway or the inhibition of anti-apoptotic proteins do not prevent the progressive forms of chronic lymphocytic leukemia (CLL), have low long-term efficacy and are subject to therapeutic resistance. Deciphering the mechanisms of leukemic cell survival and searching for new specific targets therefore remain major challenges to improve the management of this disease. It was evidenced that NTSR2 (neurotensin receptor 2), through the recruitment of TRKB (tropomyosin related kinase B), induces survival pathways in leukemic B cells. We have investigated the therapeutic potential of this protein complex as a new target. The binding domain of NTSR2 and TRKB was identified and a peptide targeting the latter was designed. The peptide binds TRKB and efficiently decreases the interaction of the two proteins. It is also effectively internalized by CLL-B cells in which it notably affects Src family kinase signaling and anti-apoptotic proteins levels. It demonstrated a cytotoxic effect both in vitro on the MEC-1 cell line and ex vivo on a cohort of 30 CLL patients. Altogether, these results underline the therapeutic potential of the NTSR2/TRKB protein complex as a target in CLL and open new perspectives for the development of targeted therapies.

## Introduction

Chronic lymphocytic leukemia (CLL) is the most common type of leukemia in adults^1^. It is characterized by a high degree of clinical heterogeneity, influenced by the molecular complexity of the disease. The therapeutic landscape of CLL has undergone profound changes in the past years^2^. The addition of CD20-specific antibodies to conventional chemotherapy has improved the therapeutic outcome of many CLL patients^3^. Since the identification of the critical role of the B cell receptor signaling pathway in the pathogenesis of CLL^4,5^, several molecules have been developed to target it^6^. Ibrutinib and idelalisib, two kinase inhibitors, have both become available for CLL therapy in the first and second line^7,8^. Additionally, the observation of high expression levels of the anti-apoptotic protein BCL-2 in CLL has led to the development of venetoclax, a BH3 mimetic compound that inhibits BCL-2 and has shown high efficacy in some CLL patients^9^. Despite the positive impact of these drugs on patients’ outcomes, their clinical use has introduced new practical challenges for clinicians, mainly due to their adverse events^10^ and the development of drug resistance^11–13^. Many ongoing clinical trials are currently evaluating the efficacy of combinational therapy and various treatment sequencing in order to overcome resistance and improve disease management^14–19^. CLL therefore still represents an incurable disorder that may progress to other more aggressive types of cancer^20,21^. Hence, innovative therapeutic strategies are required to improve patient care. It is essential to identify new druggable molecular pathways to offer patients potentially curative options or at least improved efficacy and reduced toxicity.

In a previous study published by our team^22^, neurotensin receptor 2 (NTSR2), a seven-transmembrane G protein-coupled receptor, was identified as a driver of apoptosis resistance in CLL-B cells. Interestingly, the *NTSR2* gene is located on the short arm of chromosome 2, whose gain has been associated with poor prognosis in CLL^23^. NTSR2 was found to be highly expressed in leukemic B cells^24^, and remained in a constitutively active state, caused by its interaction with the oncogenic tyrosine kinase receptor TRKB, which is also highly expressed in CLL-B cells. Further characterization revealed that the NTSR2–TRKB interaction acts as an oncogenic driver requiring brain-derived neurotrophic factor (BDNF), the natural ligand of TRKB, which is also highly expressed in CLL-B cells^22^. The NTSR2–TRKB interaction activates survival signaling pathways, notably the SRC and AKT kinase pathways, as well as expression of anti-apoptotic proteins BCL-2 and BCL-XL. When NTSR2 was downregulated, TRKB failed to protect CLL-B cells from a significant decrease in viability. Additionally, this interaction was not found in B cells from healthy donors^22^. Together, these findings demonstrated that the NTSR2–TRKB interaction plays a crucial role in CLL-B cell survival, suggesting that its inhibition represents a promising strategy for treating CLL. In the present study, we worked on validating the therapeutic value of targeting this oncogenic interaction. To do so, we designed a peptide whose target is the binding domain between NTSR2 and TRKB, with the goal of inhibiting their interaction, and in turn, their oncogenic signaling.

## Results

### Establishment of the NTSR2/TRKB interaction platform in the HEK293T cell line

In a previous study by Abbaci et al., an oncogenic interaction between NTSR2 and TRKB in CLL-B cells was evidenced^22^. To further investigate this interaction and develop a therapeutic molecule, preliminary experiments were performed in HEK293T cells.

**Figure 1:**
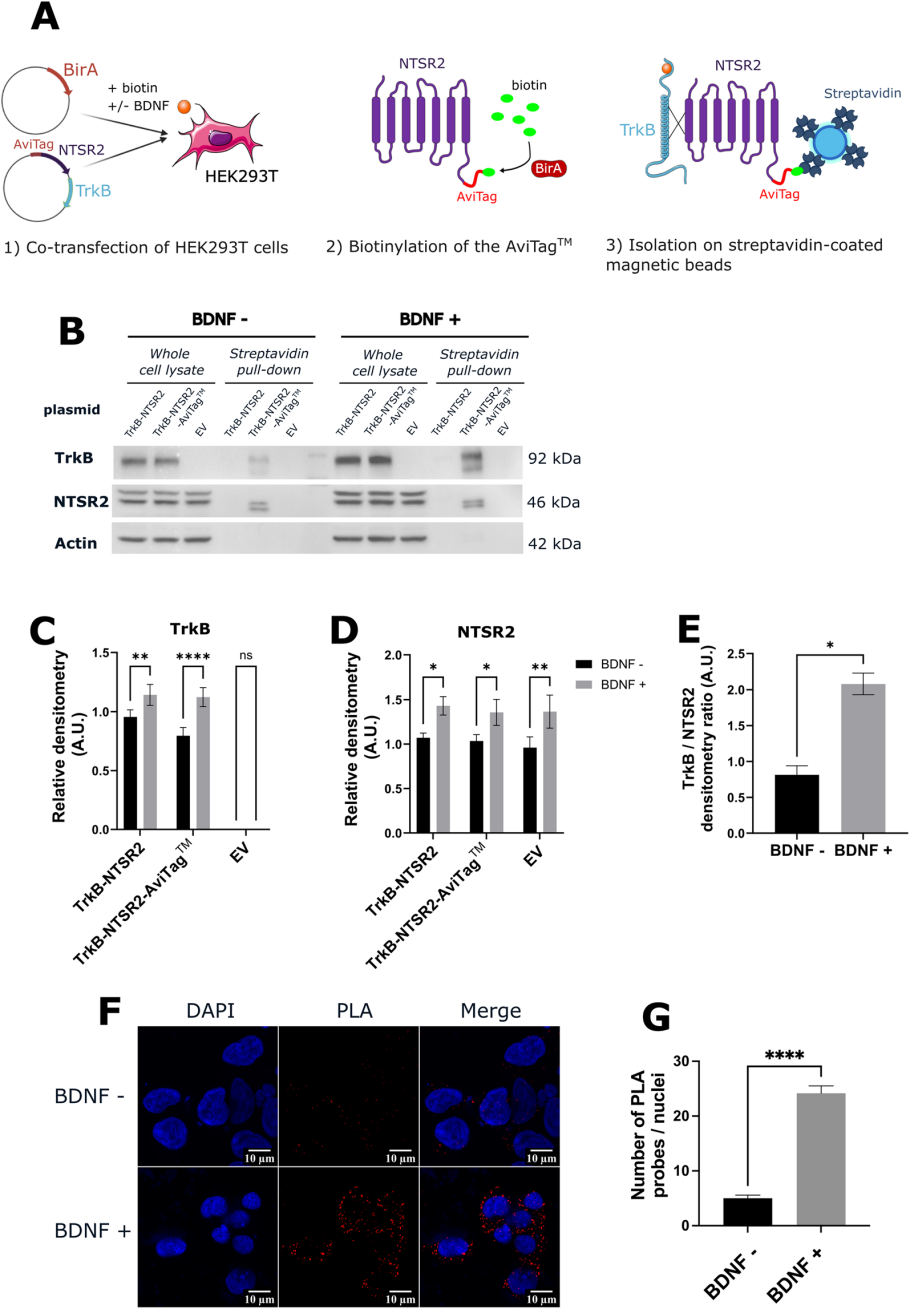
Validation of the NTSR2 and TRKB interaction in the HEK293T cellular model. (**A**) Schematic representation of the experimental approach. HEK293T cells were co-transfected with two plasmids allowing the expression of BIRA, AviTag™–NTSR2, and TRKB proteins. They were treated with 40 µM biotin to enable the biotinylation of the tag and with or without 100 µg/mL of BDNF, the ligand of TRKB. The biotinylated AviTag™–NTSR2 protein was isolated using streptavidin-coated magnetic beads, and the co-isolated proteins were then detected by immunoblotting. (**B**) Representative immunoblot of 3 independent experiments. The interaction between NTSR2 and TRKB proteins was observed through streptavidin pull-down and immunoblotting of the two proteins. No interaction was observed when cells were transfected with the plasmid encoding TRKB and NTSR2 (TRKB–NTSR2), nor with the empty vector (EV). However, the interaction was observed when cells were transfected with the plasmid encoding TRKB and NTSR2-AviTag™ (TRKB–NTSR2–AviTag™). Densitometric analysis of the expression levels of TRKB (**C**) and NTSR2 (**D**) in transfected HEK293T cells revealed an increase in the expression of the two proteins when BDNF is present. (**E**) Densitometric analysis of the TRKB/NTSR2 ratio representing the level of interaction when BDNF is present. (**F**) Proximity ligation assay (PLA) for NTSR2 and TRKB. HEK293T cells were transfected with a plasmid encoding TRKB and NTSR2. The close proximity of the two proteins was visualized as red spots on the cell surface. Nuclei were stained with DAPI (blue). Magnification × 40. (**G**) Quantification of PLA spots per cell was performed in 50 cells, showing an increased interaction between the two proteins when BDNF is present.

HEK293T cells were co-transfected with a plasmid encoding biotin transferase BirA and either a plasmid encoding TRKB and NTSR2 (TRKB–NTSR2), a plasmid encoding TRKB and a version of NTSR2 with an AviTag™ added at the *N*-terminus (TRKB–NTSR2-AviTag™), or an empty vector (EV). Following transfection, biotin was added to the culture medium to enable the biotinylation of the AviTag™ by BirA, and cells were treated or not with BDNF, the ligand of TRKB, which is known to enhance the NTSR2/TRKB interaction^22^. Transfected cells were then lysed and incubated with streptavidin-coated magnetic beads to isolate the biotinylated tagged NTSR2 receptor (Fig. 1A) and identify co-isolated proteins.

After transfection, we confirmed the expression of TRKB and NTSR2 through western blot analysis (Fig. 1B). TRKB was detected in both the TRKB–NTSR2 and TRKB–NTSR2–AviTag™ conditions, but not when an empty vector was transfected. This indicates that the receptor was not expressed in the ‘wild-type’ HEK cells. In contrast, the presence of NTSR2 was confirmed in all three conditions tested. It is worth noting that we observed two distinct bands for NTSR2, similar observations have been made for NTSR1, it was found to be expressed as three distinct protein forms, one being a glycosylated form, another being a N-terminally cleaved and de-glycosylated form, and a last one with the size predicted by its amino acid sequence^25^. Surprisingly, we also observed a slight shift in molecular weight for NTSR2 between the cell lysates and the pull-down samples. The band in pull-down samples appeared to be slightly lower, this could be due to the variation in handling for the two conditions. NTSR2 has various phosphorylation sites^26^, the difference observed could be due to loss of phosphorylation in the pull-down samples^27^. NTSR2 could also possibly slightly vary in structure or conformation due to a modified interactome which could lead to differences in the molecular weight profile compared to whole cell lysate samples.

Densitometric analysis of the protein signals revealed that protein levels were elevated when BDNF was introduced to the culture medium (Fig. 1C,D). While the exact mechanism underlying the BDNF/TRKB signaling pathway requires further investigation, it is possible that an activation of protein synthesis linked to the CREB/mTOR pathway is involved, as previously observed in cortical neurons^28^.

Neither NTSR2 nor TRKB was found in the proteins eluted from the beads when cells were transfected with the untagged version of NTSR2 or the empty vector. However, both proteins were detected in the elution from the pull-down when cells were transfected with the tagged version of NTSR2, indicating that TRKB is co-precipitated (Fig. 1B). Moreover, consistent with previous data^22^, the presence of BDNF enhanced the level of interaction between the two proteins (Fig. 1E).

To further confirm their interaction, a proximity ligation assay analysis was performed (Fig. 1F). This analysis confirmed the proximity of the two proteins. It also demonstrated an increase in the interaction between TRKB and NTSR2 in the presence of BDNF (Fig. 1G).

Overall, these findings provide evidence that the interaction between NTSR2 and TRKB is still effective in HEK293T cells, and that this interaction is enhanced upon BDNF-induced activation.

### Determination of the interaction domain between NTSR2 and TRKB

**Figure 2:**
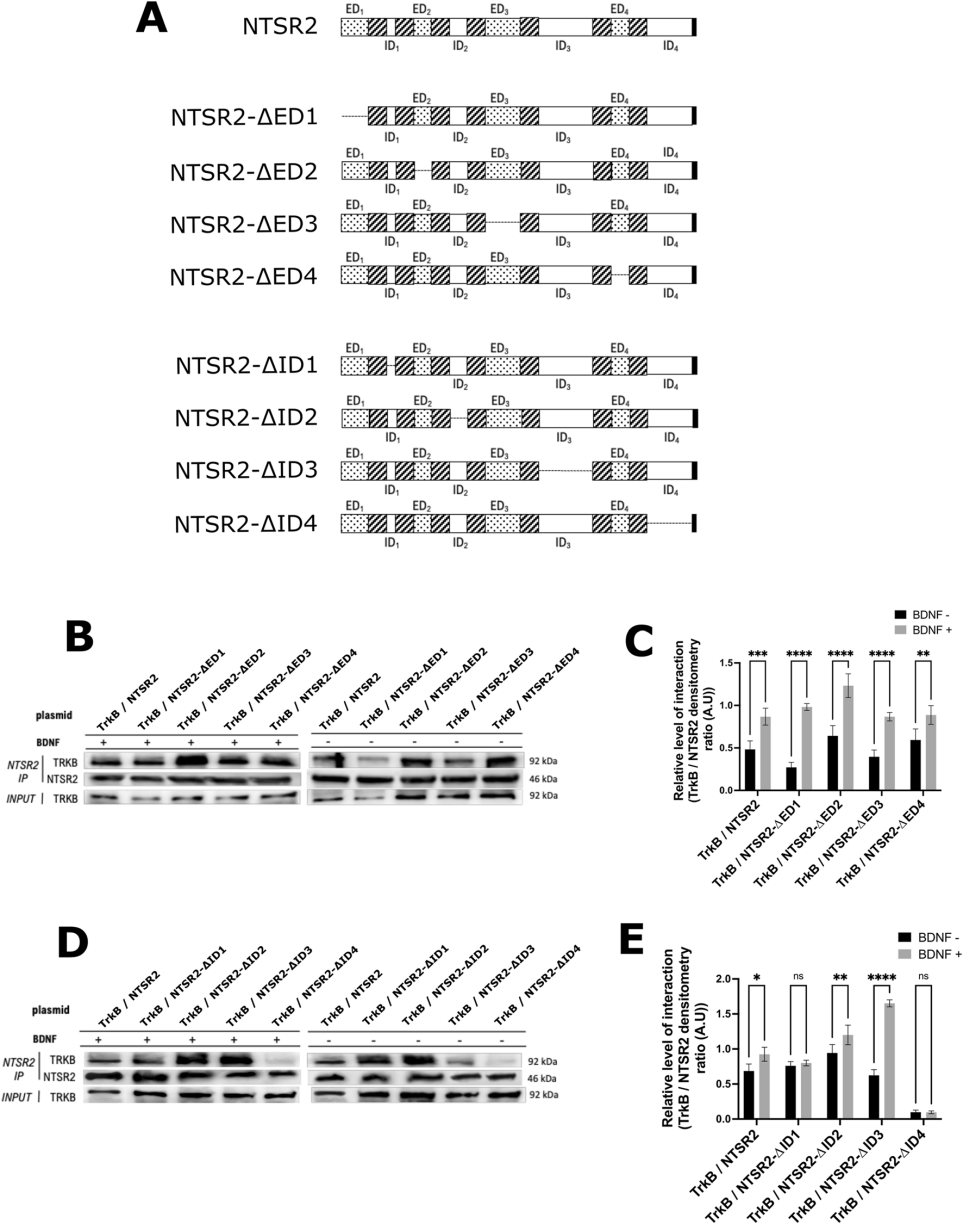
Determination of the interaction domain of NTSR2 with TRKB. (**A**) Illustration of the mutated forms of the NTSR2 protein expressed in HEK293T cells. TRKB is a single transmembrane receptor, while NTSR2 is a seven-transmembrane domain receptor (7TM). Eight truncated forms of NTSR2 were constructed, each with one extracellular (ΔED) or intracellular (ΔID) loop deleted. Loops are represented by portions with dots; transmembrane domains are represented by hatched portions, and the deleted portions are shown by a line. All constructs have the AviTag™ sequence at the C-terminus (shown in black). (**B**) Representative immunoblot of 3 independent experiments. NTSR2 truncated forms with deleted extracellular loops were expressed in HEK293T cells and isolated using streptavidin-coated magnetic beads. For each deleted form, TRKB was detected by immunoblotting, indicating that the deleted loop is not essential for the NTSR2/TRKB interaction. (**C**) Densitometric analysis of the amount of TRKB/NTSR2 interaction with deleted extracellular loops, both with and without BDNF in the culture medium, still showing the importance of BDNF in the TRKB/NTSR2 interaction. (**D**) Representative immunoblot of 3 independent experiments. The NTSR2 truncated forms with deleted intracellular loops were expressed in HEK293T cells and isolated using streptavidin-coated magnetic beads. For each truncated form, except the one with a deleted *C*-terminus part, TRKB was detected by immunoblotting, indicating that the deleted loop is not essential for the NTSR2/TRKB interaction. The low amount of TRKB detected when the *C*-terminus part of NTSR2 is absent signifies that this part of the NTSR2 protein is involved in its interaction with TRKB. (**E**) Densitometric analysis of the amount of TRKB/NTSR2 interaction with deleted intracellular loops, both with and without BDNF in the culture medium, still showing the importance of BDNF in the TRKB/NTSR2 interaction.

As previously observed in CLL-B cells^22^, the NTSR2 and TRKB proteins also interact in HEK293T cells. To determine the specific interacting domains of these two proteins, we designed plasmids encoding truncated versions of NTSR2-AviTag™. Each version had one of the eight extra- and intra-cellular domains removed (Fig. 2A).

The plasmids containing the truncated versions of NTSR2-AviTag™, along with the plasmid encoding for BIRA, were introduced into HEK293T cells. The NTSR2-AviTag™ proteins were then subjected to a pull-down procedure and proteins that were eluted from the magnetic beads were subjected to western blot analysis. Through this analysis, we were able to evaluate the interaction between NTSR2 and TRKB, and the extent of this interaction was quantified using densitometry to calculate the TRKB/NTSR2 ratio (Fig. 2C,E).

The results showed that the interaction of TRKB with the truncated forms of NTSR2 was still present regardless of the extracellular domain removed (ΔED1 to ΔED4; Fig. 2B). As observed previously, the interaction between the two proteins was significantly increased when BDNF was added to the cells (Fig. 2C), regardless of the deleted extracellular domain. Concerning the intracellular domains, the interaction between TRKB and the truncated forms of NTSR2 was always visible for the deletions of intracellular loops 1 to 3 (ΔID1 to ΔID3). Strikingly, a very weak or even absent co-immunoprecipitation of TRKB was observed when the C-terminal end of NTSR2 was deleted (NTSR2–ΔID4) (Fig. 2D), indicating that this part of the NTSR2 protein is involved in the interaction with TRKB.

Overall, these findings provide evidence that the interaction between NTSR2 and TRKB is primarily associated with the C-terminal end of NTSR2. Furthermore, they confirm that BDNF activation enhances this interaction, reinforcing the importance of BDNF signaling in regulating NTSR2/TRKB-related pathways.

### Targeting the NTSR2/TRKB interaction induces leukemic B cell death

The previous data suggests that the interaction between NTSR2 and TRKB occurs through the *C*-terminus region of NTSR2. To inhibit this interaction, a peptide named *Pep* was generated, which corresponds to the *C*-terminal sequence of the NTSR2 protein. This peptide was used as a competitor for the NTSR2/TRKB interaction. In addition to the *Pep* peptide, we also produced variants: we added a ‘TAT’ cell-penetrating peptide sequence to enhance cell penetration^29^, and/or a V5-tag for subsequent fluorescent labelling (Table 1).

**Table 1:** Amino acid sequences of the synthesized peptides.

Name	Amino acid sequence	Sequence characteristics
TAT	GRKKRRQRRRPPQ	Cell penetrating peptide
*Pep*	**NAVSSSFRKLFLEAVSSLCGEHHPMKRLPPKPQSPTLMDTASGFGDPPETRT**	Therapeutic peptide
*Pep*-V5	**NAVSSSFRKLFLEAVSSLCGEHHPMKRLPPKPQSPTLMDTASGFGDPPETRT**GKPIPNPLLGLDST	V5 tagged peptide
TAT-*Pep*	GRKKRRQRRRPPQ**NAVSSSFRKLFLEAVSSLCGEHHPMKRLPPKPQSPTLMDTASGFGDPPETRT**	CPP tagged peptide
TAT-*Pep*-V5	GRKKRRQRRRPPQ**NAVSSSFRKLFLEAVSSLCGEHHPMKRLPPKPQSPTLMDTASGFGDPPETRT**GKPIPNPLLGLDST	CPP and V5 tagged peptide

Peptides were produced by GeneCust (Boynes France).

The sequence of intracellular domain 4 is in bold.

**Figure 3:**
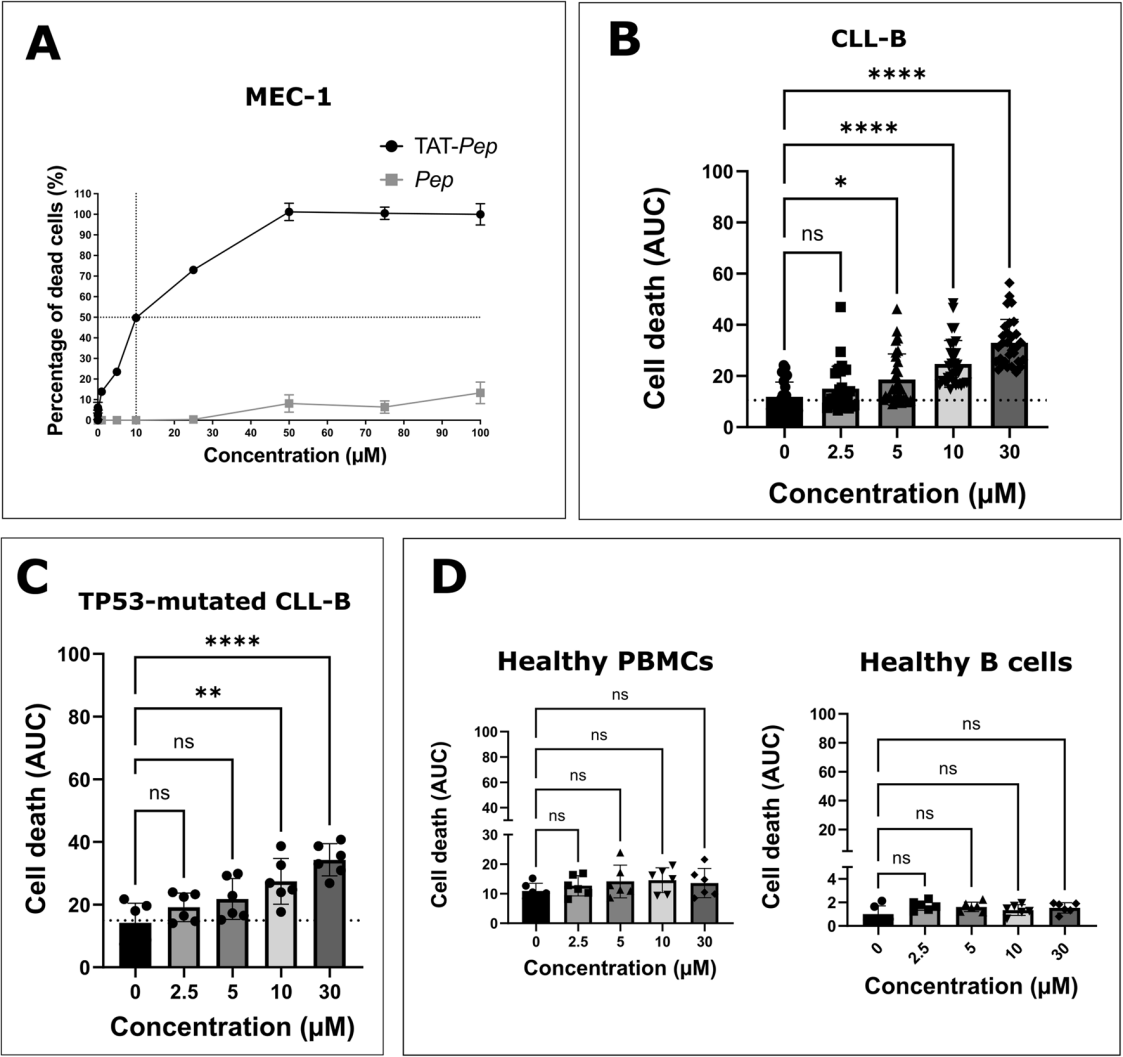
Cytotoxicity of the peptide in vitro and ex vivo. (**A**) Determination of the cytotoxicity of *Pep* and TAT-*Pep* peptides on the MEC-1 cell line. Increased concentrations (0 to 100 µM) of *Pep* and TAT-*Pep* were incubated with the MEC-1 cell line, and the number of dead cells was measured using the Incucyte live-cell imaging device with Cytotox Dye (green). Fluorescence was monitored for 24 h. (**B**) Measurement of the cytotoxicity of the TAT-*Pep* peptide on CLL-B cells from patients. Increased concentrations (0 to 30 µM) of TAT-*Pep* were incubated with CLL-B cells, and the number of dead cells was measured using the Incucyte live-cell imaging device with Cytotox Dye (green). Fluorescence was monitored for 48 h. Experiments were conducted on 30 different patients, each in technical triplicates. (**C**) Evaluation of TAT-*Pep*’s cytotoxicity on CLL-B cells from 6 patients bearing TP53 mutations (Patients ID numbers: 2, 6, 7, 9, 11 and 12). The same protocol as in (**B**) was applied. (**D**) Cytotoxicity of the TAT-*Pep* peptide on PBMCs from healthy donors as well as isolated healthy B cells. The same protocol as in (**B**) was applied. Increased concentrations (0 to 30 µM) of TAT-Pep were incubated with PBMCs or B cells from 6 different individuals, each in technical triplicates. In all analyses, the ‘Green Object Count/Phase Object Count’ ratio was determined using the Incucyte analysis software to obtain the percentage of dead cells. The area under the curve for each condition was then calculated, and they were compared using a t-test.

The cytotoxicity of the peptides was initially assessed on the MEC-1 cell line to determine their IC50. The *Pep* peptide alone did not exhibit significant cytotoxicity; however, the TAT-*Pep* peptide induced cell death with an IC50 of 10 µM (Fig. 3A).

Next, we conducted tests using the TAT-*Pep* peptide on CLL-B cells from a cohort of thirty patients. The level of expression of both NTSR2 and TrkB in all 30 patients was evaluated by flow cytometry, as expected, both targets were found to be highly expressed in CLL-B cells compared to heathy B cells, as shown on Supplementary Fig. S1A. Characteristics of the patients included in the study are available in Supplementary Table 1. We treated 20,000 CLL-B cells with a range of peptide concentrations (0, 2.5, 5, 10 and 30 µM). To make sure that the observed cell death was specifically attributed to the NTSR2 sequence and not the TAT CPP sequence, we tested the cytotoxicity of the TAT peptide on CLL-B cells from ten patients at the same concentrations. TAT-induced cytotoxicity data is available in Supplementary Fig. S2, as expected, the TAT peptide did not induce any significant cytotoxicity. At the concentration of 2.5 µM of TAT-*Pep*, no significant cytotoxicity was observed. However, at 5 µM, there was a 1.6-fold increase in cell death compared to the control. It progressively rose to a two-fold increase at 10 µM and a 2.8-fold increase at 30 µM (Fig. 3B). Among our cohort, 6 of the patients bore *TP53* mutations (patients 2, 6, 7, 9, 11 and 12). As shown in Fig. 3C, despite their unfavorable mutation statuses, they still responded to TAT-*Pep* treatment. We observed an increase in cell death following treatment, however, it is significant only starting at 10 µM of TAT-*Pep*, with a 1.9-fold increase in cell death compared to the control. As for the 30 µM treatment condition, we observed a 2.4-fold increase compared to the control.

We also examined the same peptide concentrations on peripheral blood mononuclear cells (PBMC) from healthy donors as well as isolated B cells from six healthy donors (Fig. 3D). Remarkably, the peptide did not significantly affect cell viability, indicating its specificity towards leukemic B cells. This finding highlights the promising therapeutic potential, as TAT-*Pep* selectively targets cancerous cells without causing substantial harm to healthy cells.

To further underline the pathological nature of the NTSR2/TRKB interaction and confirm previously published results^22^, we checked for the expression of both proteins in PBMCs isolated from ten healthy individuals using flow cytometry. Results showed that TRKB was expressed in both healthy B and T cells but that, conversely, NTSR2 was not expressed in either population (Supplementary Fig. S1B).

### The peptide is efficiently internalized in patients B cells and decreases the interaction between NTSR2 and TRKB

**Figure 4:**
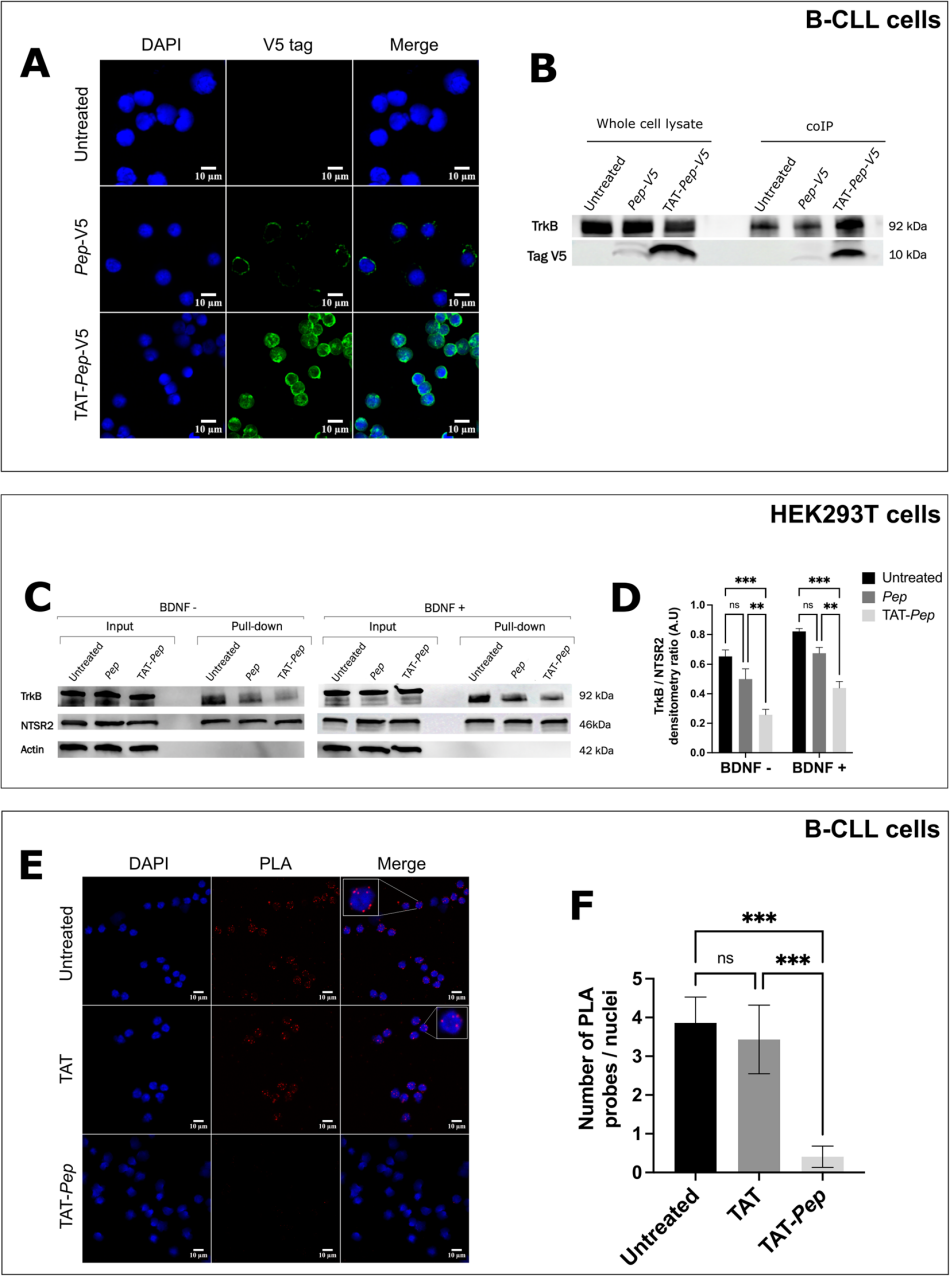
The peptide is internalized in patients B cells and effectively decreases the interaction between NTSR2 and TRKB. (**A**) Immunofluorescence analysis of *Pep*-V5 and TAT-*Pep*-V5 peptide internalization in B cells of patients with chronic lymphocytic leukemia. CLL-B cells from 3 patients were incubated with 10 µM of peptide overnight, and the V5 Tag was detected using an anti-V5 primary antibody (#R96025, ThermoFisher) and an Alexa Fluor™ 488 anti-mouse IgG secondary antibody (#A-11001, ThermoFisher, green fluorescence). Nuclei were stained with DAPI (blue). Only the TAT-*Pep* peptide was able to penetrate the CLL-B cells. (**B**) Immunoblotting analysis of the interaction between TRKB and *Pep*. Patients B cells were treated overnight with 10 µM of *Pep*-V5 or TAT-*Pep*-V5 peptide. TRKB was then immunoprecipitated using protein A-coated magnetic beads pre-incubated with an anti-TRKB antibody, and *Pep* was detected with an anti-V5 antibody. The TAT-*Pep*-V5 peptide efficiently co-immunoprecipitated with the TRKB protein. (**C**) Representative immunoblot of 3 independent experiments. Co-immunoprecipitation analysis of the inhibition of NTSR2/TRKB interaction by TAT-*Pep*. HEK293T cells transfected with a plasmid encoding TRKB and AviTag™-NTSR2 were incubated overnight with 10 µM of *Pep* or TAT-*Pep* peptides. The tagged NTSR2 was isolated on streptavidin-coated magnetic beads, and TRKB was detected by immunoblotting. The TRKB/NTSR2 interaction was significantly decreased when TAT-*Pep* was added to the culture medium. (**D**) Densitometric analysis of the TRKB/NTSR2 ratio in the presence of *Pep* or TAT-*Pep*, with and without BDNF in the culture medium. (**E**) Proximity ligation assay (PLA) analysis of NTSR2/TRKB interaction in the presence of TAT-*Pep*. Patients B cells were incubated overnight with 10 µM TAT or TAT-*Pep* peptides. The close proximity of the two proteins was visualized as red spots. Nuclei were stained with DAPI (blue). Magnification × 40. (**F**) Quantification of PLA spots per cell was performed in 50 cells, showing a decrease in the interaction between the two proteins when TAT-*Pep* is present.

The TAT-*Pep* peptide that we developed is designed to inhibit the interaction between NTSR2 and TRKB proteins. We proceeded to characterize the properties of this potential therapeutic peptide candidate. To assess its ability to penetrate patients' B cells, we treated the B cells with 10 µM of either the TAT-*Pep*-V5 or *Pep*-V5 peptide. Subsequently, the peptides were detected using an anti-V5 tag antibody (appearing in green), while the cell nuclei were stained with DAPI (appearing in blue) (Fig. 4A).

As anticipated, we observed a weak green fluorescence in CLL-B cells treated with the *Pep*-V5 peptide, suggesting that the *Pep*-V5 peptide has limited or minimal penetration into the cells.

Conversely, for the cells incubated with the TAT-*Pep*-V5 peptide, we observed an intense green fluorescence, indicating that the TAT-*Pep*-V5 peptide effectively penetrates the cells.

Next, we proceeded to evaluate the ability of the peptide to inhibit the NTSR2/TRKB interaction by competing with the NTSR2 protein for binding to its target, TRKB. This assessment aimed to determine whether the TAT-*Pep*-V5 peptide could effectively disrupt the interaction between NTSR2 and TRKB. Patients’ CLL-B cells were treated with either 10 µM of the *Pep*-V5 or the TAT-*Pep*-V5 peptides. Subsequently, TRKB was immunoprecipitated from the cell lysate, and *Pep*-V5 and TAT-*Pep*-V5 peptides were detected using an anti-V5 antibody (Fig. 4B). As anticipated, the TAT-*Pep*-V5 peptide was co-immunoprecipitated with the TRKB protein, as clearly observed on the immunoblot. This indicates that the TAT-*Pep*-V5 peptide effectively enters the CLL-B cells and binds to the intracellular domain of the TRKB protein. The successful binding of the TAT-*Pep*-V5 peptide to TRKB demonstrates its potential as an effective inhibitor of the NTSR2/TRKB interaction, which could have significant implications for disrupting leukemic B cell growth and survival pathways.

To confirm that the peptide binding to TRKB actively inhibits the NTSR2/TRKB interaction, we first performed an NTSR2-AviTag™ pull-down as previously described on transfected HEK293T cells incubated overnight with 10 µM of both the *Pep* and TAT-*Pep* peptides. The amount of co-immunoprecipitated TRKB protein was detected by immunoblot (Fig. 4C,D). As expected, the incubation of HEK293T cells with the TAT-*Pep* peptide resulted in a significant decrease in the amount of co-immunoprecipitated TRKB protein. This demonstrates that the TAT-*Pep* peptide effectively disrupts the NTSR2/TRKB interaction. When HEK293T cells were incubated with the *Pep* peptide, the amount of co-immunoprecipitated TRKB protein was also decreased, although not significantly, as indicated by the densitometric analysis (Fig. 4D). This is associated to the low penetration of the peptide in the cells.

Notably, the addition of BDNF in the culture medium did not restore the interaction between the two proteins, as shown in Fig. 4C and further supported by the densitometric analysis (Fig. 4D). This reinforces the fact that the inhibitory action of the TAT-*Pep* peptide is specific and not dependent on the presence of BDNF.

In a second approach, we investigated the inhibition of the NTSR2/TRKB interaction in CLL-B cells from three CLL patients using PLA on NTSR2 and TRKB. The leukemic B cells were either treated with the TAT-*Pep* peptide, TAT alone, or left untreated, and then the proximity ligation assay was performed (Fig. 4E,F). As anticipated, the red fluorescence spots representing the proximity of NTSR2 and TRKB proteins were dramatically reduced in CLL-B cells incubated with the TAT-*Pep* peptide, in comparison to cells treated with TAT alone or left untreated. This result provides further evidence that the TAT-*Pep* peptide effectively inhibits the interaction between NTSR2 and TRKB proteins in CLL-B cells, potentially hindering signaling pathways crucial for the survival and growth of leukemic B cells.

### The therapeutic peptide affects SRC family kinase activation and inhibits the expression of anti-apoptotic proteins

**Figure 5:**
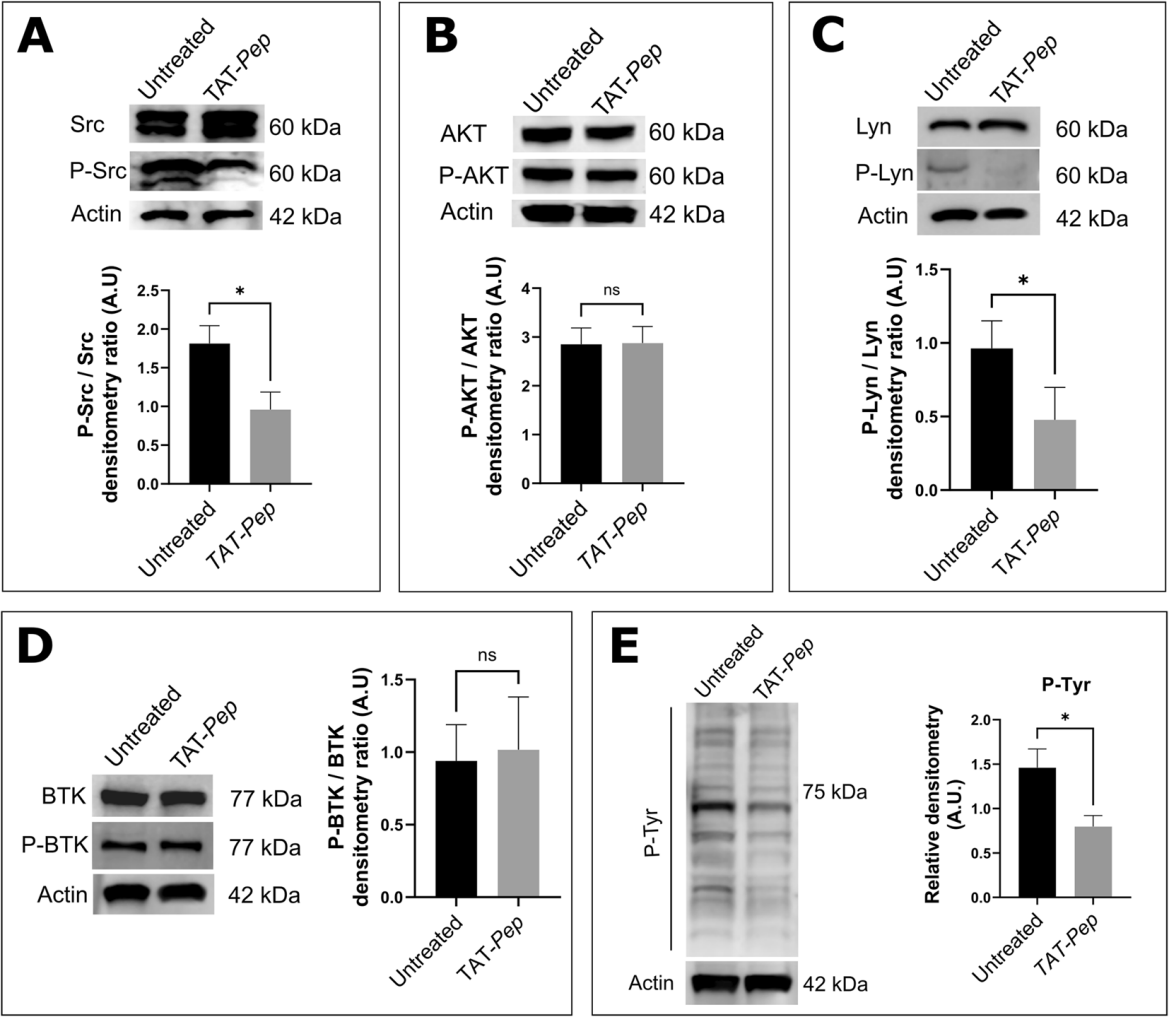
The peptide inhibits kinase phosphorylation. CLL-B cells from 10 patients were incubated overnight, with or without 10 µM of TAT-Pep. Whole cell lysates were then prepared, and 10 µg of proteins were immunoblotted to assess the following parameters. (**A**) Relative phosphorylation level of SRC. (**B**) Relative phosphorylation level of AKT. (**C**) Relative phosphorylation level of LYN. (**D**) Relative phosphorylation level of BTK. (**E**) Relative phosphorylation level of all tyrosine-phosphorylated proteins.

In their paper, Abbaci et al. have shown that the NTSR2/TRKB interaction activates the SRC protein and induces an increased amount of anti-apoptotic proteins BCL-2 and BCL-XL^22^. We then analyzed the impact of the TAT-*Pep* peptide on the activation of the aforementioned proteins in CLL-B cells (Fig. 5).

After treatment, a significant decrease in the amount of phosphorylated SRC was observed, confirming the implication of the protein in the survival pathway (Fig. 5A). However, we did not observe any variation in AKT phosphorylation, suggesting that the PI3K/AKT pathway is not affected by the peptide (Fig. 5B). We also evaluated the level of LYN phosphorylation (Fig. 5C), as it is another SRC family member whose involvement in CLL is well described^30^, results also showed a significant decrease in phosphorylation following treatment. Additionally, we checked for the effect of TAT-*Pep* on the activation on Bruton Tyrosine Kinase (BTK) (Fig. 5D), we found that the peptide did not affect BTK phosphorylation, suggesting that it does not directly affect the BCR pathway. Finally, we evaluated the global level of tyrosine phosphorylation and found evidence that the treatment induces a general decrease in tyrosine phosphorylation (Fig. 5E), suggesting that several signaling pathways are either non-activated or less activated in CLL-B cells treated with the TAT-*Pep* peptide.

**Figure 6:**
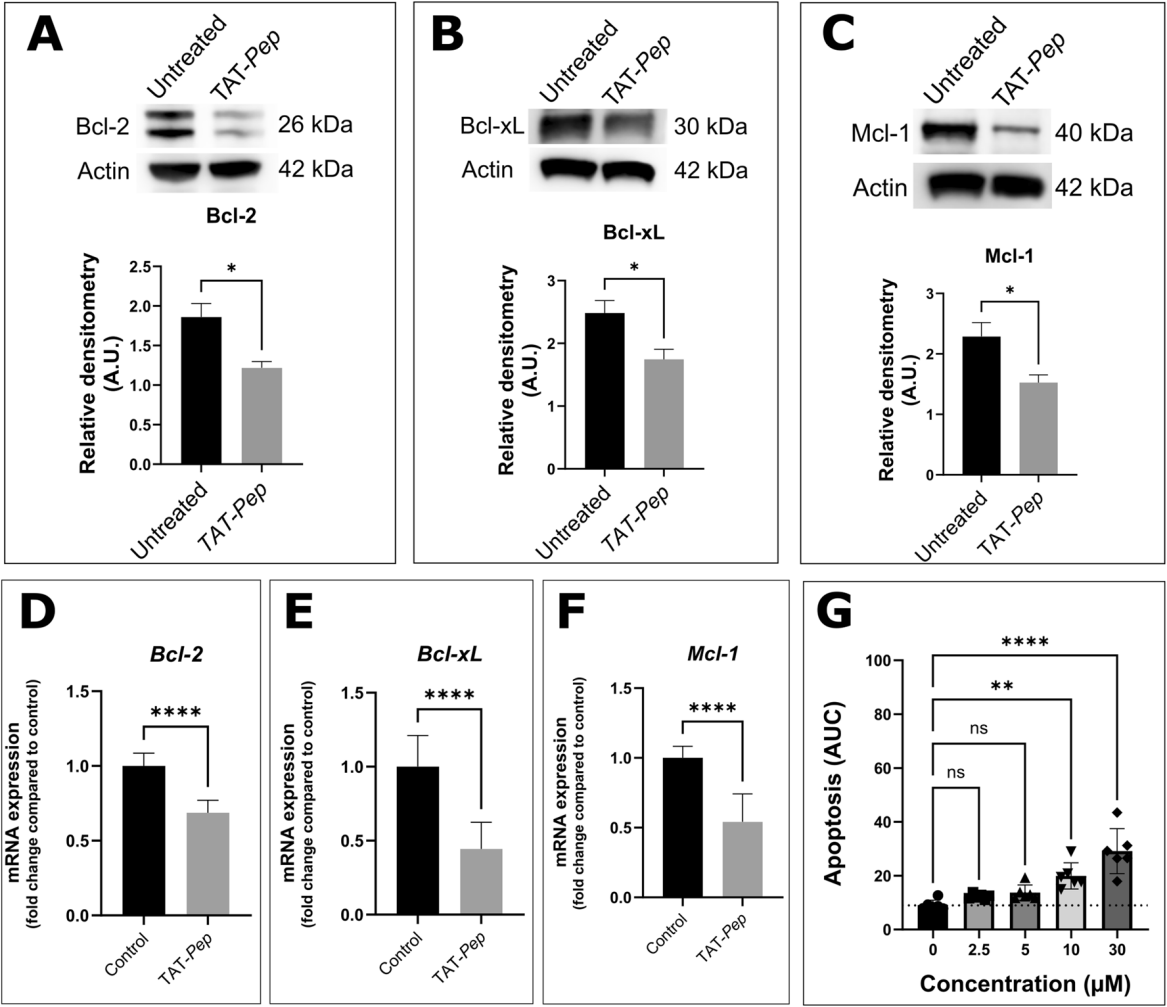
The peptide lowers anti-apoptotic proteins levels. CLL-B cells from 10 patients were incubated overnight, with or without 10 µM of TAT-*Pep*. Whole cell lysates were then prepared, and 10 µg of proteins were immunoblotted to assess the following parameters. (**A**) Relative level of the anti-apoptotic protein BCL-2 in B-CLL. (**B**) Relative level of the anti-apoptotic protein BCL-XL and (**C**) relative level of the anti-apoptotic protein MCL-1. CLL-B samples from ten different patients were utilized for the analysis. CLL-B cells from 12 patients were incubated overnight, with or without 10 µM of TAT-*Pep*, subsequently RNA was extracted from cell lysates and mRNA levels of *BCL-2* (**D**); *BCL-XL* (**E**) and *MCL-1* (**F**) were evaluated using RTqPCR; *18S* was used as a housekeeping gene. (**G**) Increased concentrations (0 to 30 µM) of TAT-*Pep* were incubated with 20,000 CLL-B, and the number of Annexin V-positive cells was measured using the Incucyte live-cell imaging device. Fluorescence was monitored for 48 h. Experiments were conducted on 6 different patients, each in technical triplicates. The ‘Green Object Count/Phase Object Count’ ratio was determined using the Incucyte analysis software to obtain the percentage of apoptotic cells. The area under the curve for each condition was then calculated, and they were compared using a t-test.

Concerning anti-apoptotic protein expression, a decrease in anti-apoptotic proteins BCL-2; BCL-XL and MCL-1 was observed after TAT-*Pep* treatment of CLL-B cells, possibly as a consequence of the SRC kinase inactivation (Fig. 6A–C). To unveil whether the regulation mechanism behind the observed modifications was transcriptional or post-transcriptional, we evaluated the mRNA levels of all three anti-apoptotic proteins following peptide treatment. Results showed a significantly lowered expression for all three following treatment (Fig. 6D–F), suggesting that the regulation might occur transcriptionally. To further underline that the induction of cell death following TAT-*Pep* treatment involved apoptosis regulation pathways, we used B cells from six CLL patients and stained them with Annexin V in both TAT-*Pep* treated and untreated conditions. As shown on Fig. 6G, TAT-*Pep* treatment did induce apoptosis in CLL-B cells. At the concentration of 2.5 µM, no significant cytotoxicity was observed. However, at 5 µM, there was already a 1.9-fold increase in cell death compared to the control. It progressively rose to a 2.5-fold increase and a 3.6-fold increase for 10 µM and 30 µM, respectively.

## Discussion

Chronic lymphocytic leukemia remains an area with a significantly unmet medical need, despite advances in therapy. New therapeutic options are extremely limited for patients with del 17p and those who may be refractory to initial treatment with immunochemotherapy^31^. Although targeted therapies have improved the therapeutic management in CLL, it remains unclear how durable their positive impact on patient outcome will be and the relapse of patients under these novel agents poses a new challenge. Thus, there remains a need for the development of new therapies to achieve better and more durable responses in patients. Our study explores the targeting of an unexplored pathway in CLL, with the aim of demonstrating its therapeutic potential.

Previous work within our team evidenced the interaction of two surface receptors, NTSR2 and TRKB, and their involvement in the activation of survival pathways in CLL-B cells, notably through the activation of kinases SRC and AKT and the overexpression of anti-apoptotic proteins BCL-2 and BCL-XL^22^. In the present study, we determined the binding domain of these two proteins and formulated a peptide targeting it, to evaluate the therapeutic interest of this pathway in the treatment of CLL. The designed peptide proved to be cytotoxic to CLL-B cells but not to healthy donors’ PBMCs, indicating its specificity for cancer cells which could suggest a potentially low toxicity. We also evidenced that following peptide treatment, a decrease in SRC and LYN activation as well as a decrease in the amount of anti-apoptotic proteins BCL-2, BCL-XL and MCL-1 and their transcripts was observable in CLL-B cells, thus confirming some of the molecular actors described in previous published work^22^ and evidencing new actors at play. SRC has been shown to be involved in various processes in CLL such as proliferation, angiogenesis, migration, differentiation, invasion, and immune function^32^. The activation of SRC kinase may result from interactions with cell membrane receptors, as it is the case in our study, or post-translational modifications^33^. As for LYN kinase, it has been shown to be essential for CLL progression, as demonstrated by significantly reduced CLL burden in vivo in the absence of LYN^34^. Loss of LYN within leukemic cells notably reduced BCR signaling, which is crucial for CLL development and progression. We have also checked for the effect of TAT-*Pep* on another kinase which well described for its role in BCR signaling: BTK. It is essential for the survival of CLL cells, and its constitutive activity in CLL has made it a target for inhibition by BTK inhibitors such as ibrutinib, which has had great clinical success in CLL^35^. TAT-*Pep* had no effect on BTK activation, suggesting that it might not target the BCR pathway, as most clinically used novel agents do in CLL.

Peptide-based agents provide a potential solution to overcome drug resistance by disrupting protein–protein interactions without directly binding to the target protein, and instead interacting with binding partners of the target protein, thereby potentially overcoming resistance induced by mutations in target genes or alternative signaling pathways^36^. Therapeutic peptides are gaining attractiveness and show great promise in improving CLL patients care, for instance, preclinical data evaluating the effect of *N*-methylated thrombospondin-1-derived peptides show its potential in specifically killing CLL-B cells^37^. Work published by Simon-Gracia et al. also demonstrates that new tumoral-addressed chimeric peptides for the selective inhibition of PP2A/SET interaction have potential for the treatment of CLL^38^. Our work follows a similar path, highlighting the therapeutic potential of a peptide targeting the NTSR2/TRKB oncogenic signaling platform in the management of chronic lymphocytic leukemia.

## Methods

### Ethical approval and consent to participate

The study was approved by the Limoges University Hospital Institutional Review Board, AC 72-2011-18. All 30 patients (Supplementary Table 1) and 10 healthy donors provided informed consent to participate, all experiments were performed in accordance with relevant named guidelines and regulation. Patients’ prognostic criteria were determined by mutational and cytogenetic analyses according to Rossi et al.^39^ and Nguyen-Khac et al.^40^.

### Design of the peptide

The peptides were produced by GeneCust (Boynes, France). Peptides were designed using the identified target sequence, sequences are detailed in Table 1. Various peptides were produced: one being the native sequence of the C-terminal domain of NTSR2; another with an added TAT cell penetrating peptide (CPP) to enable more efficient cell penetration; another with an added V5 tag to enable tracking of the peptide location by immunofluorescence. Finally, we produced the TAT sequence alone as a control to check for its eventual cytotoxicity.

### Primary culture

CLL-B cells were isolated from patients’ blood using the MACSxpress® Whole Blood B-CLL Cell Isolation Kit (Miltenyi). After isolation, the viability and quantity of isolated CLL-B cells were assessed using a cell counter (LUNA II). CLL-B cells were then frozen in heat-inactivated fetal bovine serum (FBS) supplemented with 10% DMSO. For culturing and analyses, the frozen CLL-B cells were thawed and cultured at 37 °C in 5% CO_2_ in RPMI 1640 medium (Gibco) supplemented with 10% FBS, 100 Units/mL penicillin, 100 µg/mL streptomycin and 0.1% β-mercaptoethanol.

### Cell line culture

The HEK293T cell line was cultured in DMEM GlutaMAX medium (Gibco) supplemented with 10% FBS, 100 Units/mL penicillin, 100 µg/mL streptomycin and 1% non-essential amino acids. The MEC-1 cell line was cultured in RPMI 1640 medium supplemented with 10% FBS, 100 Units/mL penicillin, 100 µg/mL streptomycin and 0.1% β-mercaptoethanol. Cells were cultured at 37 °C in 5% CO_2_.

### Transfection

Vectors used were constructed by Vector Builder. The vector builder IDs are VB201022-1029qpx (BirA); VB201022-1027yza (TRKB-NTSR2) and VB201022-1012yjb (TRKB-NTSR2-AviTag™). 500 000 HEK293T cells were seeded and transfected the following day with 3 µg of plasmid using jetPEI (Polyplus Transfection). They were then treated with 40 µM biotin and with or without 100 µg/mL of BDNF (ABclonal).

### Flow cytometry

Cells were counted and their viability was assessed using trypan blue staining. They were then seeded in a 96 V-well plate at a density of 100,000 cells/well. They were first incubated with BD Pharmingen™ Human BD Fc Block™ (#564219, BD Biosciences) for 10 min at room temperature. They were then washed using PBS 2% BSA and incubated with primary antibodies for 60 min at 4 °C. They were then washed once again and incubated with secondary antibodies and/or primary fluorochrome-coupled antibodies for 60 min at 4 °C. They were washed again before being fixed using 2% paraformaldehyde (ThermoFisher). They underwent a final wash and were resuspended in 200 μL of PBS 2% BSA. Fluorescence was measured using the CytoFLEX LX device (Beckman Coulter) and data analyzed using the Kaluza software (Beckman Coulter). Antibodies used are listed in Supplementary Table 3. Raw flow cytometry data is available at the end of the Supplementary Data Appendix.

### Streptavidin-coated magnetic beads pull-down

Proteins were extracted using RIPA buffer (Sigma-Aldrich) and sonication (VibraCell™). They were then incubated with 30 μL of streptavidin-functionalized magnetic beads (Invitrogen) for 2 h at 4 °C under rotation. For western blot analysis, the magnetic beads were washed 4 times with cold PBS and the fixed proteins were eluted in 4 × Laemmli buffer supplemented with 10% β-mercaptoethanol and denatured at 95 °C for 5 min.

### Western blot analysis

Whole cell lysate proteins were extracted using RIPA buffer (Sigma-Aldrich) and sonication (VibraCell™). Plasma membrane proteins were recovered by using the Plasma Membrane Protein Extraction Kit (Abcam) according to the manufacturer’s instructions. A list of the antibodies used for immunoblotting is available in Supplementary Table 2. Bands were quantitatively assessed by densitometry using Fiji software. To evaluate the level of interaction of NTSR2 and TRKB we normalized the density of the TRKB bands by the density of the NTSR2 bands to obtain the relative amount of TRKB co-precipitated through NTSR2.

### Immunoprecipitation

100 µL of protein A-functionalized magnetic beads (Invitrogen) were incubated for 10 min at room temperature with 1 μg of antibody. After washing, the beads are resuspended in 100 μg of CLL-B cell lysate and incubated overnight at 4 °C. Beads are then washed and the immunoprecipitated proteins are eluted and subjected to western blot analysis.

### Immunofluorescence

Patients B cells were seeded in a poly-l-ornithine-coated (Sigma-Aldrich) 8-well MilliCell EZ slide (Merck Millipore) and treated overnight with the tagged peptide. Following treatment, they were washed thrice using 1× DPBS (Sigma-Aldrich) and fixed with methanol at − 20 °C for 10 min. They were once again washed three times with 1× DPBS, they were then blocked with 5% BSA for 1 h at room temperature. Fixed and blocked cells were incubated overnight at 4 °C with the anti-V5 antibody (#R96025, ThermoFisher) at a dilution of 1/200 and then incubated for 1 h at room temperature with a goat anti-mouse secondary antibody Alexa Fluor™ 488 (#A-11001, ThermoFisher) at a dilution of 1/1000. They were washed three times one last time and images were captured at × 60 magnification using the Zeiss LSM880 confocal microscope.

### Proximity ligation assay (PLA)

Cells were fixed with methanol at − 20 °C for 10 min and then the Duolink kit (Sigma) was used to perform the PLA according to the manufacturer’s instructions. Before observation, cells were mounted on glass slides with DAPI-containing mounting medium and results were observed by confocal microscopy (LSM880, Zeiss) and analyzed using the Fiji software (ImageJ).

### Evaluation of cell proliferation, cell death and apoptosis

Cell viability and survival were monitored using the IncucyteS3 system (Sartorius), a live-cell imaging device enabling the tracking of fluorescence over set periods of time^41,42^. Apoptotic cells were labeled with the Incucyte® Annexin V Dye (Sartorius), while dead cells were marked with the Incucyte® Cytotox Dye for Counting Dead Cells (Sartorius). In all analyses, the ‘Green Object Count/Phase Object Count’ ratio was determined using the Incucyte analysis software to obtain the percentage of dead cells. The area under the curve for each condition was then calculated, and they were compared using a t-test. To evaluate proliferation, phase confluence was determined and normalized with t = 0 h using the Incucyte analysis software.

### RNA extraction and RTqPCR analysis

Total RNA were extracted using the Quick-RNA™ Microprep Kit (Zymo Research) and quantified by NanoDrop 2000 (Thermo Fischer). For RT-qPCR analyses, 2 µg of RNA were reverse-transcribed into cDNA using the cDNA Archive kit (Applied Biosystems). Quantitative gene expression was performed using SensiFAST Probe Hi-ROX kit (Bioline, London) on a QuantStudio 5 (Thermo Fischer). Results were normalized to 18S expression and analyzed using the ∆∆Ct method.

### Statistical analyses

Statistical analyses were performed using GraphPad Prism 9.0. Cytotoxicity experiments were compared using a one-way ANOVA followed by a multiple comparisons test of the different conditions to the control. Western blots densitometry data were compared using either a t-test or a two-way ANOVA. P values ****P < 0.0001, ***P < 0.001, **P < 0.01, *P < 0.05 were considered significant.

### Supplementary Information

Supplementary Information 1.

Supplementary Information 2.

Supplementary Information 3.

## Data Availability

Data will be made available upon reasonable request to the corresponding author.

## References

[1] Hallek M, Al-Sawaf O (2021). Chronic lymphocytic leukemia: 2022 update on diagnostic and therapeutic procedures. Am. J. Hematol..

[2] Delgado J, Nadeu F, Colomer D, Campo E (2020). Chronic lymphocytic leukemia: From molecular pathogenesis to novel therapeutic strategies. Haematologica.

[3] Al-Sawaf O, Fischer K, Eichhorst B, Hallek M (2016). Targeted therapy of CLL. Oncol. Res. Treat..

[4] Burger JA, Chiorazzi N (2013). B cell receptor signaling in chronic lymphocytic leukemia. Trends Immunol..

[5] Almasri M (2022). Druggable molecular pathways in chronic lymphocytic leukemia. Life.

[6] Robak T, Blonski JZ, Robak P (2016). Antibody therapy alone and in combination with targeted drugs in chronic lymphocytic leukemia. Semin. Oncol..

[7] Burger JA (2020). Long-term efficacy and safety of first-line ibrutinib treatment for patients with CLL/SLL: 5 years of follow-up from the phase 3 RESONATE-2 study. Leukemia.

[8] Brown JR (2014). Idelalisib, an inhibitor of phosphatidylinositol 3-kinase p110δ, for relapsed/refractory chronic lymphocytic leukemia. Blood.

[9] Jain N (2019). Ibrutinib and venetoclax for first-line treatment of CLL. N. Engl. J. Med..

[10] Lampson BL (2016). Idelalisib given front-line for treatment of chronic lymphocytic leukemia causes frequent immune-mediated hepatotoxicity. Blood.

[11] Woyach JA (2017). BTKC481S-mediated resistance to ibrutinib in chronic lymphocytic leukemia. J. Clin. Oncol..

[12] Ahn IE (2017). Clonal evolution leading to ibrutinib resistance in chronic lymphocytic leukemia. Blood.

[13] Herling CD (2018). Clonal dynamics towards the development of venetoclax resistance in chronic lymphocytic leukemia. Nat. Commun..

[14] Ryan CE (2022). MAJIC: A phase III trial of acalabrutinib + venetoclax versus venetoclax + obinutuzumab in previously untreated chronic lymphocytic leukemia or small lymphocytic lymphoma. Future Oncol. Lond. Engl..

[15] Davids MS (2021). A phase 1b/2 study of duvelisib in combination with FCR (DFCR) for frontline therapy for younger CLL patients. Leukemia.

[16] Al-Sawaf O (2020). Venetoclax plus obinutuzumab versus chlorambucil plus obinutuzumab for previously untreated chronic lymphocytic leukaemia (CLL14): Follow-up results from a multicentre, open-label, randomised, phase 3 trial. Lancet Oncol..

[17] Kater AP (2020). Venetoclax plus rituximab in relapsed chronic lymphocytic leukemia: 4-year results and evaluation of impact of genomic complexity and gene mutations from the MURANO phase III study. J. Clin. Oncol..

[18] Staber PB (2021). Tafasitamab combined with idelalisib or venetoclax in patients with CLL previously treated with a BTK inhibitor. Leuk. Lymphoma.

[19] Ghia P (2020). ASCEND: Phase III, randomized trial of acalabrutinib versus idelalisib plus rituximab or bendamustine plus rituximab in relapsed or refractory chronic lymphocytic leukemia. J. Clin. Oncol..

[20] Black GS (2022). Subclonal evolution of CLL driver mutations is associated with relapse in ibrutinib- and acalabrutinib-treated patients. Blood.

[21] Zapatka M (2022). Clonal evolution in chronic lymphocytic leukemia is scant in relapsed but accelerated in refractory cases after chemo(immune) therapy. Haematologica.

[22] Abbaci A (2018). Neurotensin receptor type 2 protects B-cell chronic lymphocytic leukemia cells from apoptosis. Oncogene.

[23] Kostopoulou F (2019). Gain of the short arm of chromosome 2 (2p gain) has a significant role in drug-resistant chronic lymphocytic leukemia. Cancer Med..

[24] Saada S (2012). Differential expression of neurotensin and specific receptors, NTSR1 and NTSR2, in normal and malignant human B lymphocytes. J. Immunol..

[25] Libanje F (2023). NTSR1 glycosylation and MMP dependent cleavage generate three distinct forms of the protein. Sci. Rep..

[26] *NTSR2 (Human)*. https://www.phosphosite.org/proteinAction?id=3128700&showAllSites=true#appletMsg.

[27] Lee C-R, Park Y-H, Kim Y-R, Peterkofsky A, Seok Y-J (2013). Phosphorylation-dependent mobility shift of proteins on SDS-PAGE is due to decreased binding of SDS. Bull. Korean Chem. Soc..

[28] Moya-Alvarado G (2023). BDNF/TrkB signaling endosomes in axons coordinate CREB/mTOR activation and protein synthesis in the cell body to induce dendritic growth in cortical neurons. eLife.

[29] Patel SG (2019). Cell-penetrating peptide sequence and modification dependent uptake and subcellular distribution of green florescent protein in different cell lines. Sci. Rep..

[30] Nguyen P-H, Reinart N, Hallek M (2013). Role of lyn kinase in the pathogenesis of chronic lymphocytic leukemia. Blood.

[31] Baliakas P (2019). Cytogenetic complexity in chronic lymphocytic leukemia: Definitions, associations, and clinical impact. Blood.

[32] Bhanumathy K (2021). Protein tyrosine kinases: Their roles and their targeting in leukemia. Cancers.

[33] Alvarez RH, Kantarjian HM, Cortes JE (2006). The role of Src in solid and hematologic malignancies. Cancer.

[34] Nguyen P-H (2016). LYN kinase in the tumor microenvironment is essential for the progression of chronic lymphocytic leukemia. Cancer Cell.

[35] Woyach JA (2014). Bruton’s tyrosine kinase (BTK) function is important to the development and expansion of chronic lymphocytic leukemia (CLL). Blood.

[36] Li CM (2021). Novel peptide therapeutic approaches for cancer treatment. Cells.

[37] Pramil E (2019). Targeting chronic lymphocytic leukemia with N-methylated thrombospondin-1-derived peptides overcomes drug resistance. Blood Adv..

[38] Simon-Gracia L (2020). Bifunctional therapeutic peptides for targeting malignant B cells and hepatocytes: Proof of concept in chronic lymphocytic leukemia. Adv. Ther..

[39] Rossi D (2013). Association between molecular lesions and specific B-cell receptor subsets in chronic lymphocytic leukemia. Blood.

[40] Nguyen-Khac F, Balogh Z, Chauzeix J, Veronese L, Chapiro E (2023). Cytogenetics in the management of chronic lymphocytic leukemia: Guidelines from the Groupe Francophone de Cytogénétique Hématologique (GFCH). Curr. Res. Transl. Med..

[41] Lanigan TM (2020). Real time visualization of cancer cell death, survival and proliferation using fluorochrome-transfected cells in an IncuCyte® imaging system. J. Biol. Methods.

[42] Gelles JD, Chipuk JE (2016). Robust high-throughput kinetic analysis of apoptosis with real-time high-content live-cell imaging. Cell Death Dis..

