# Supplementary material for: Targeting the NTSR2/TrkB oncogenic pathway in chronic lymphocytic leukemia
Source: Sci Rep. 2024 Mar 13;14:6084. doi: 10.1038/s41598-024-56663-5 (PMC10937676; doi:10.1038/s41598-024-56663-5)

Patient 1

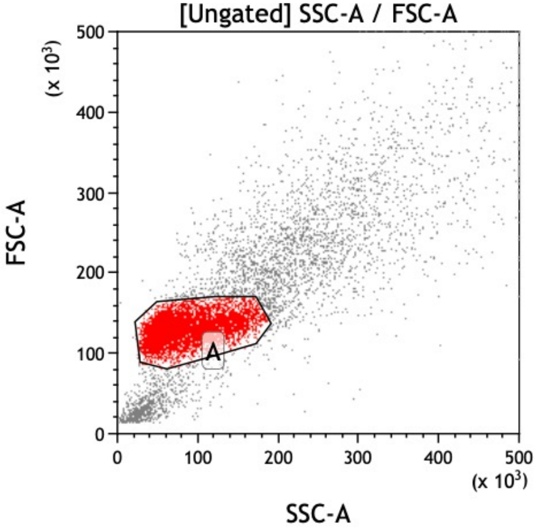

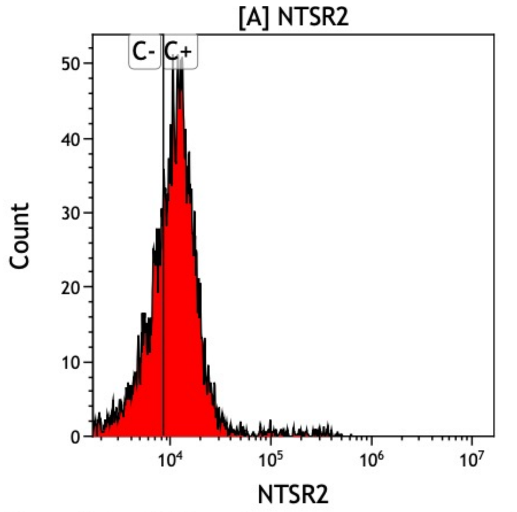

Gate	%Total	%Gated
All	62,22	100,00
C-	23,05	37,05
C+	39,17	62,95

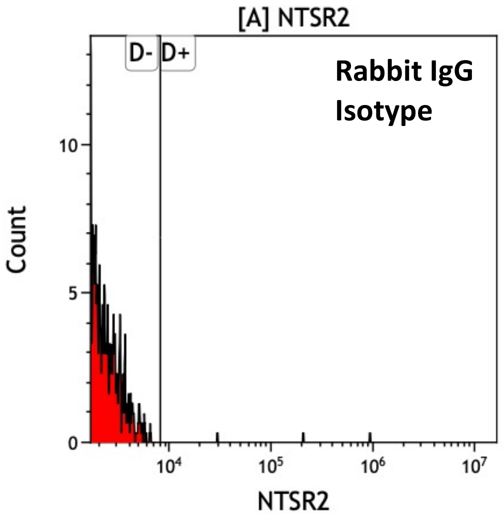

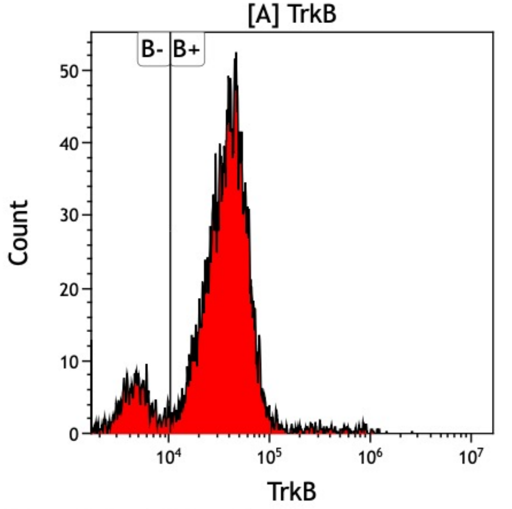

Gate	%Total	%Gated
All	62,22	100,00
B-	7,31	11,75
B+	54,91	88,25

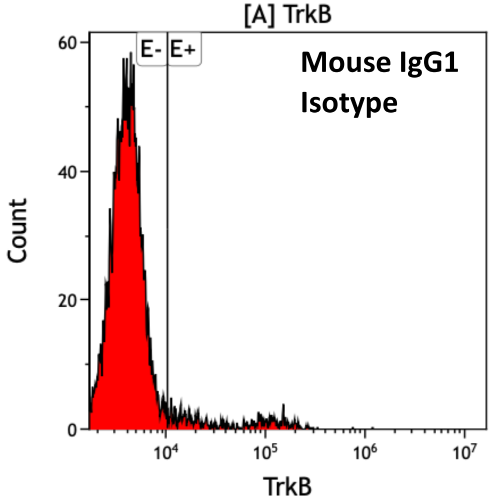

Patient 2

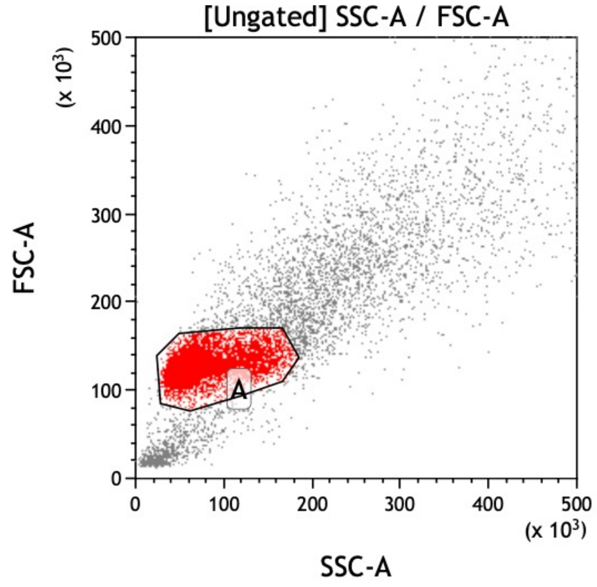

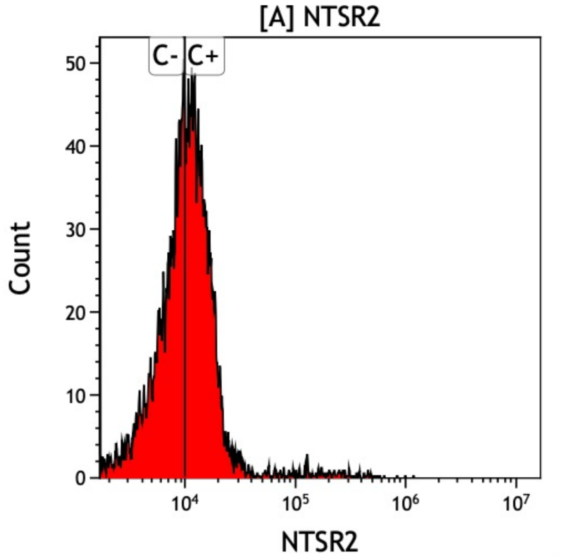

Gate	%Total	%Gated
All	58,49	100,00
C-	27,43	46,90
C+	31,06	53,10

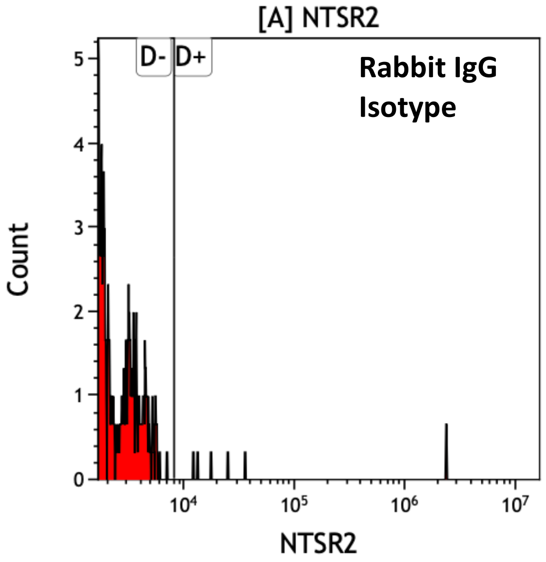

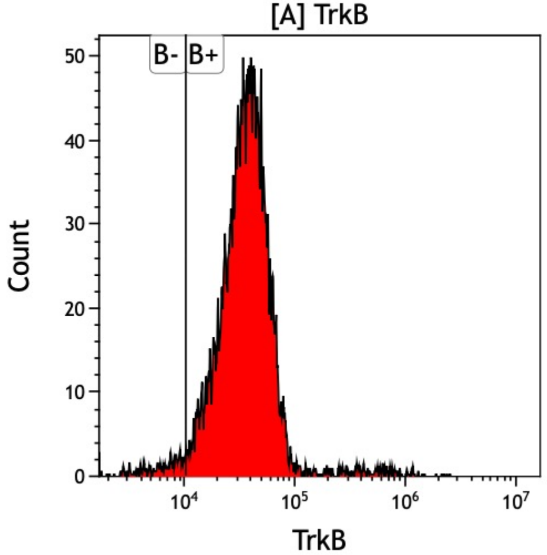

Gate	%Total	%Gated
All	58,49	100,00
B-	1,83	3,13
B+	56,66	96,87

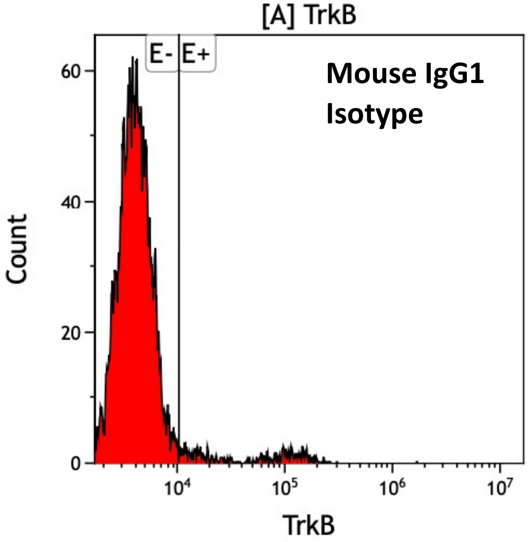

Patient 3

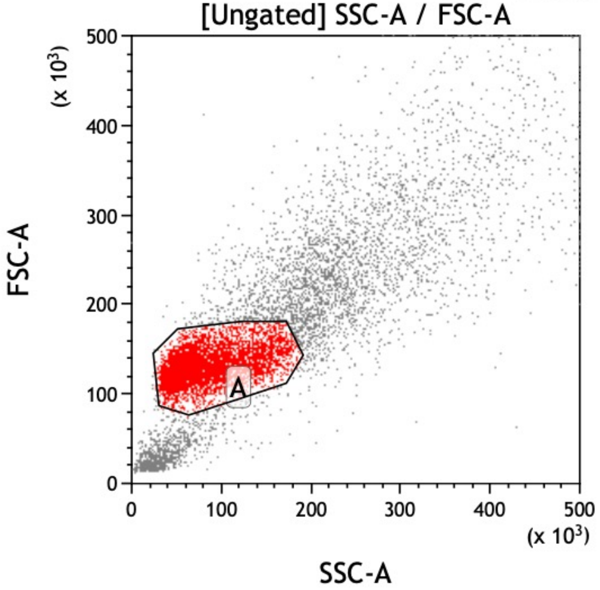

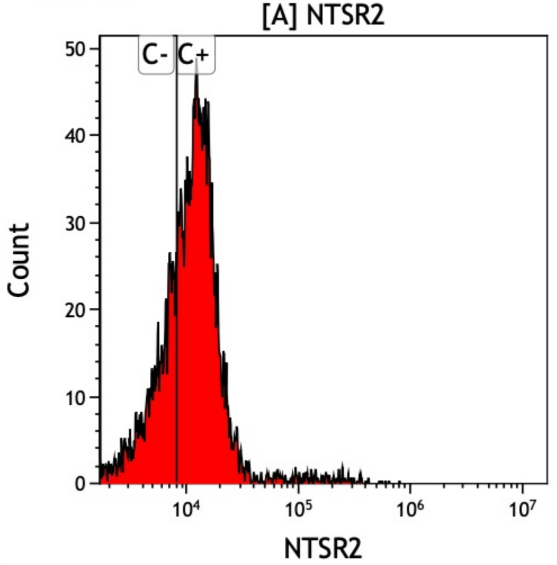

Gate	%Total	%Gated
All	56,27	100,00
C-	16,73	29,73
C+	39,54	70,27

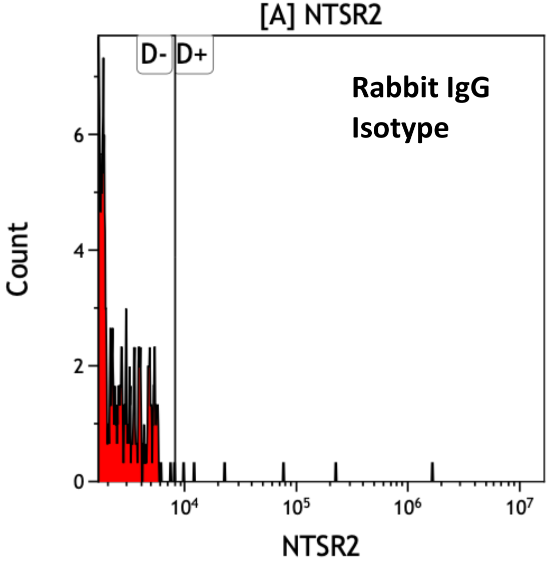

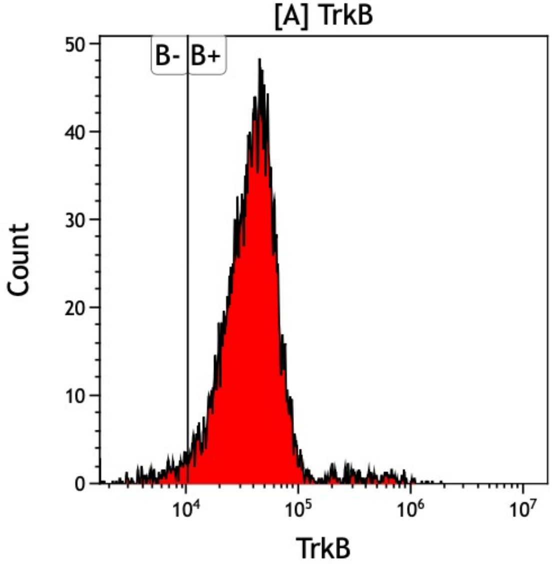

Gate	%Total	%Gated
All	56,27	100,00
B-	1,82	3,23
B+	54,45	96,77

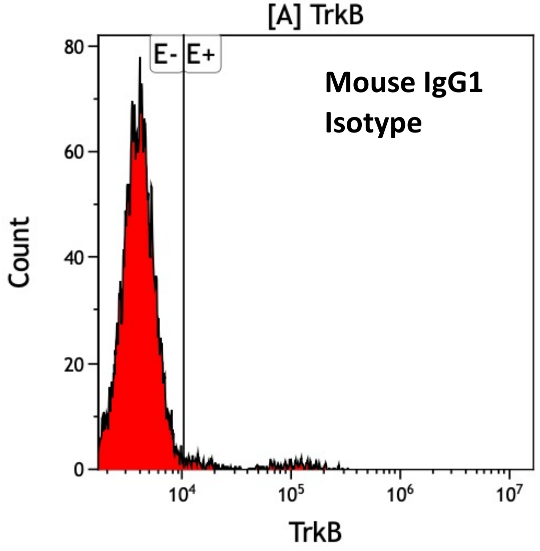

Patient 4

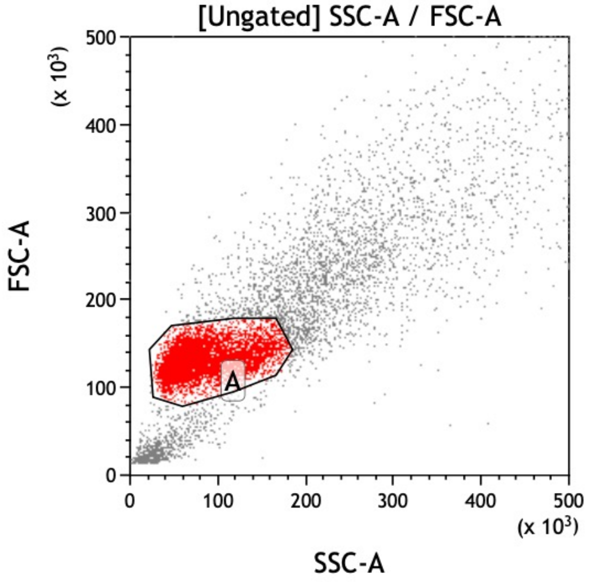

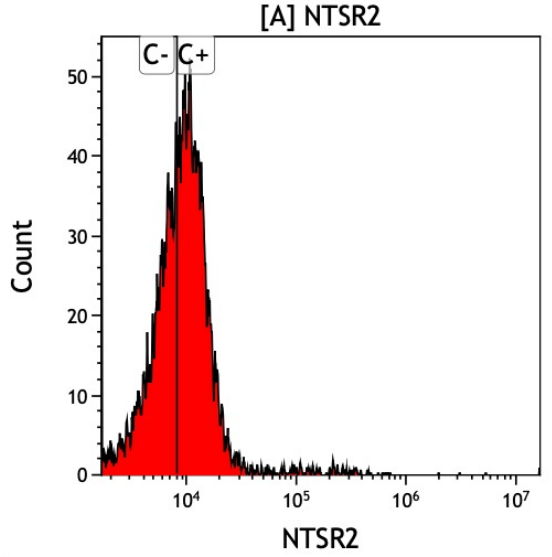

Gate	%Total	%Gated
All	61,07	100,00
C-	24,76	40,54
C+	36,31	59,46

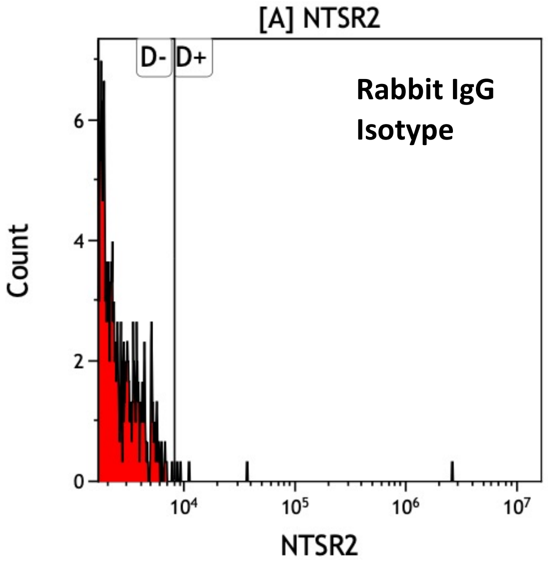

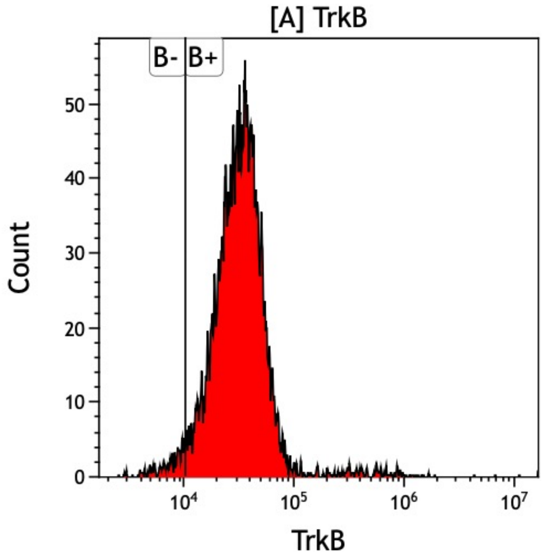

Gate	%Total	%Gated
All	61,07	100,00
B-	1,97	3,23
B+	59,10	96,77

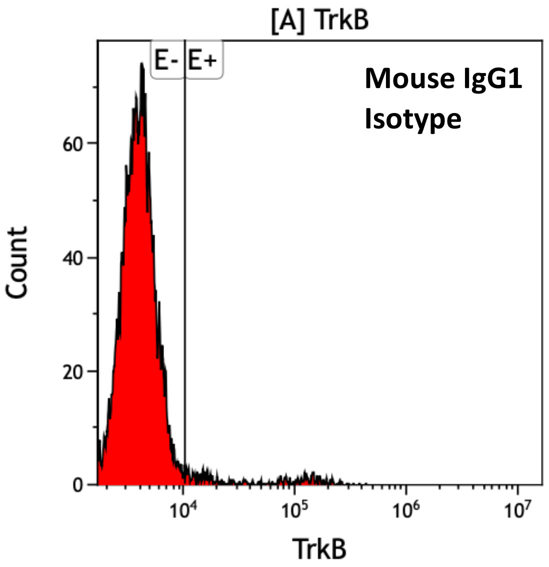

Patient 5

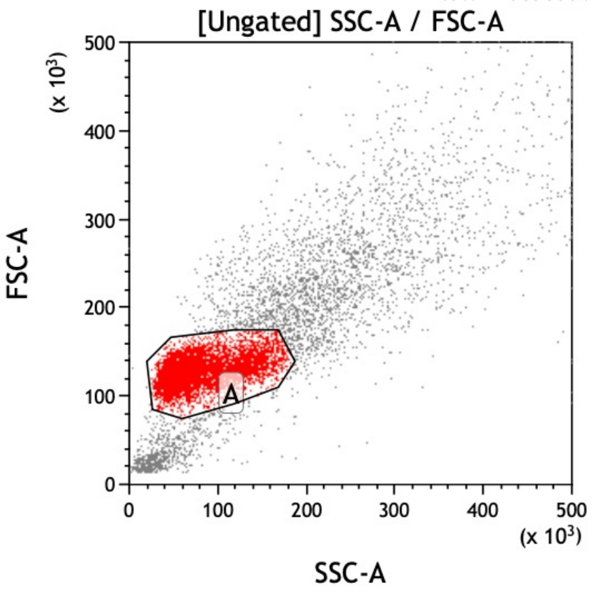

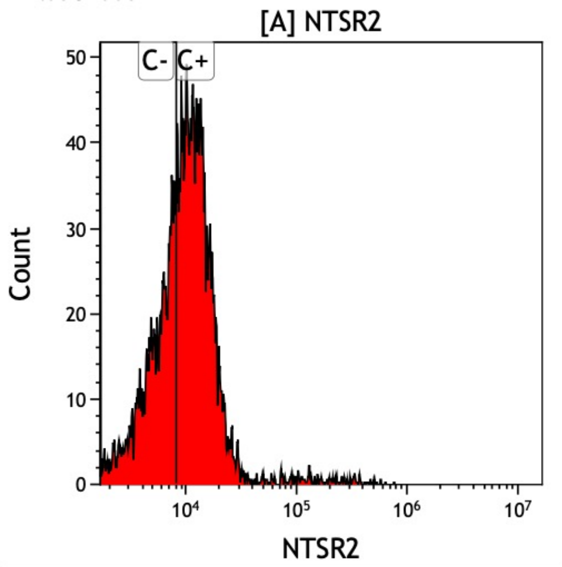

Gate	%Total	%Gated
All	61,98	100,00
C-	22,46	36,24
C+	39,52	63,76

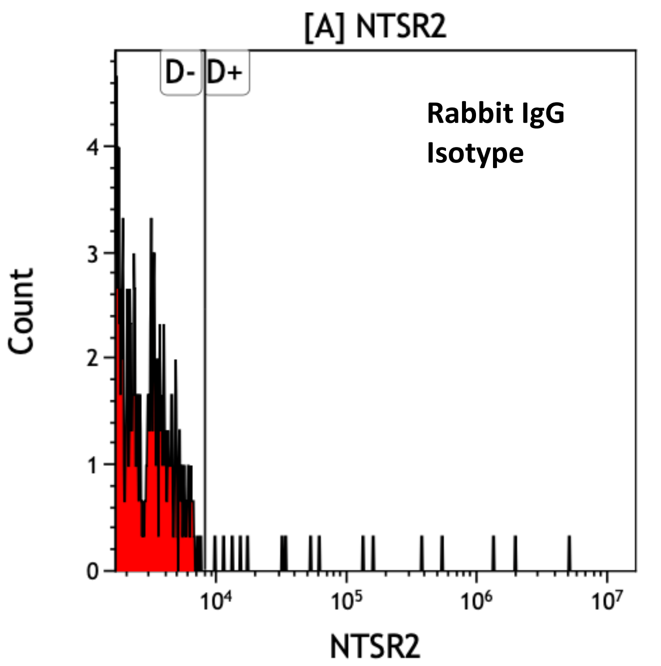

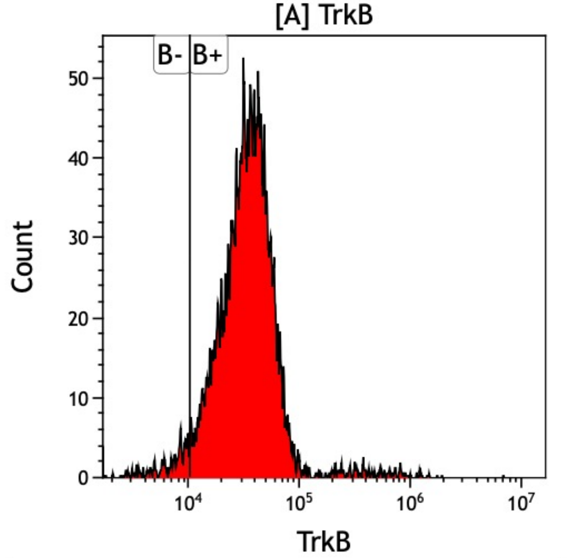

Gate	%Total	%Gated
All	61,98	100,00
B-	2,53	4,08
B+	59,45	95,92

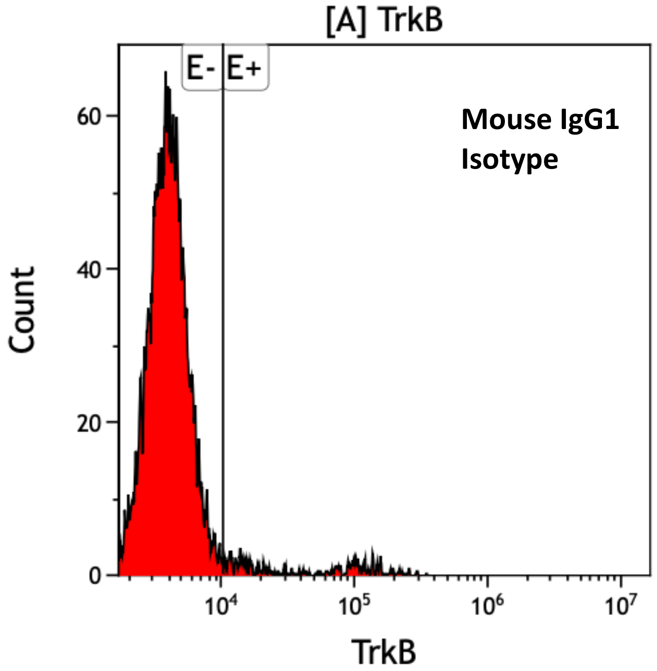

Patient 6

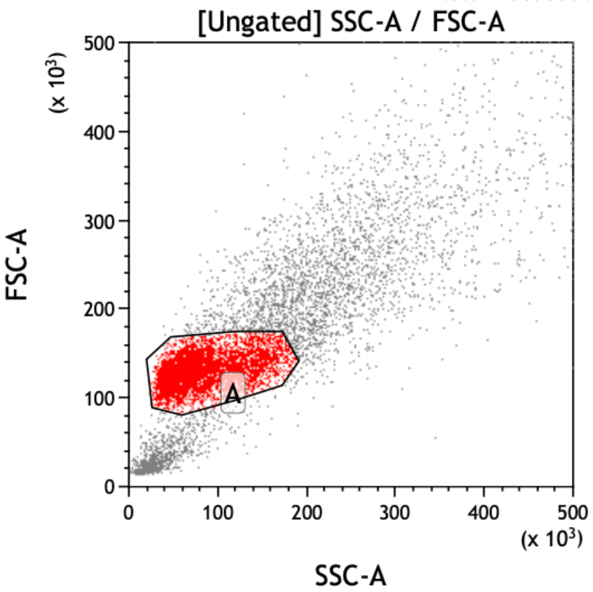

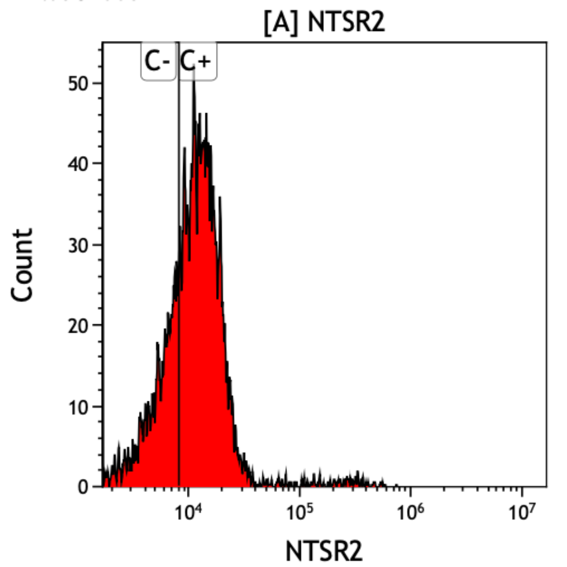

Gate	%Total	%Gated
All	60,28	100,00
C-	17,08	28,33
C+	43,20	71,67

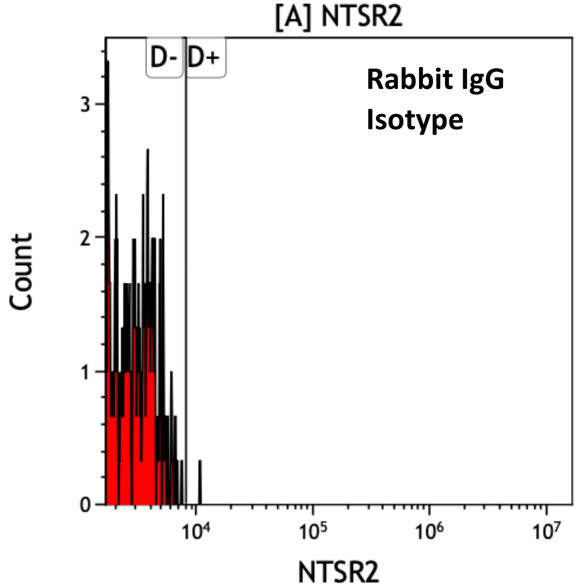

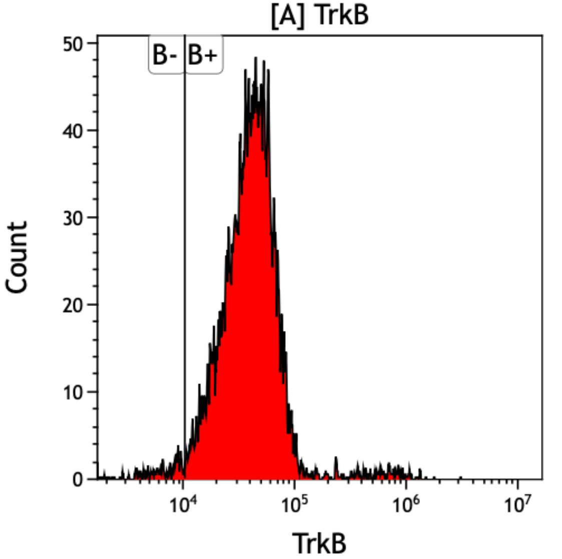

Gate	%Total	%Gated
All	60,28	100,00
B-	1,47	2,44
B+	58,81	97,56

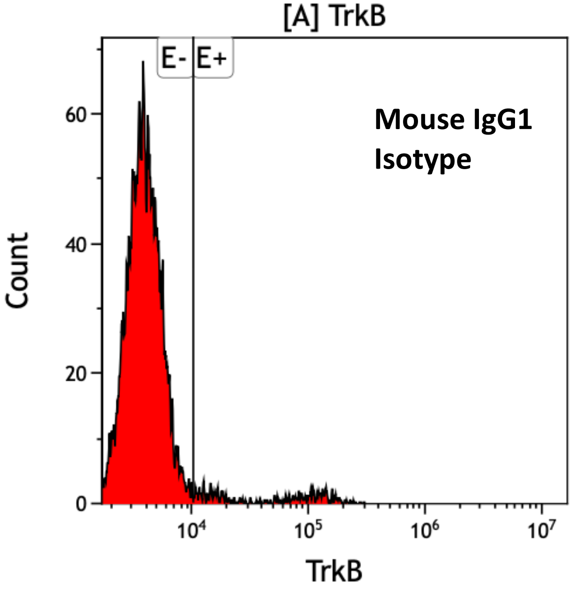

Patient 7

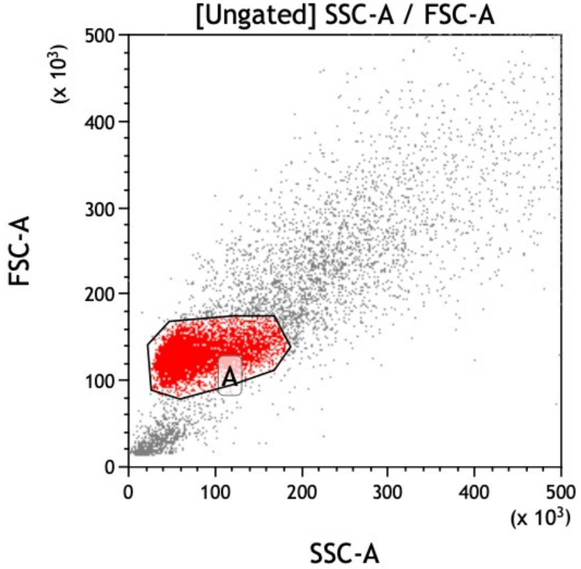

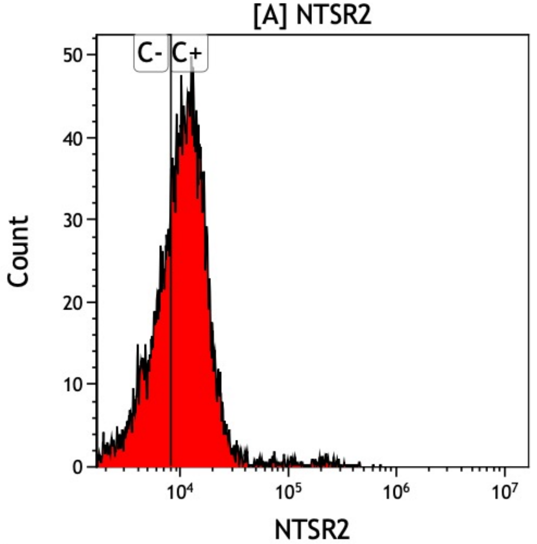

Gate	%Total	%Gated
All	60,37	100,00
C-	18,93	31,36
C+	41,44	68,64

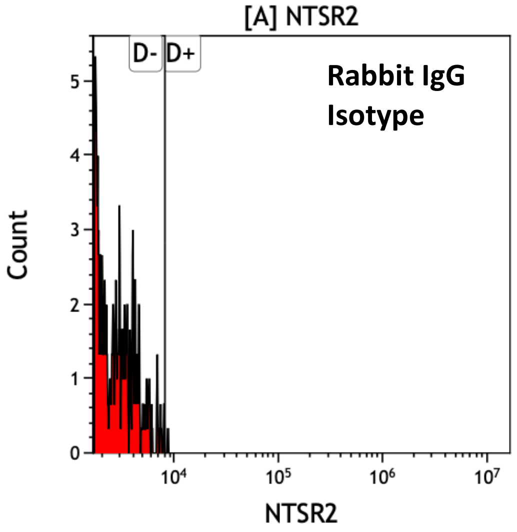

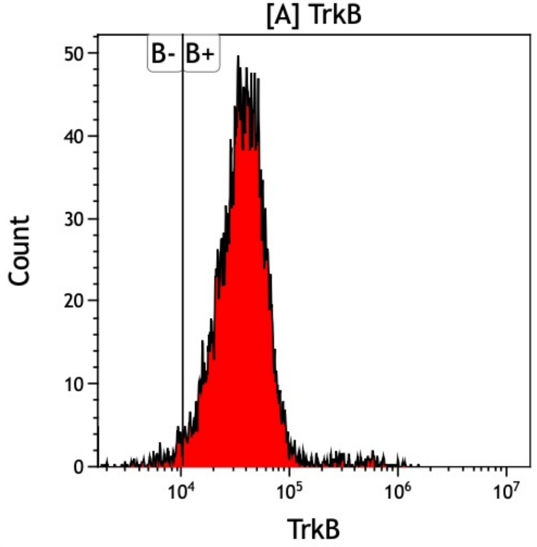

Gate	%Total	%Gated
All	60,37	100,00
B-	1,55	2,57
B+	58,82	97,43

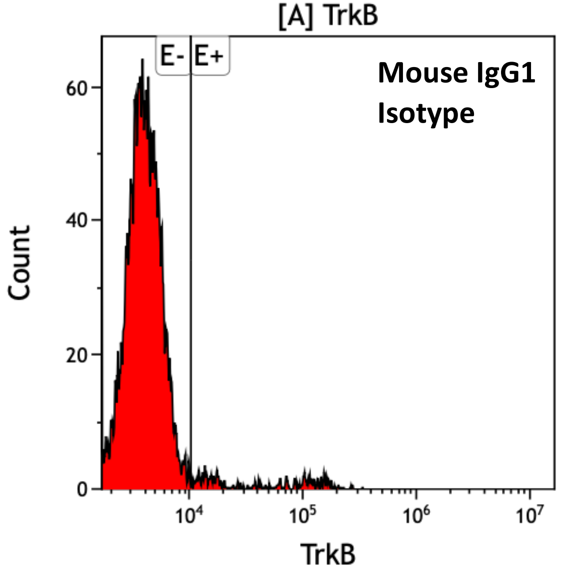

Patient 8

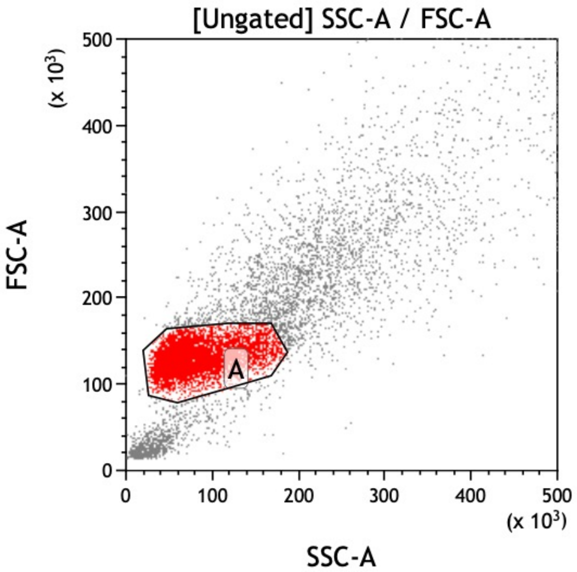

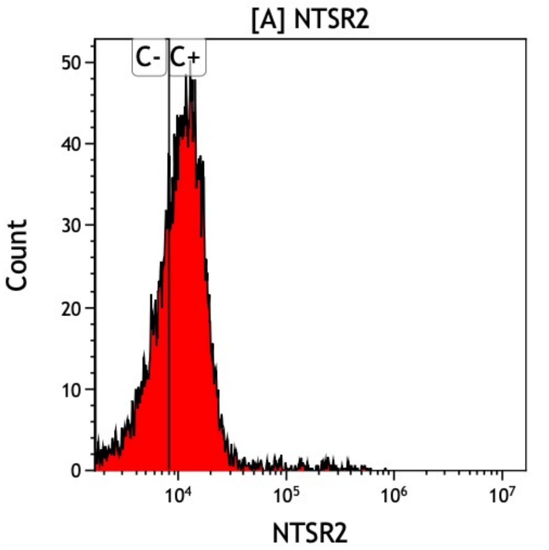

Gate	%Total	%Gated
All	60,20	100,00
C-	18,26	30,33
C+	41,94	69,67

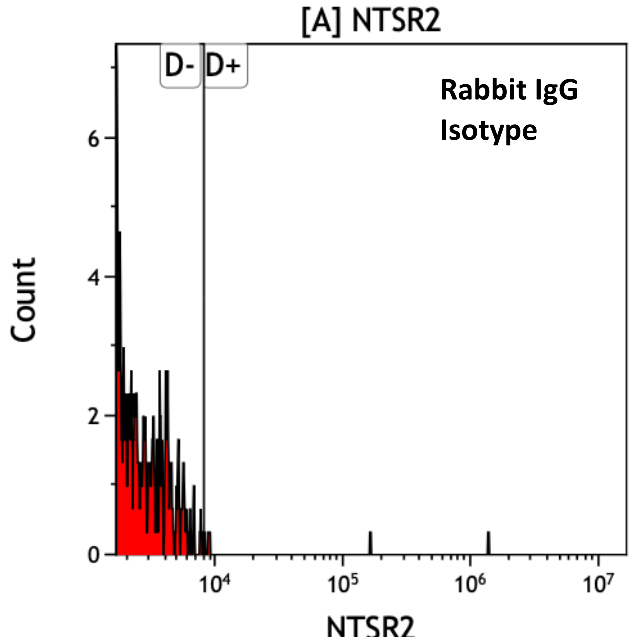

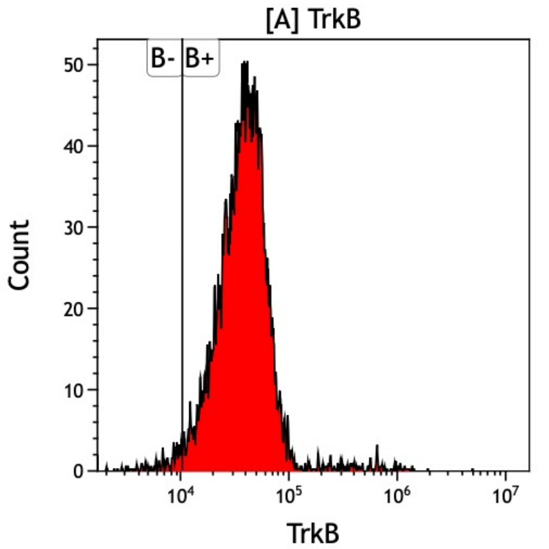

Gate	%Total	%Gated
All	60,20	100,00
B-	1,37	2,28
B+	58,83	97,72

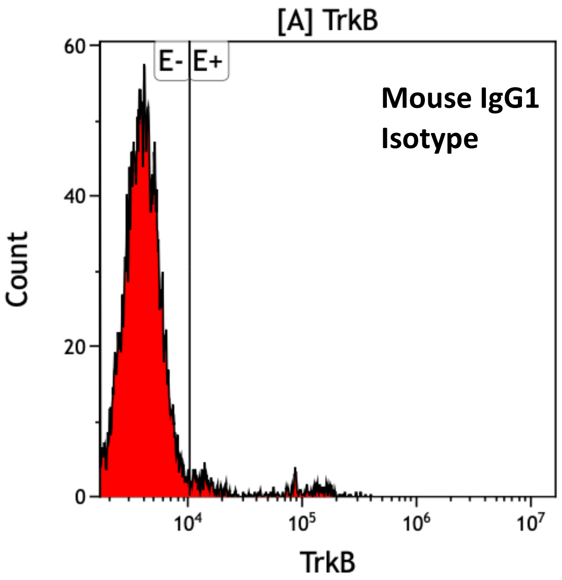

Patient 9

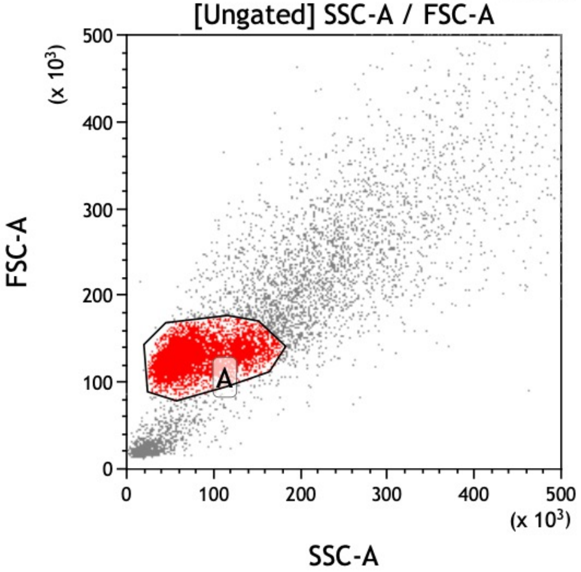

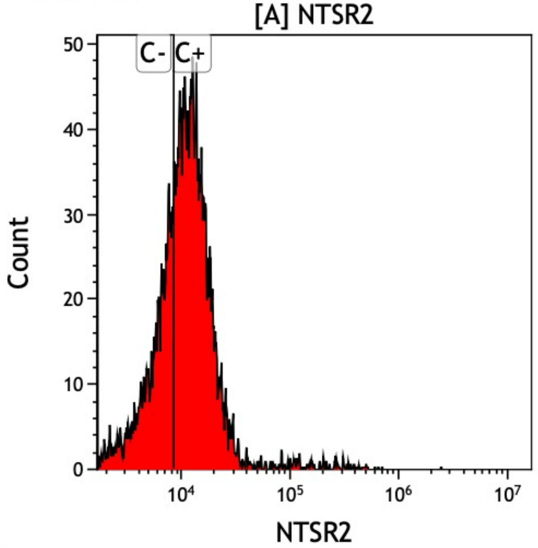

Gate	%Total	%Gated
All	57,91	100,00
C-	18,53	32,00
C+	39,38	68,00

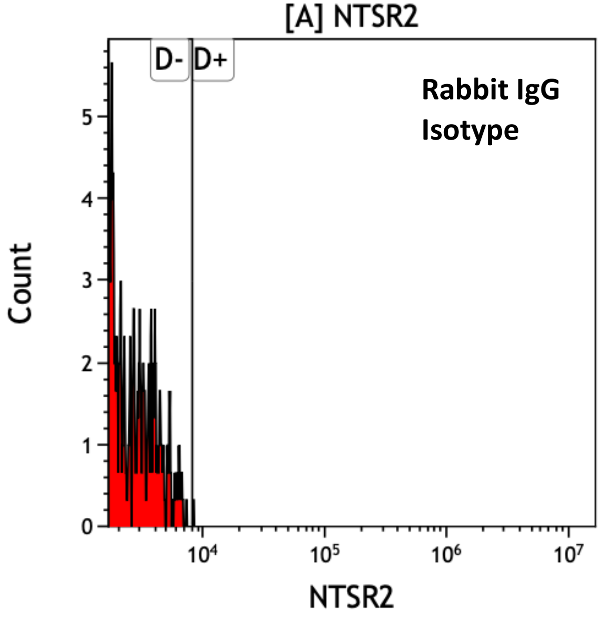

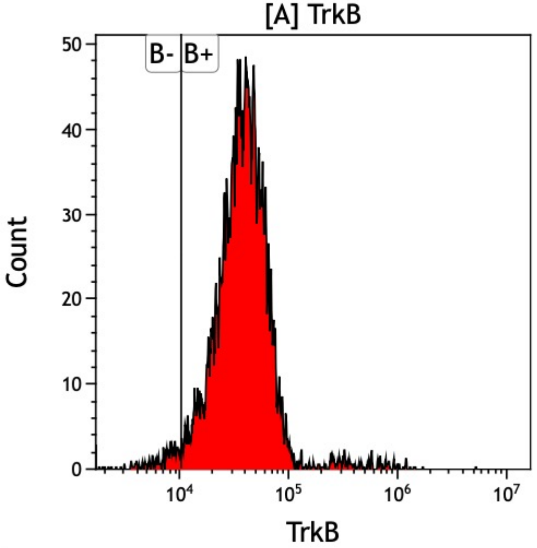

Gate	%Total	%Gated
All	57,91	100,00
B-	1,53	2,64
B+	56,38	97,36

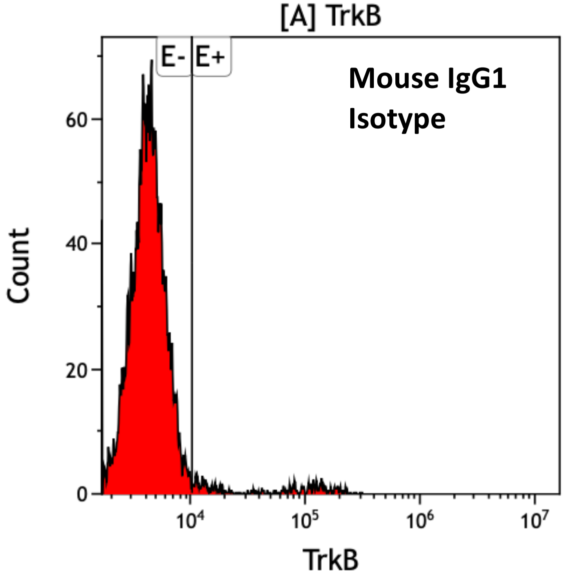

Patient 10

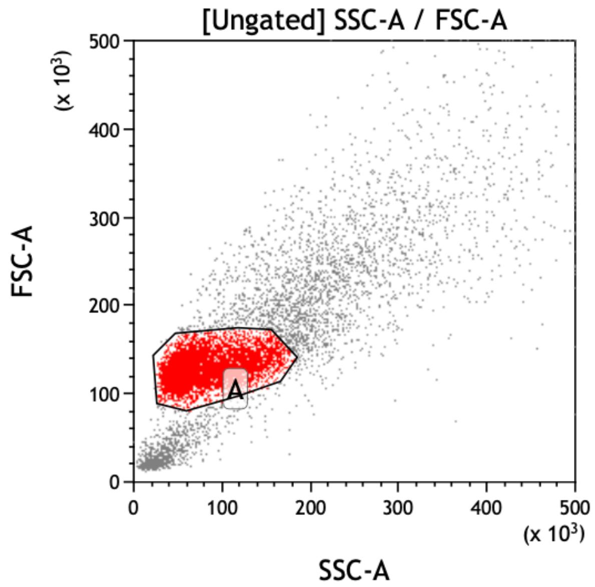

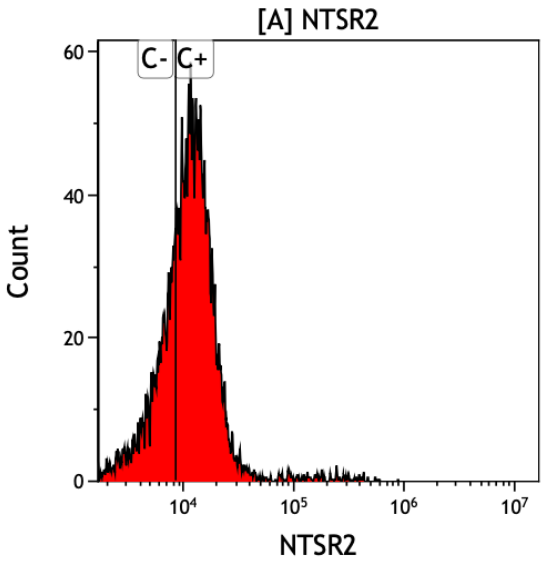

Gate	%Total	%Gated
All	64,14	100,00
C-	18,50	28,84
C+	45,64	71,16

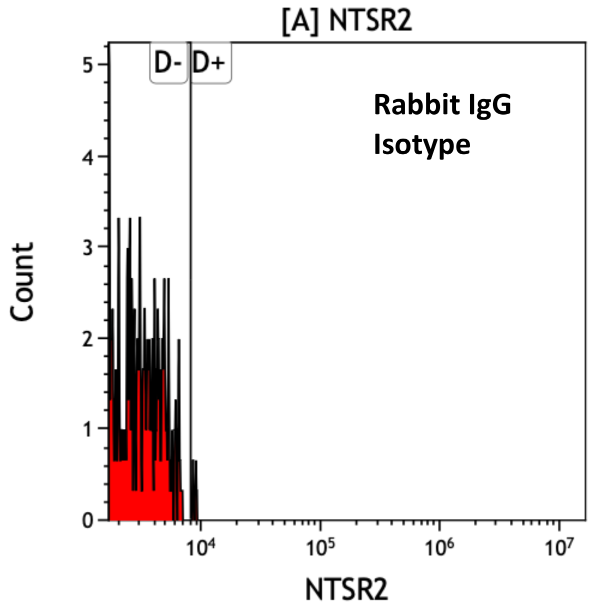

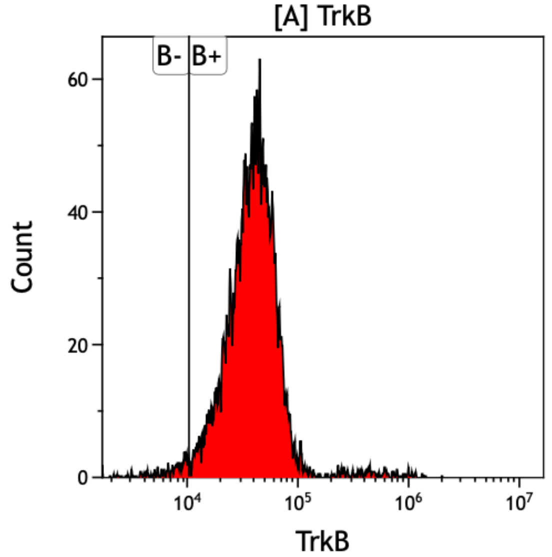

Gate	%Total	%Gated
All	64,14	100,00
B-	1,56	2,43
B+	62,58	97,57

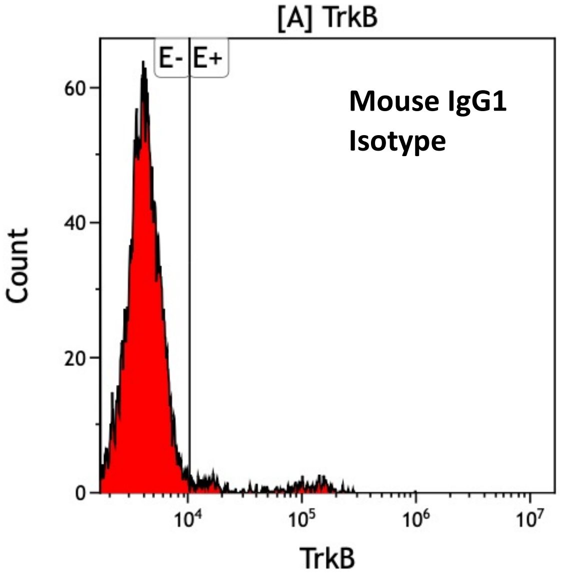

Patient 11

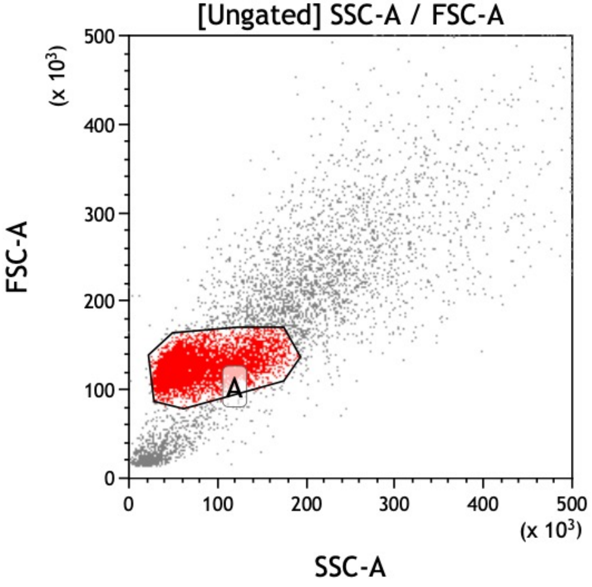

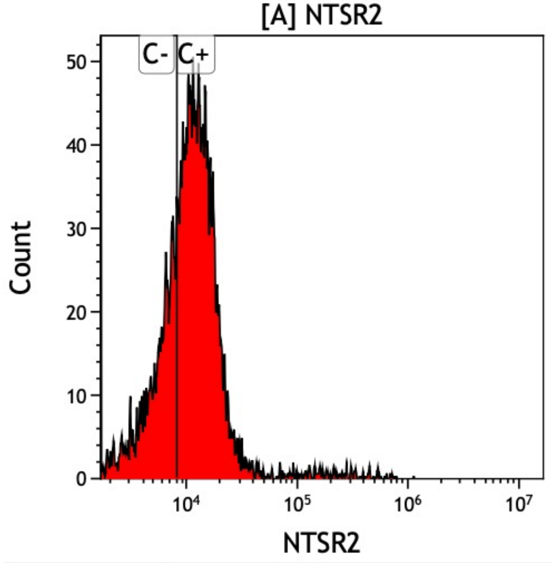

Gate	%Total	%Gated
All	62,28	100,00
C-	18,26	29,32
C+	44,02	70,68

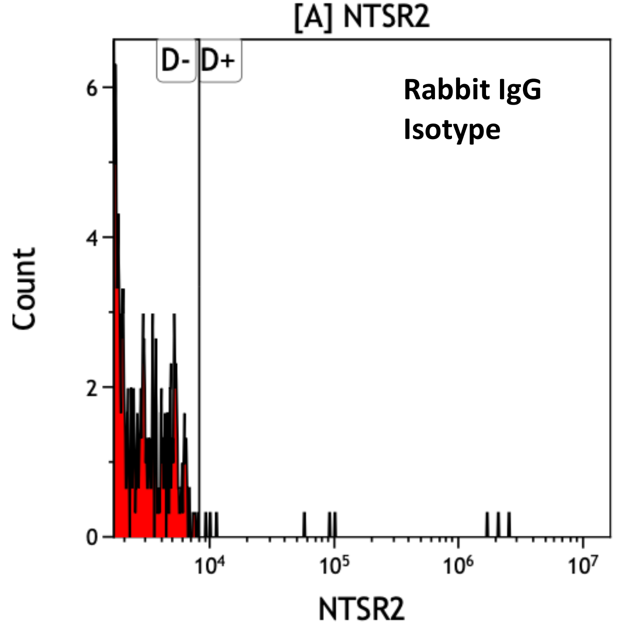

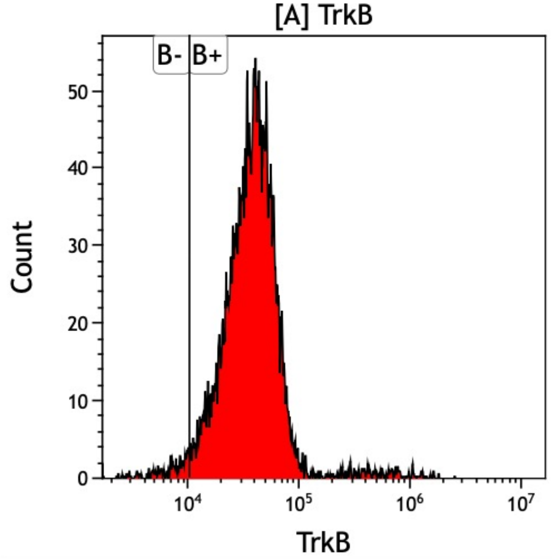

Gate	%Total	%Gated
All	62,28	100,00
B-	1,69	2,71
B+	60,59	97,29

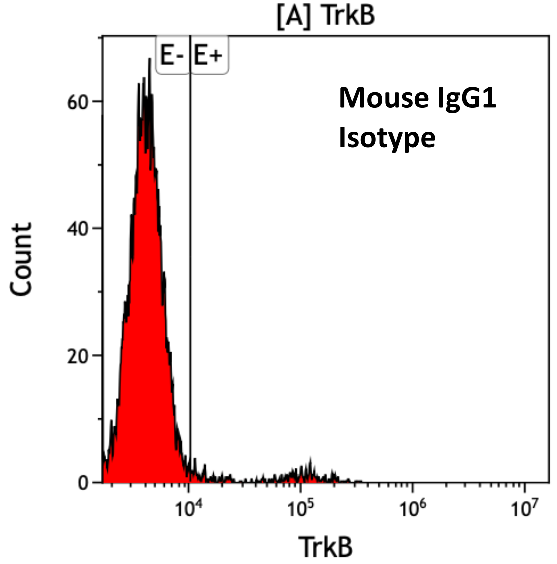

Patient 12

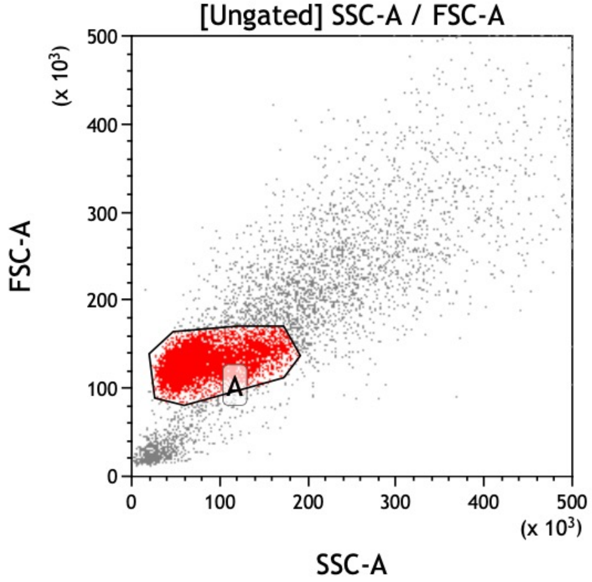

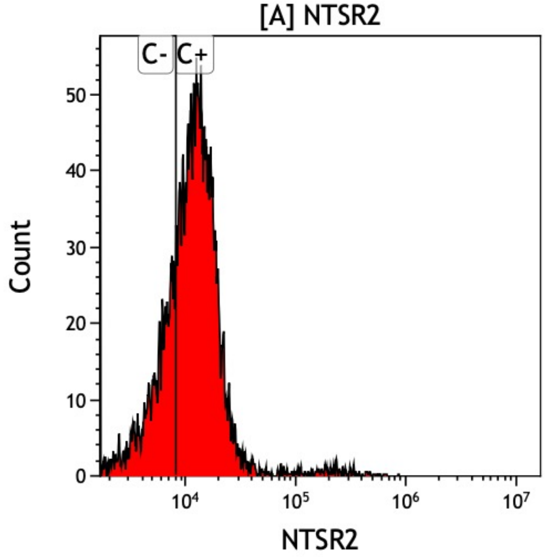

Gate	%Total	%Gated
All	63,63	100,00
C-	16,57	26,04
C+	47,06	73,96

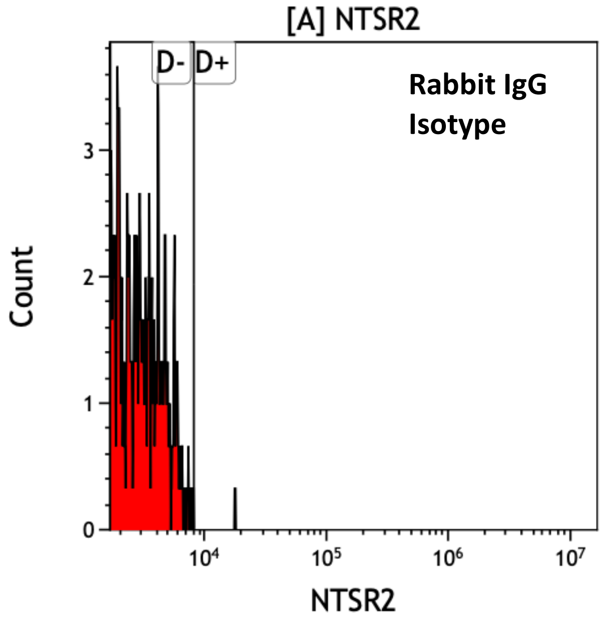

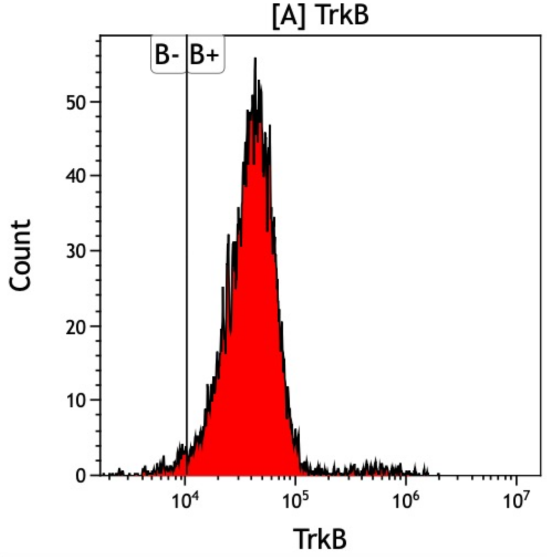

Gate	%Total	%Gated
All	63,63	100,00
B-	1,64	2,58
B+	61,99	97,42

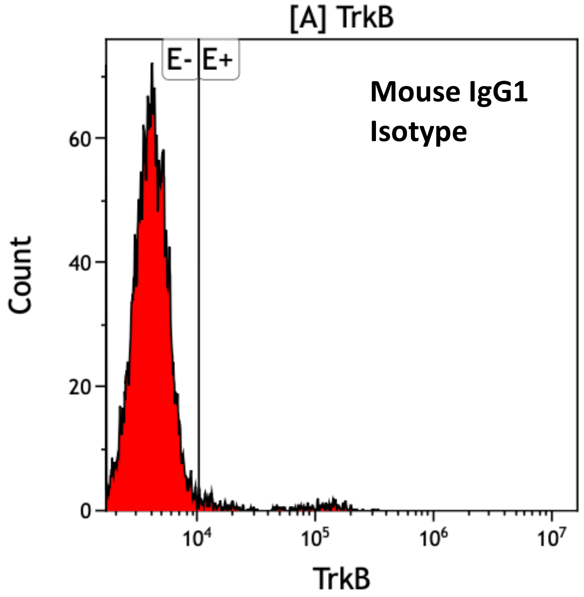

Patient 13

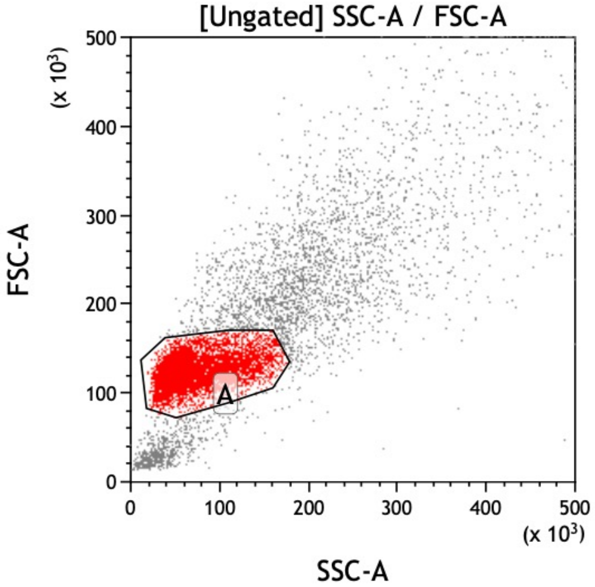

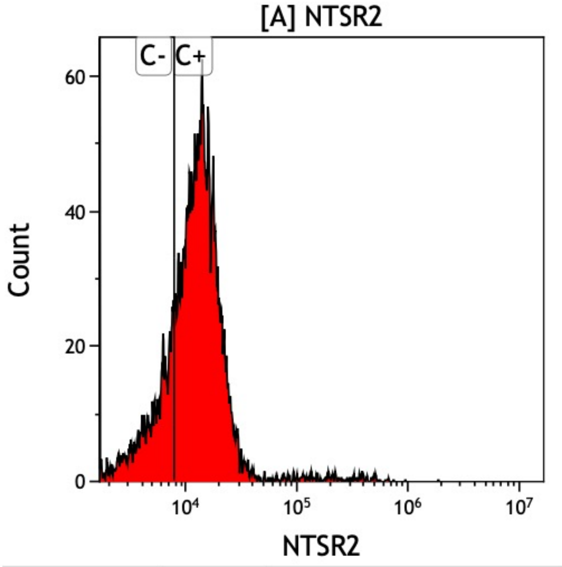

Gate	%Total	%Gated
All	65,29	100,00
C-	14,18	21,72
C+	51,11	78,28

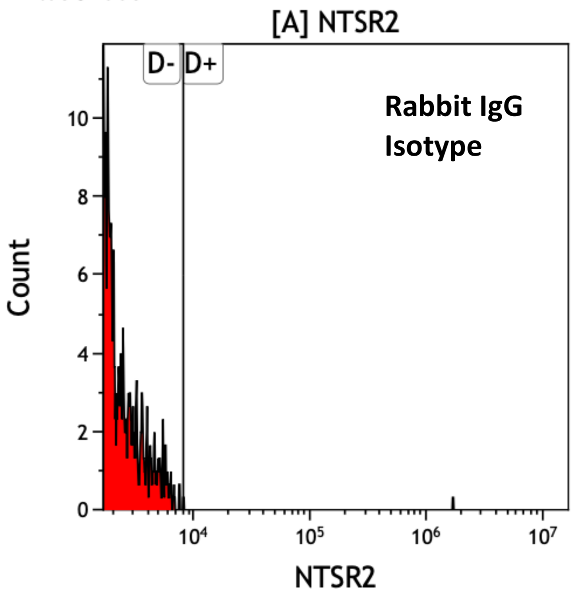

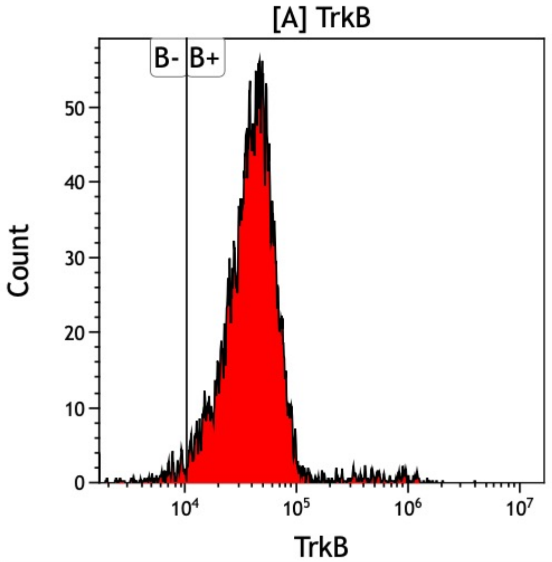

Gate	%Total	%Gated
All	65,29	100,00
B-	1,56	2,39
B+	63,73	97,61

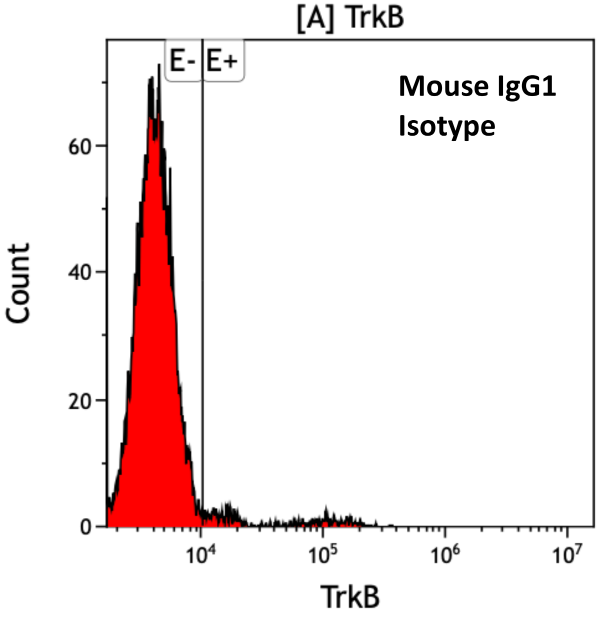

Patient 14

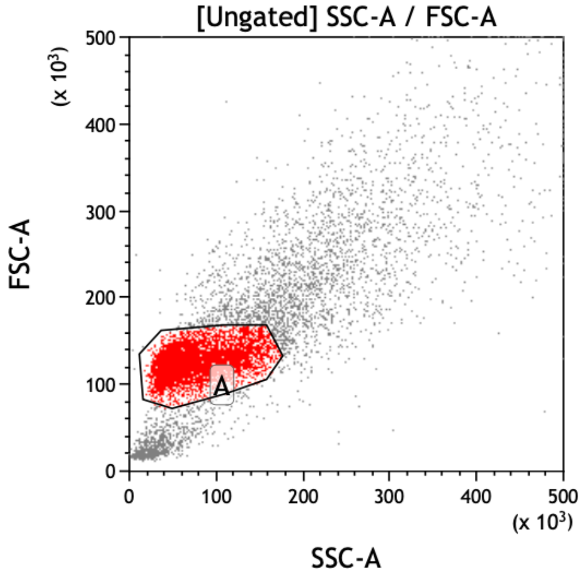

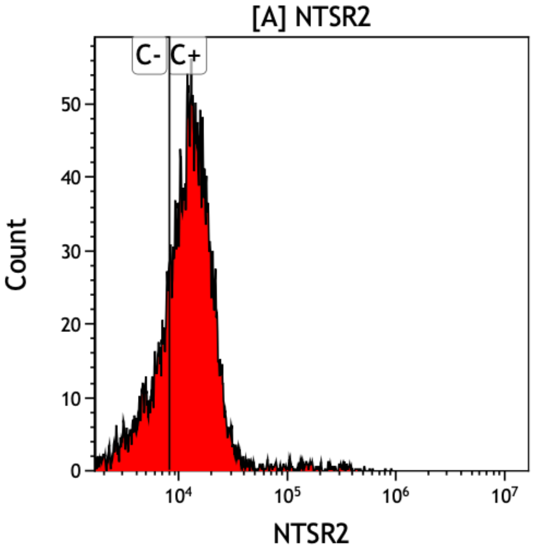

Gate	%Total	%Gated
All	63,29	100,00
C-	15,08	23,83
C+	48,21	76,17

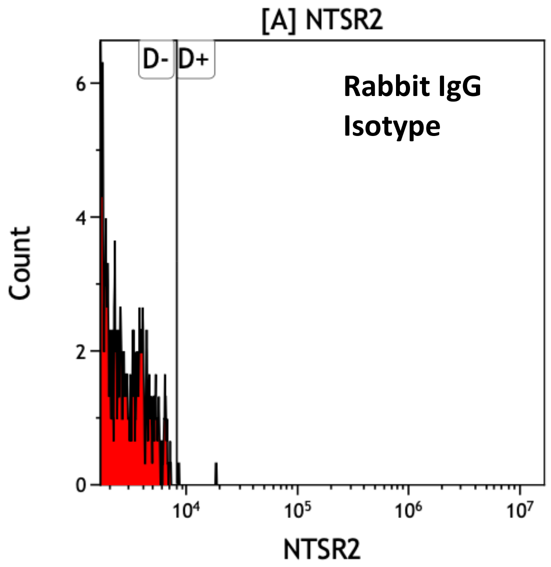

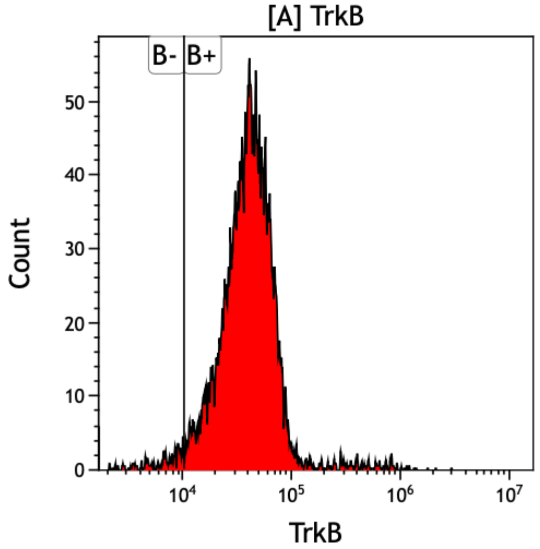

Gate	%Total	%Gated
All	63,29	100,00
B-	1,85	2,92
B+	61,44	97,08

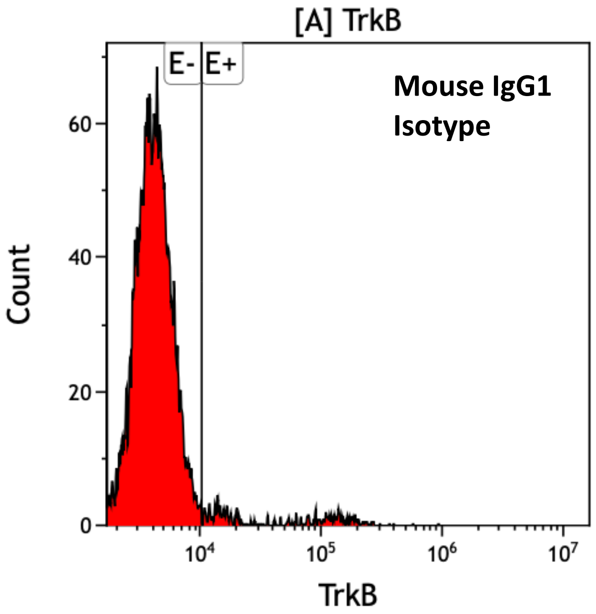

Patient 15

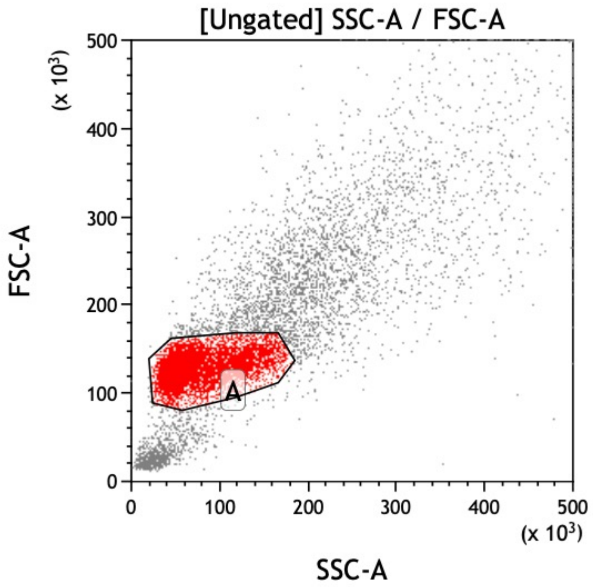

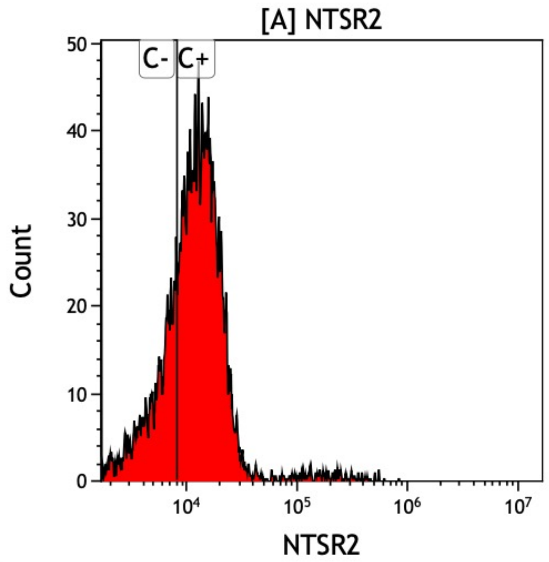

Gate	%Total	%Gated
All	57,62	100,00
C-	15,20	26,38
C+	42,42	73,62

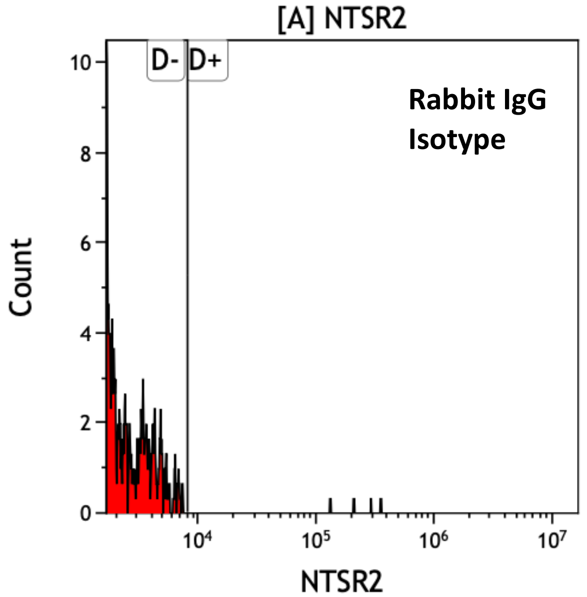

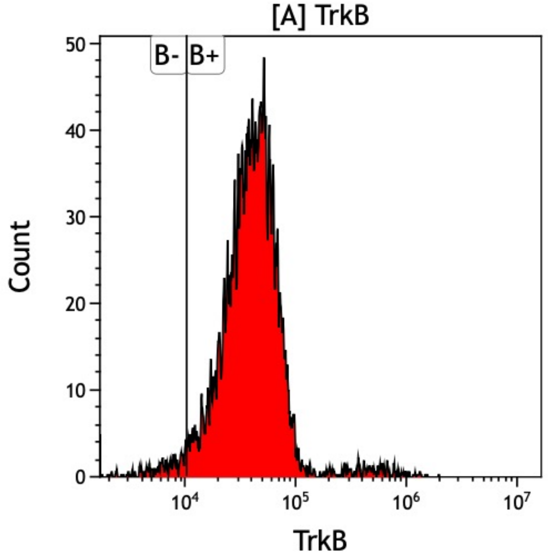

Gate	%Total	%Gated
All	57,62	100,00
B-	1,81	3,14
B+	55,81	96,86

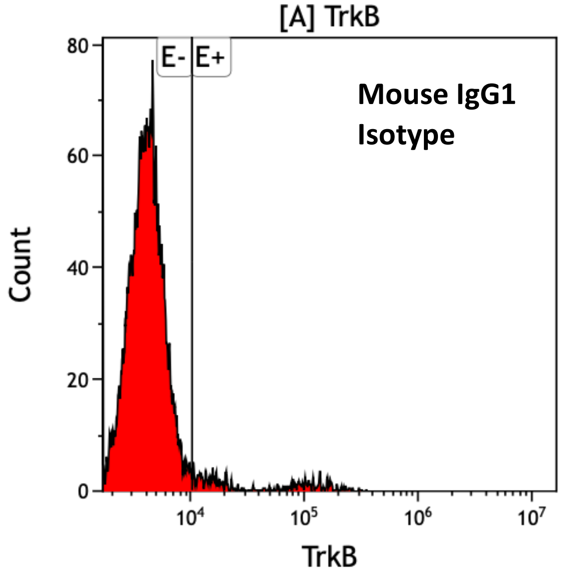

Patient 16

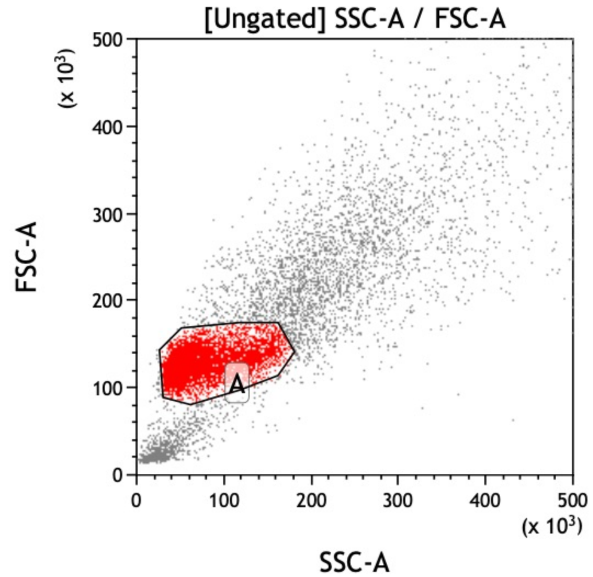

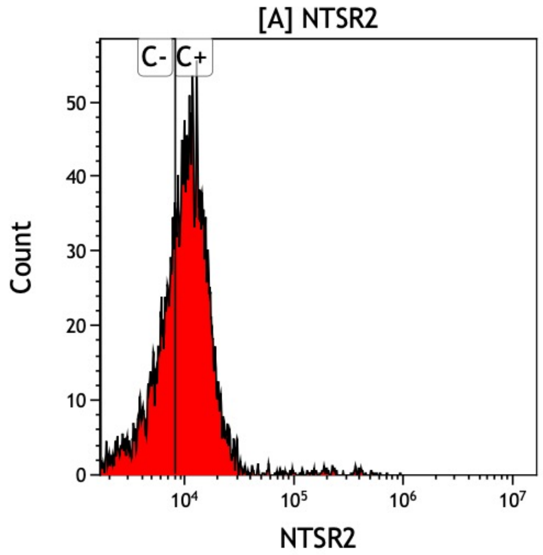

Gate	%Total	%Gated
All	56,78	100,00
C-	18,60	32,76
C+	38,18	67,24

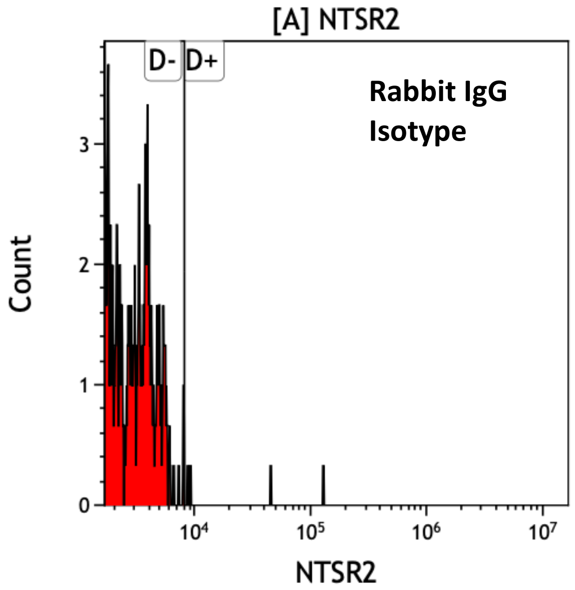

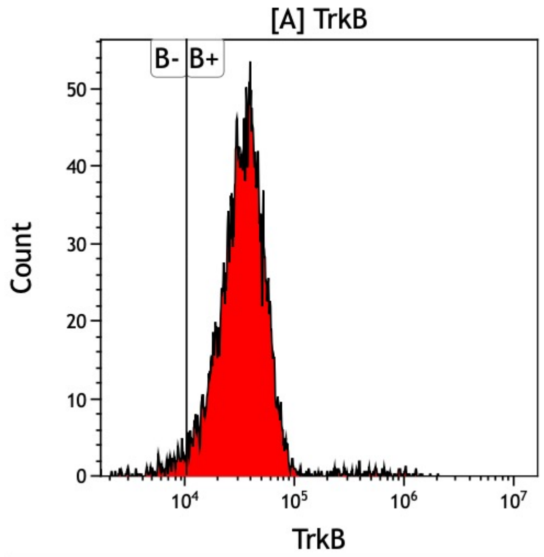

Gate	%Total	%Gated
All	56,78	100,00
B-	1,77	3,12
B+	55,01	96,88

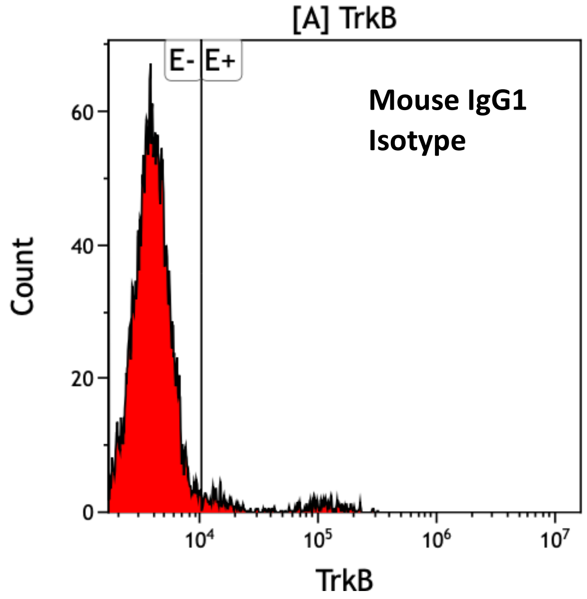

Patient 17

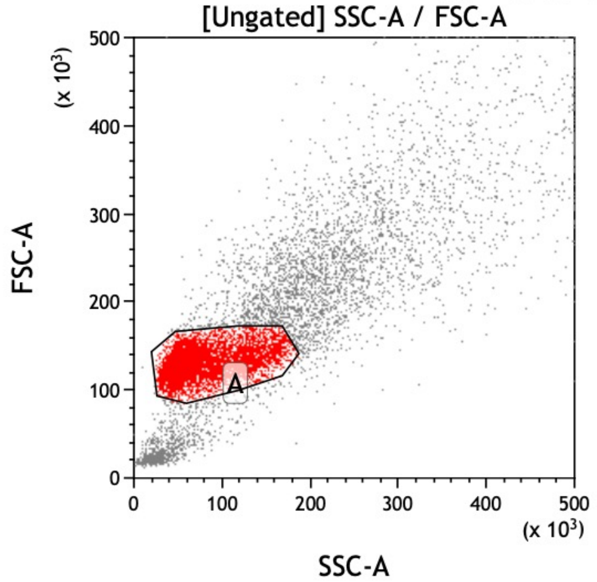

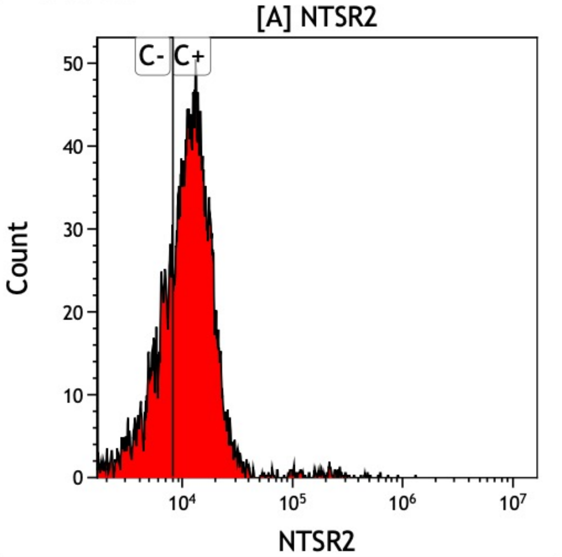

Gate	%Total	%Gated
All	57,86	100,00
C-	16,22	28,03
C+	41,64	71,97

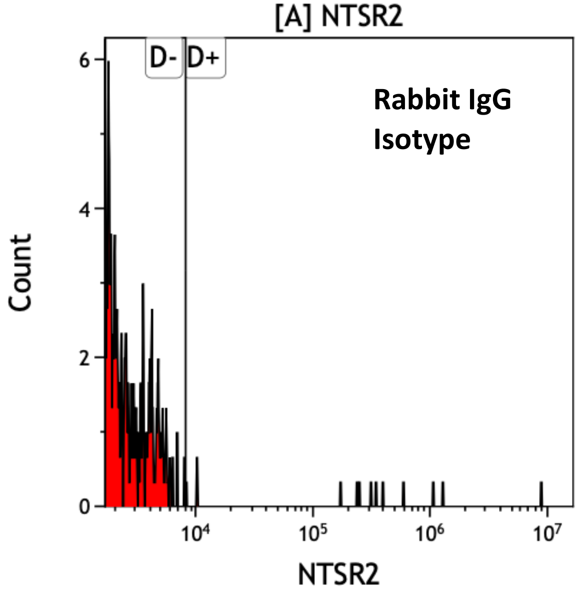

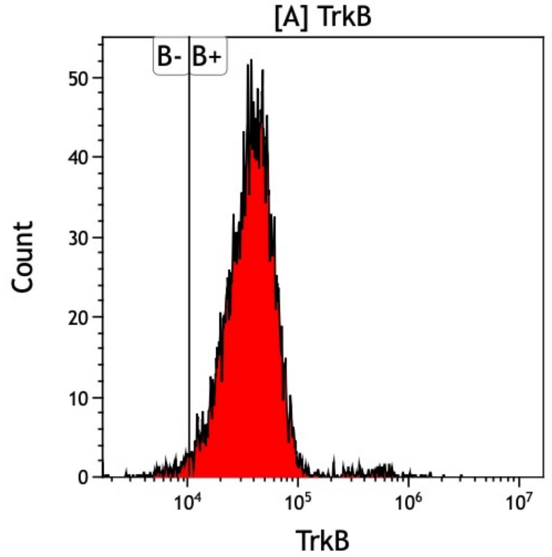

Gate	%Total	%Gated
All	57,86	100,00
B-	1,24	2,14
B+	56,62	97,86

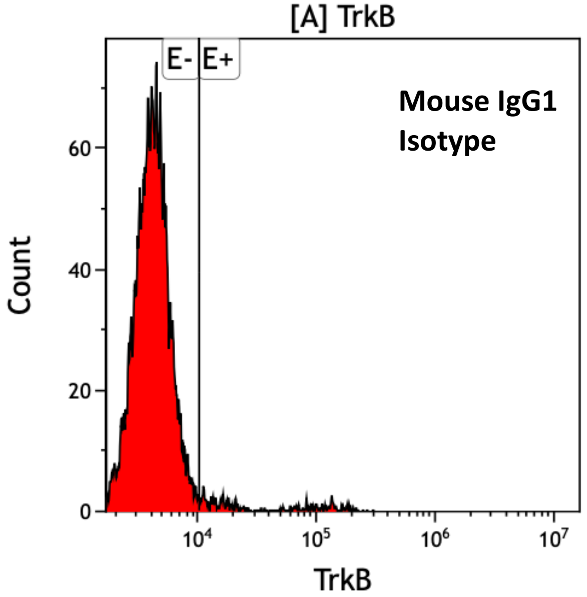

Patient 18

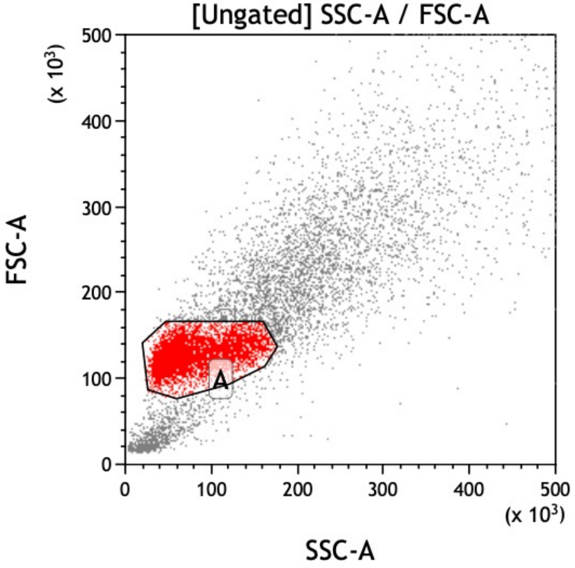

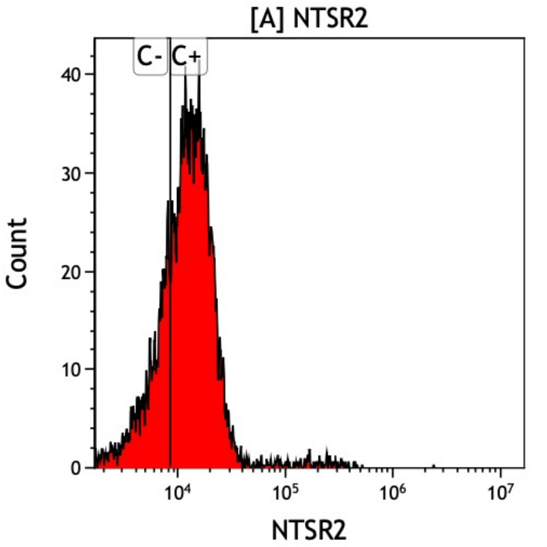

Gate	%Total	%Gated
All	50,89	100,00
C-	13,33	26,19
C+	37,56	73,81

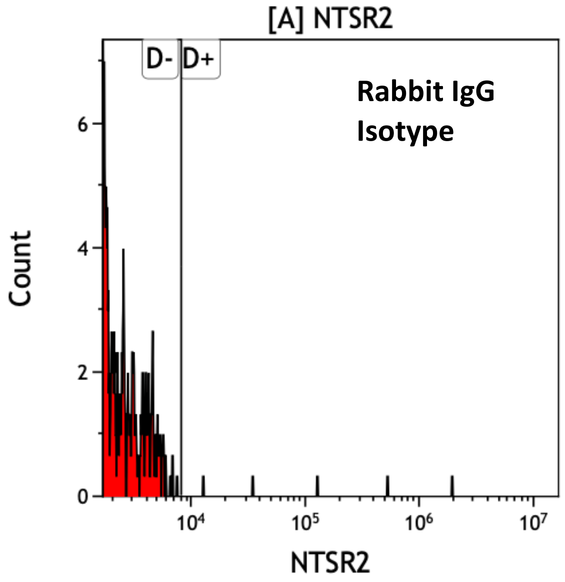

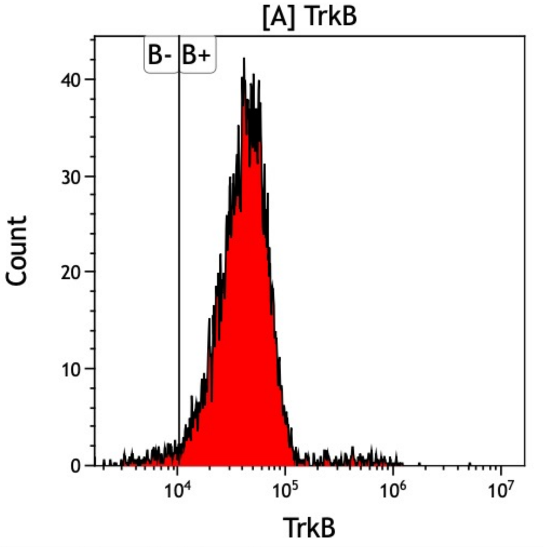

Gate	%Total	%Gated
All	50,89	100,00
B-	1,39	2,73
B+	49,50	97,27

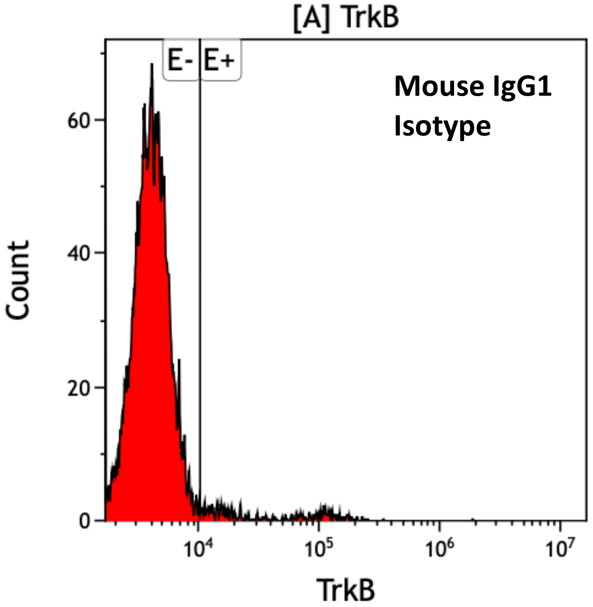

Patient 19

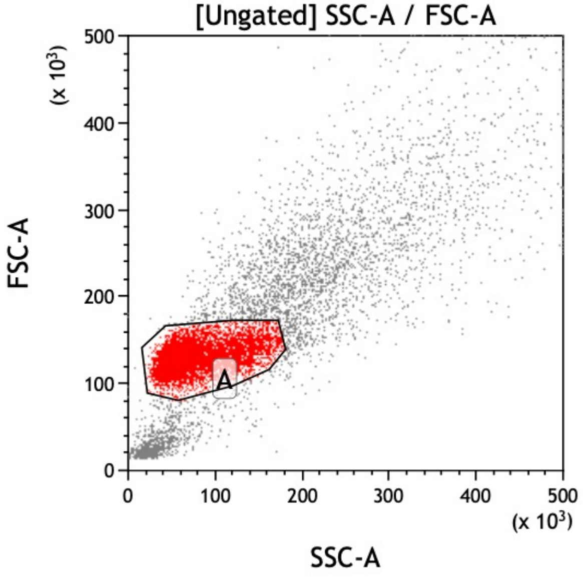

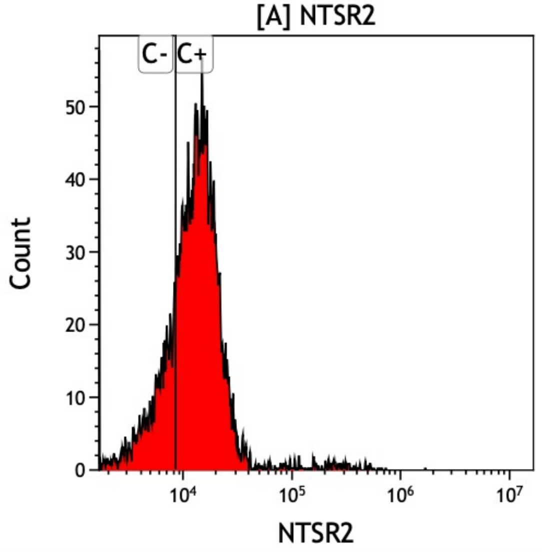

Gate	%Total	%Gated
All	60,12	100,00
C-	13,16	21,89
C+	46,96	78,11

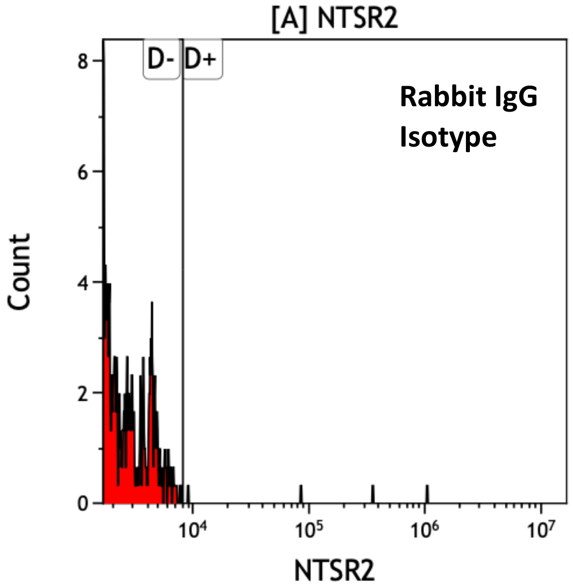

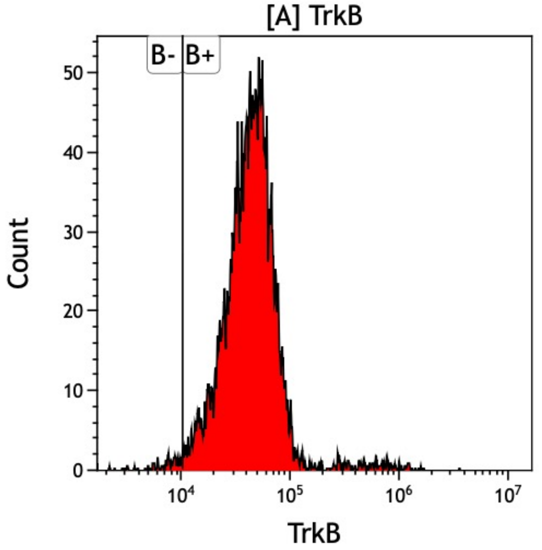

Gate	%Total	%Gated
All	60,12	100,00
B-	0,86	1,43
B+	59,26	98,57

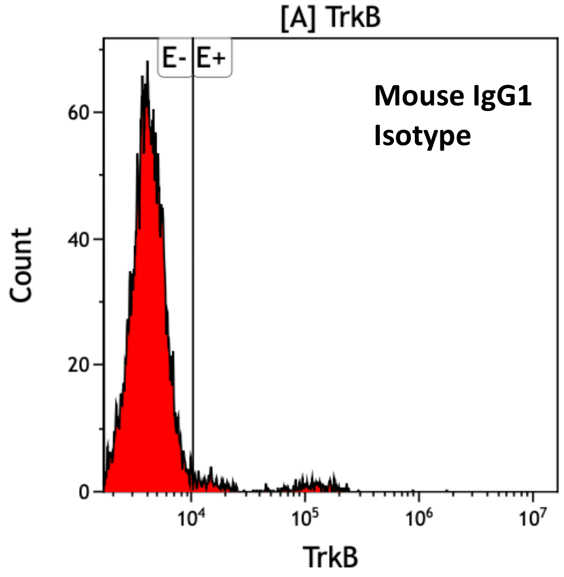

Patient 20

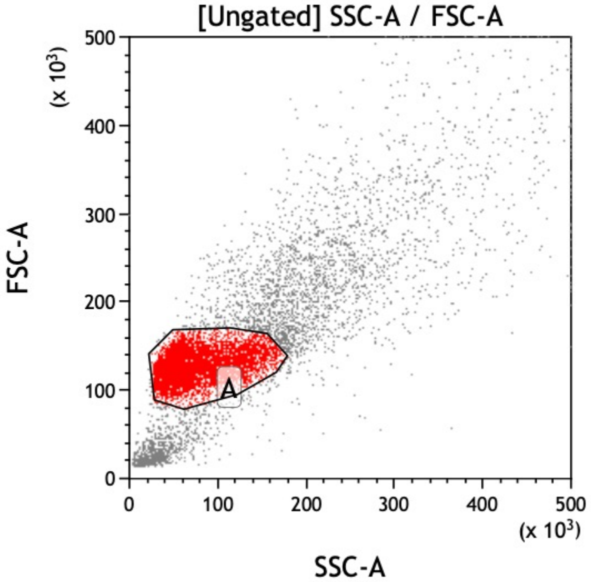

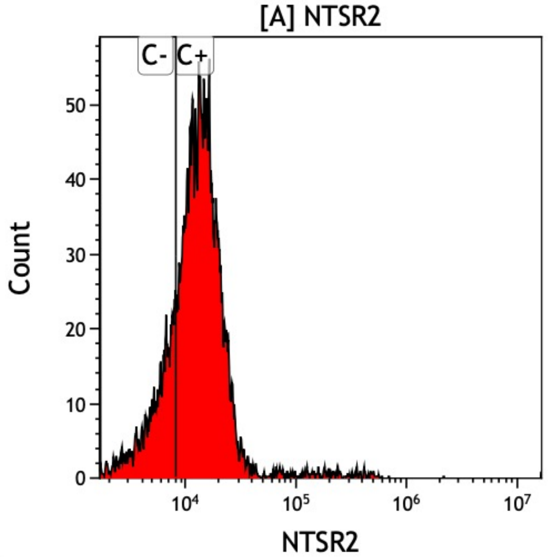

Gate	%Total	%Gated
All	63,58	100,00
C-	13,54	21,30
C+	50,04	78,70

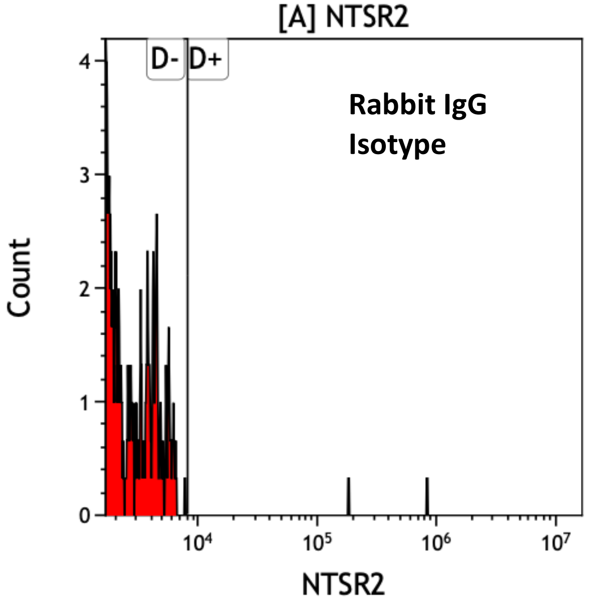

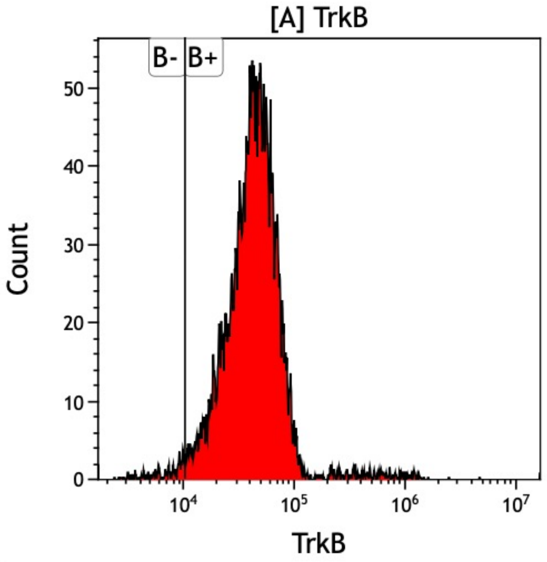

Gate	%Total	%Gated
All	63,58	100,00
B-	1,31	2,06
B+	62,27	97,94

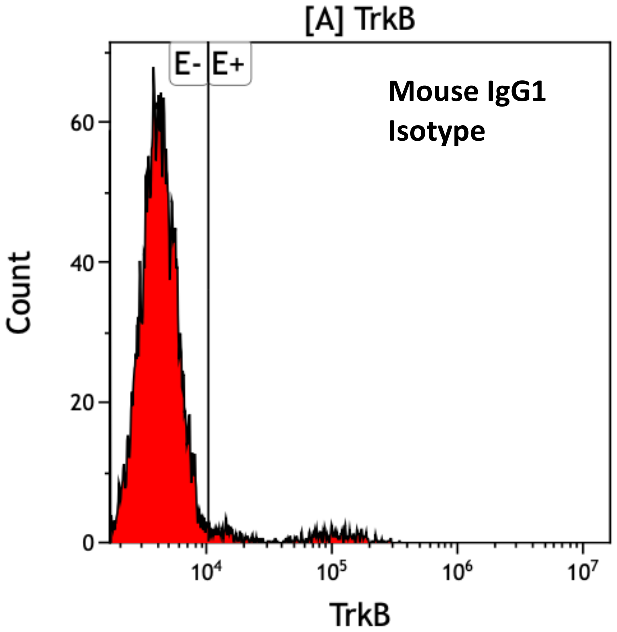

Patient 21

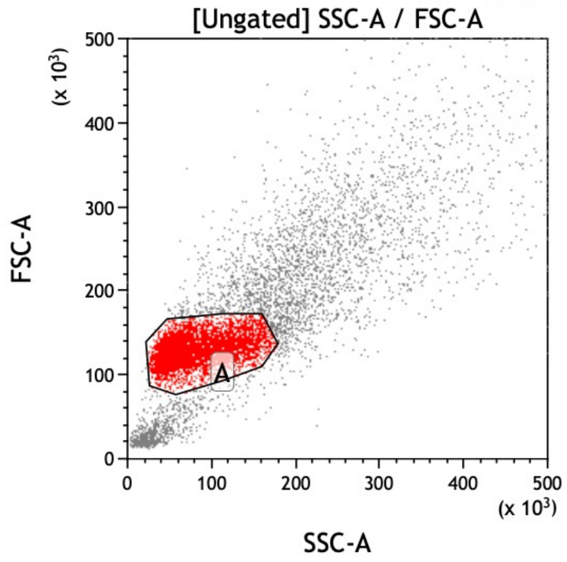

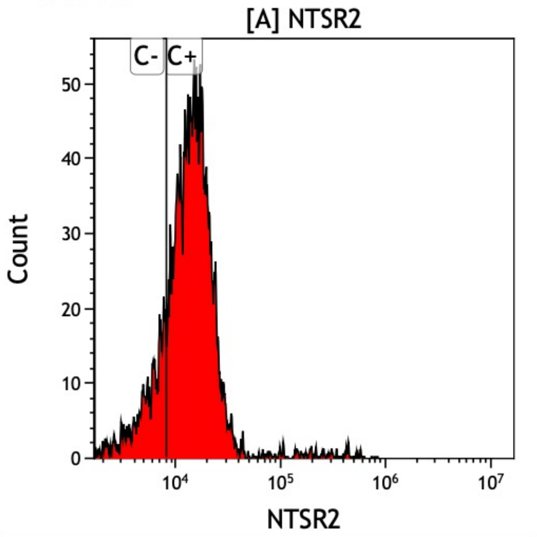

Gate	%Total	%Gated
All	60,42	100,00
C-	11,13	18,42
C+	49,29	81,58

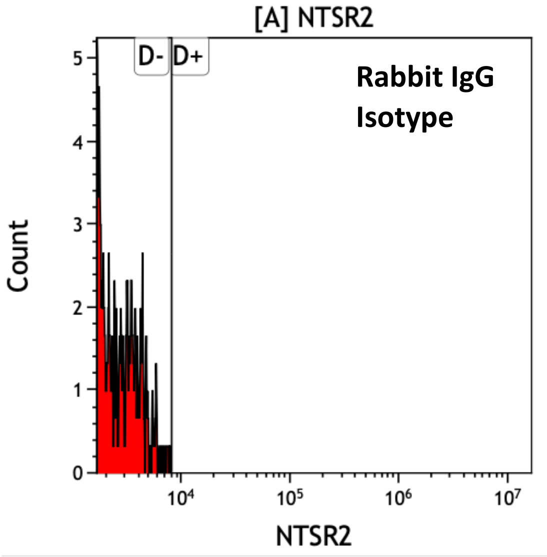

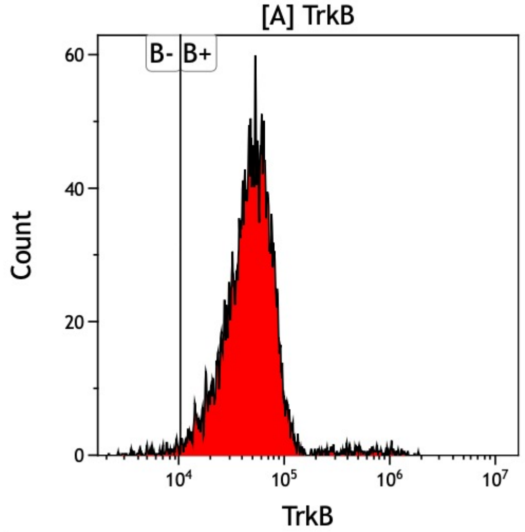

Gate	%Total	%Gated
All	60,42	100,00
B-	0,90	1,49
B+	59,52	98,51

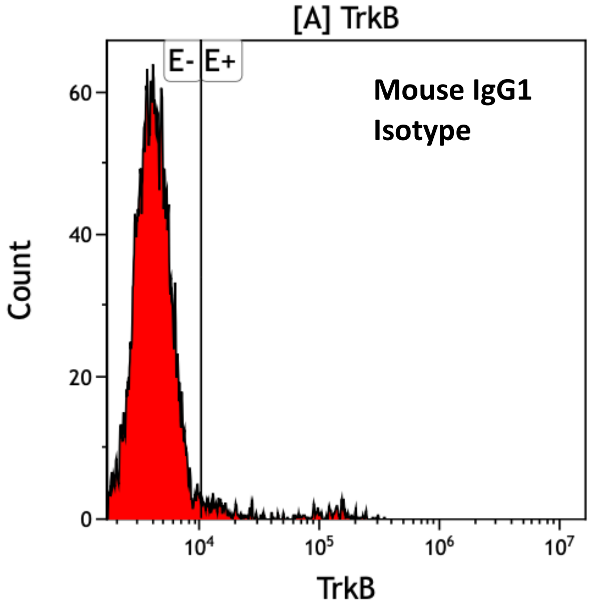

Patient 22

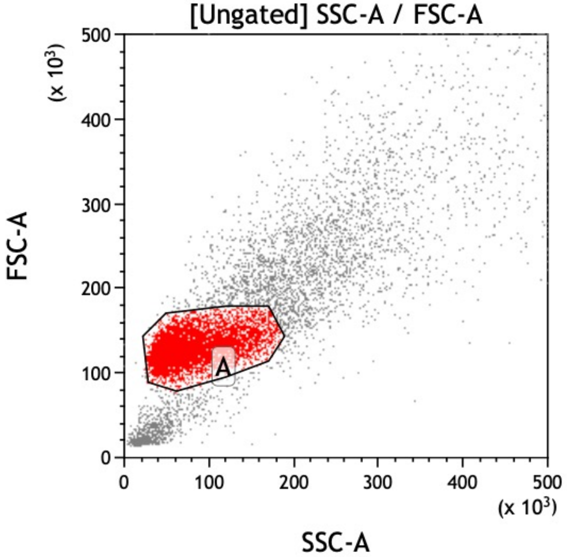

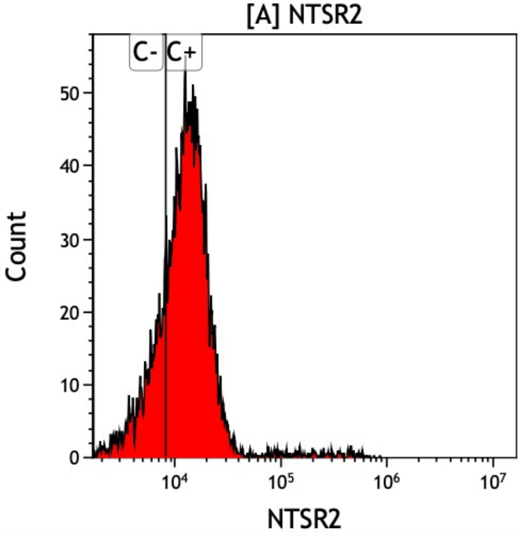

Gate	%Total	%Gated
All	61,27	100,00
C-	13,54	22,10
C+	47,73	77,90

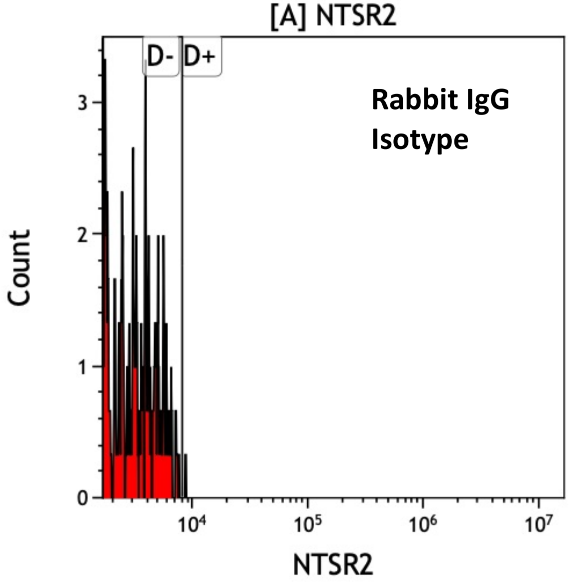

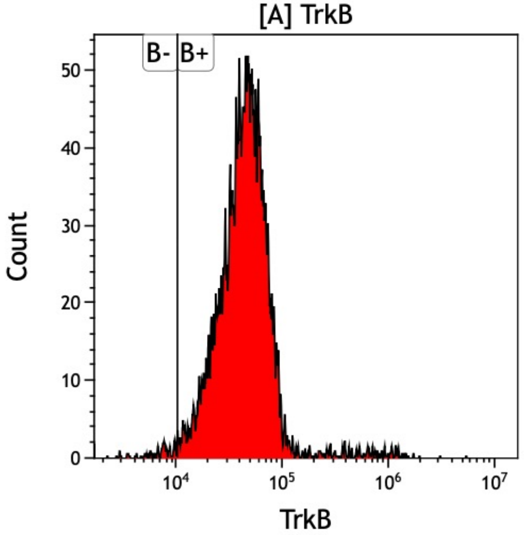

Gate	%Total	%Gated
All	61,27	100,00
B-	0,88	1,44
B+	60,39	98,56

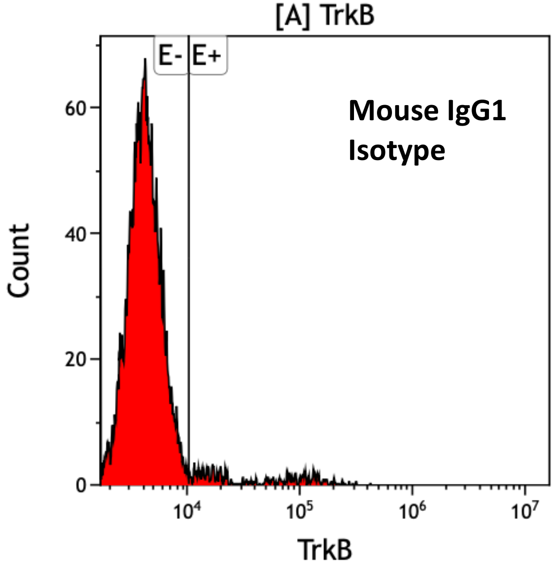

Patient 23

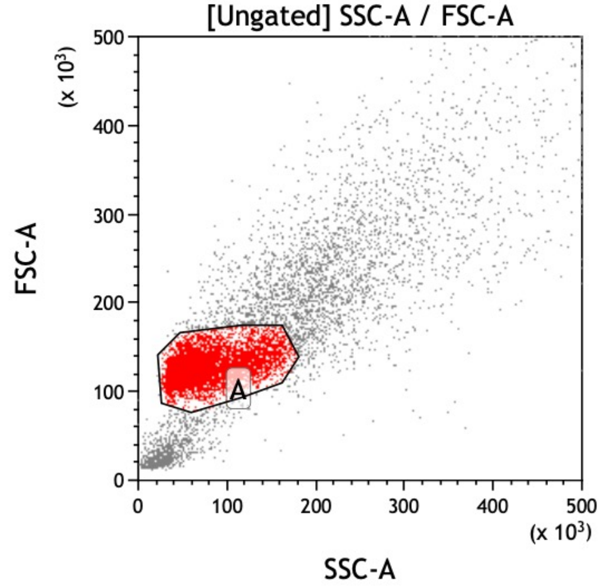

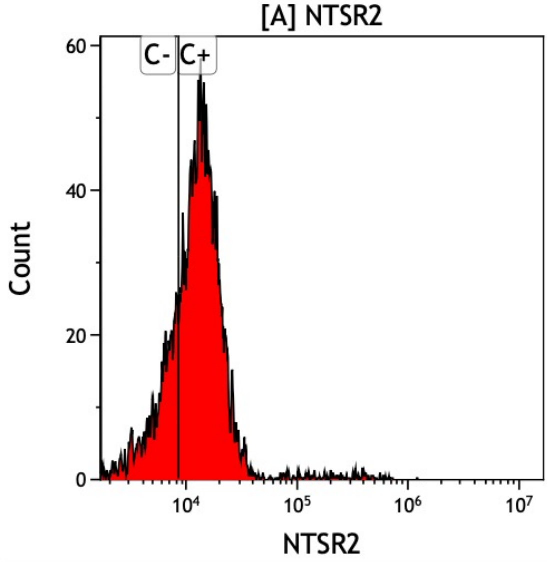

Gate	%Total	%Gated
All	61,10	100,00
C-	14,69	24,04
C+	46,41	75,96

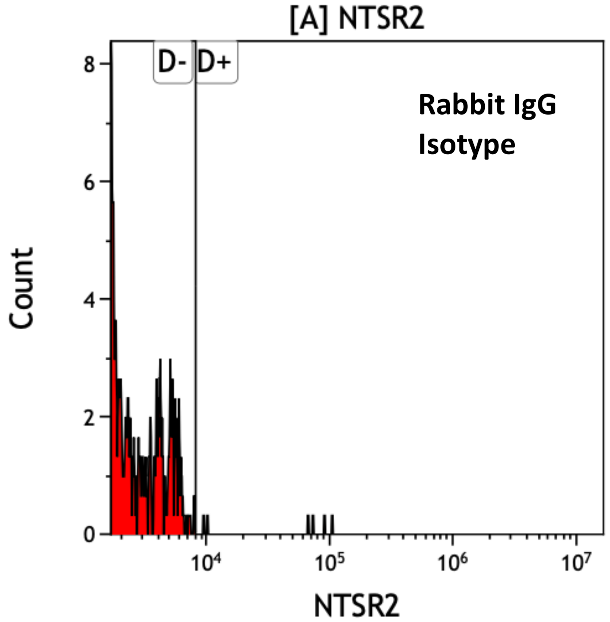

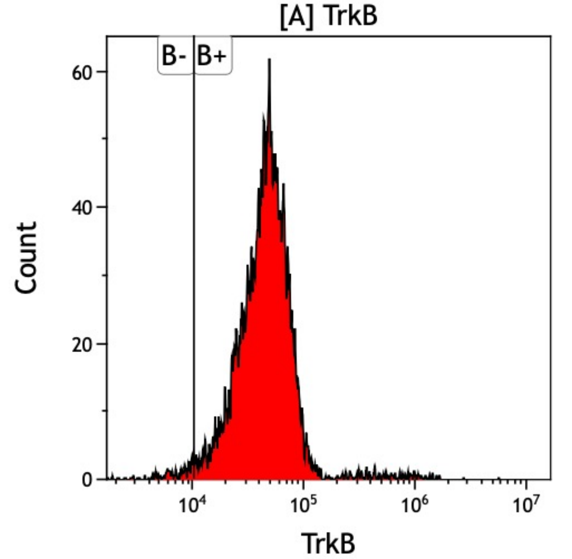

Gate	%Total	%Gated
All	61,10	100,00
B-	1,24	2,03
B+	59,86	97,97

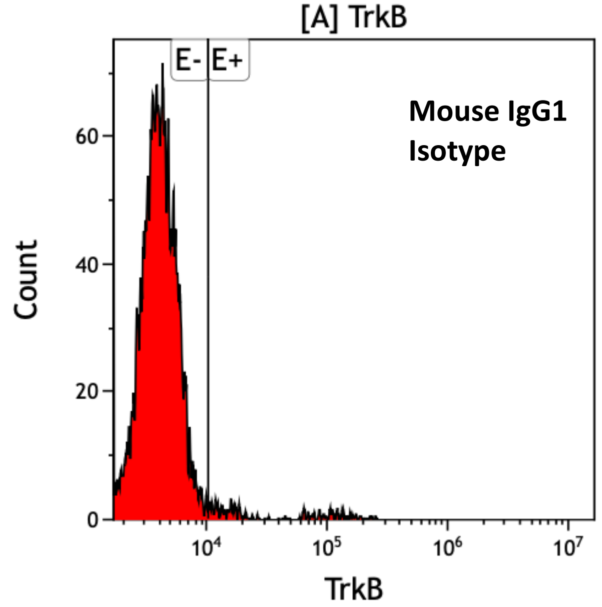

Patient 24

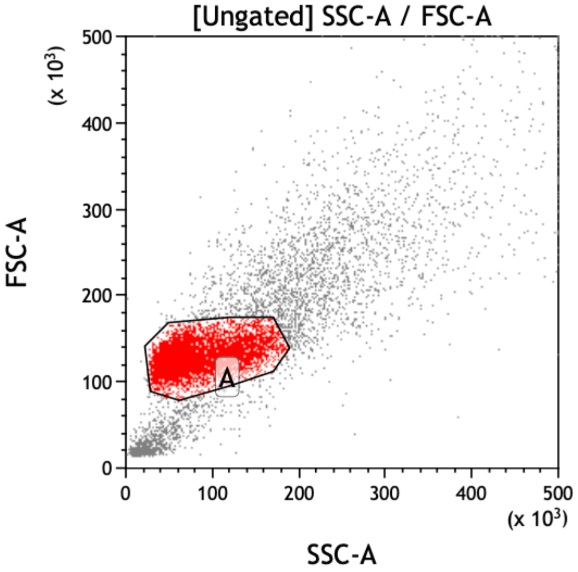

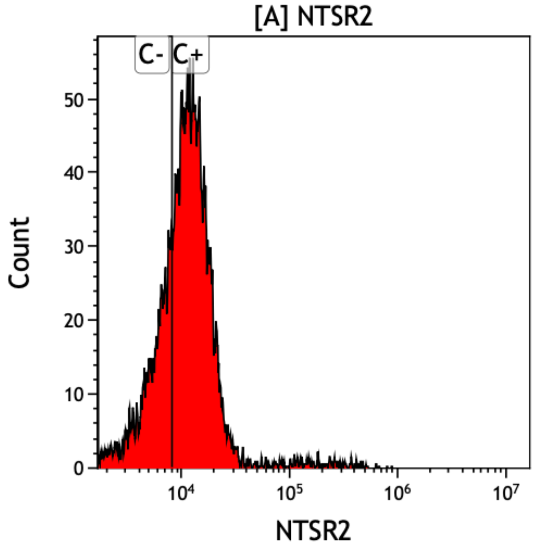

Gate	%Total	%Gated
All	65,68	100,00
C-	18,54	28,23
C+	47,14	71,77

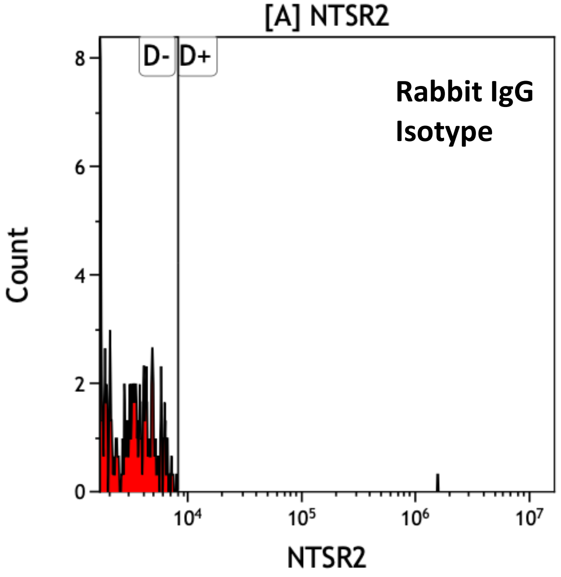

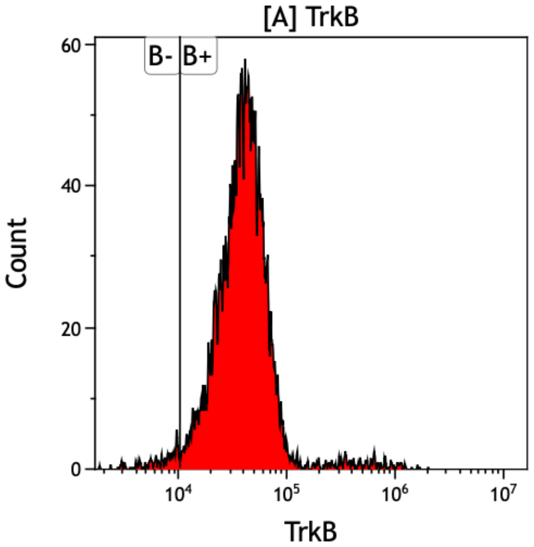

Gate	%Total	%Gated
All	65,68	100,00
B-	1,69	2,57
B+	63,99	97,43

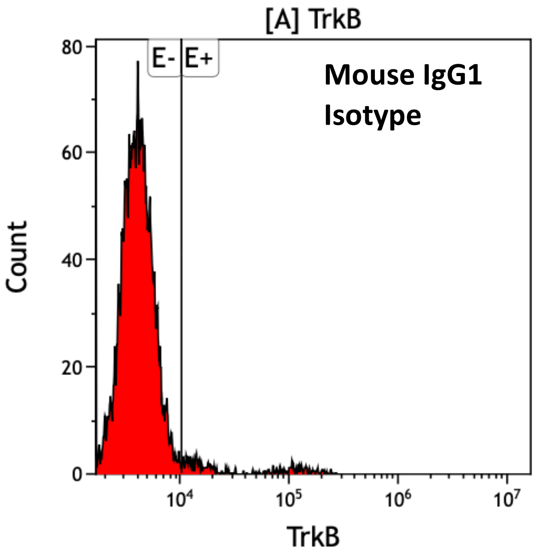

Patient 25

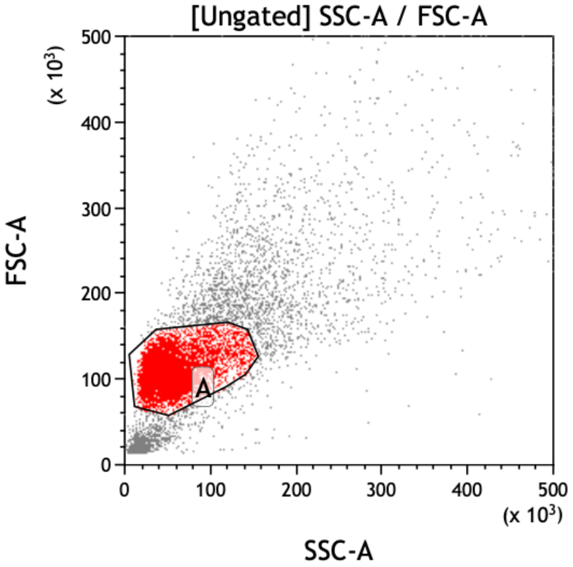

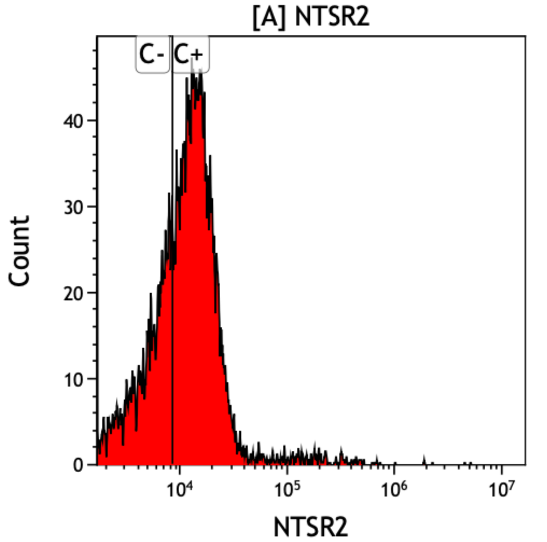

Gate	%Total	%Gated
All	68,65	100,00
C-	23,35	34,01
C+	45,30	65,99

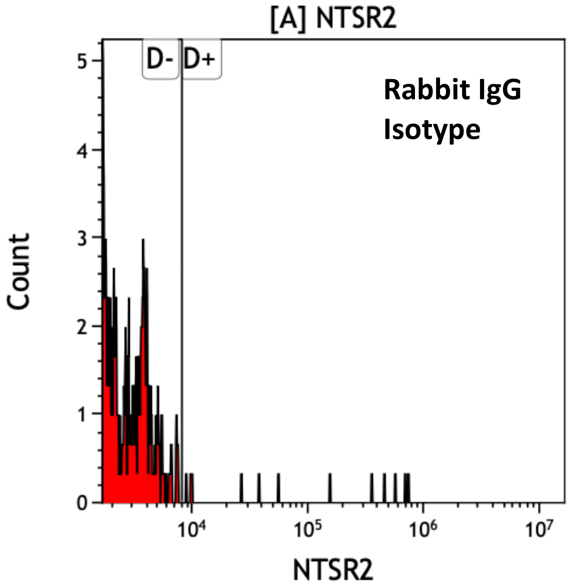

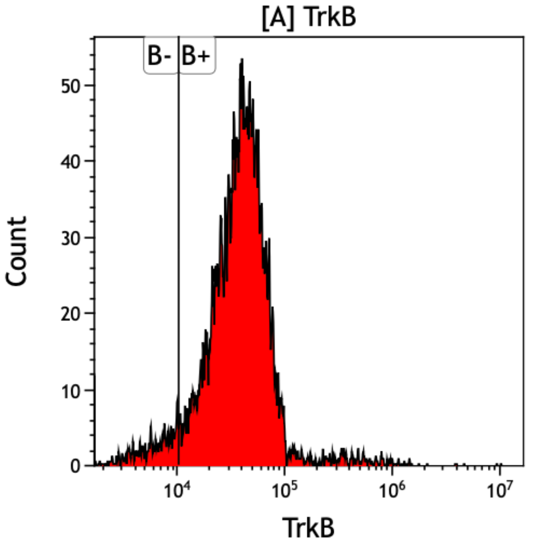

Gate	%Total	%Gated
All	68,65	100,00
B-	4,21	6,13
B+	64,44	93,87

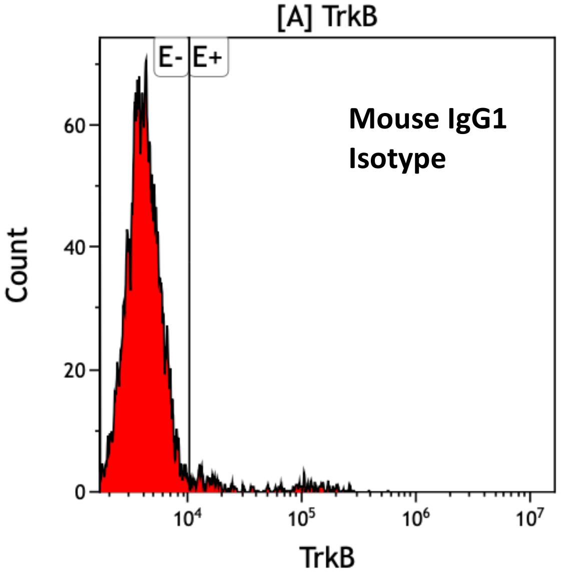

Patient 26

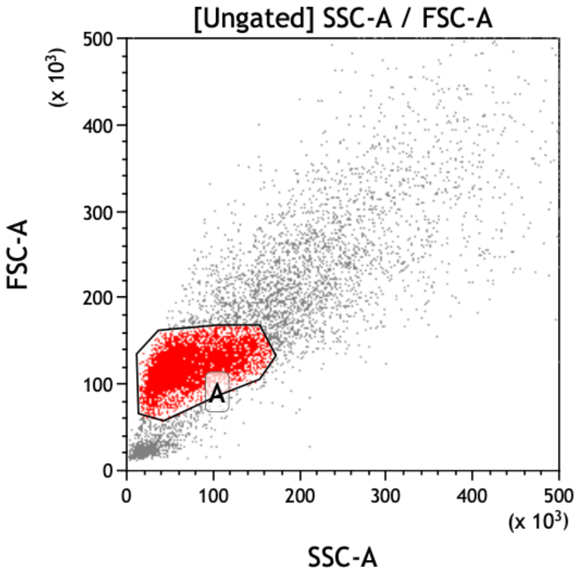

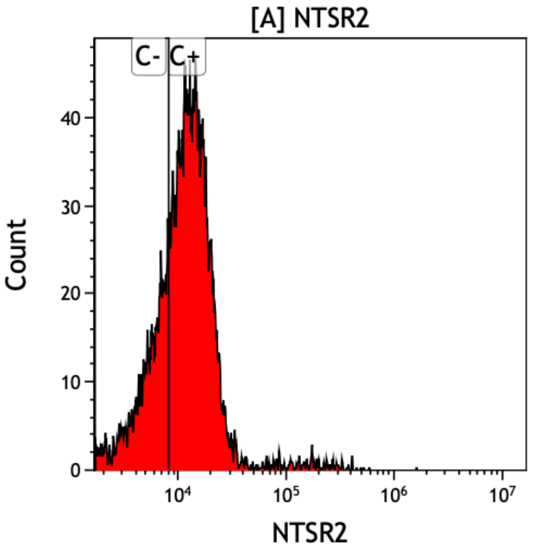

Gate	%Total	%Gated
All	61,11	100,00
C-	17,54	28,70
C+	43,57	71,30

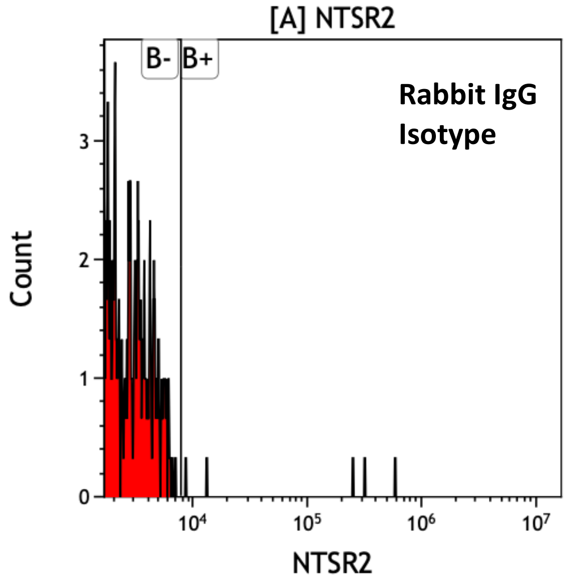

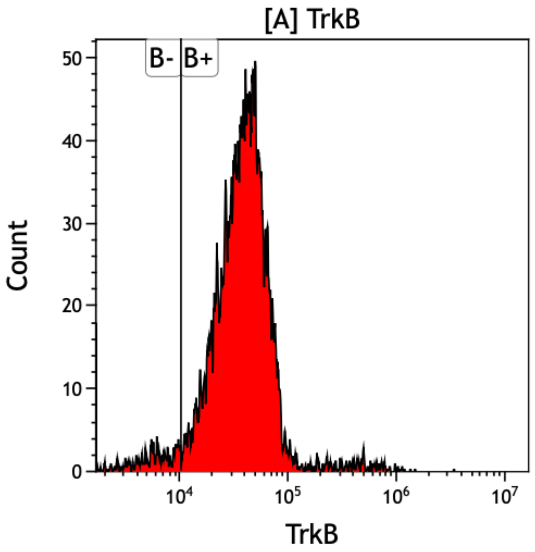

Gate	%Total	%Gated
All	61,11	100,00
B-	2,87	4,70
B+	58,24	95,30

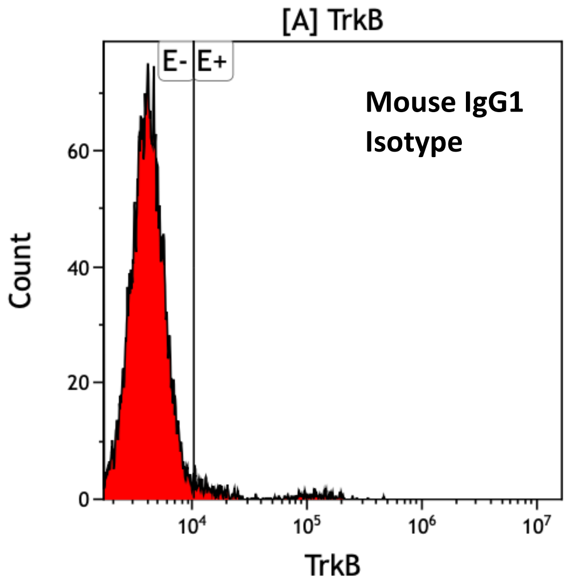

Patient 27

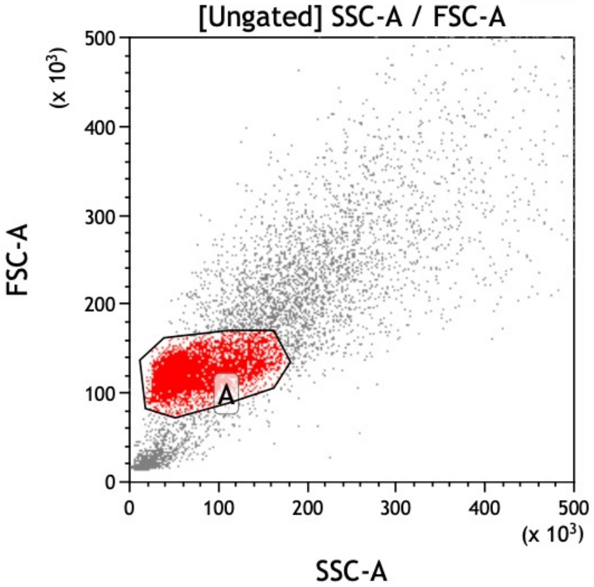

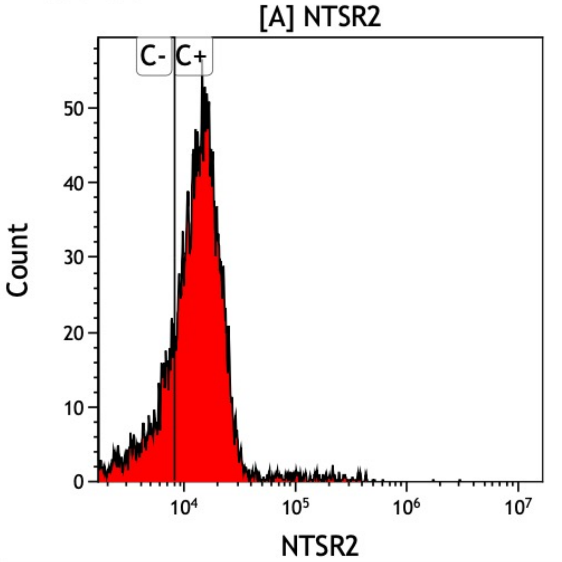

Gate	%Total	%Gated
All	61,25	100,00
C-	12,65	20,65
C+	48,60	79,35

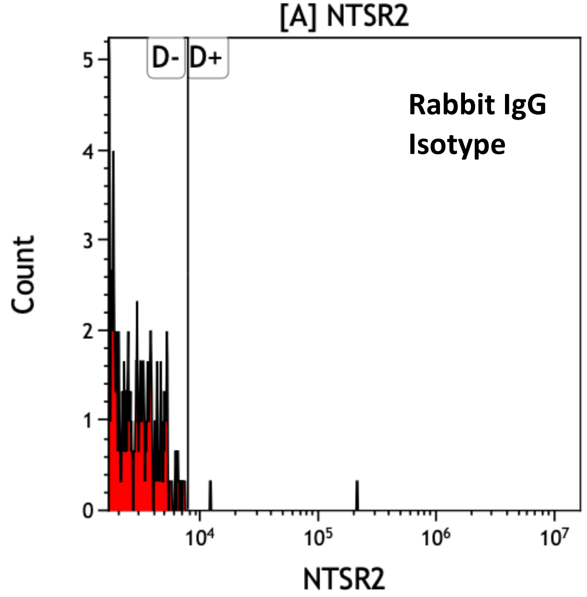

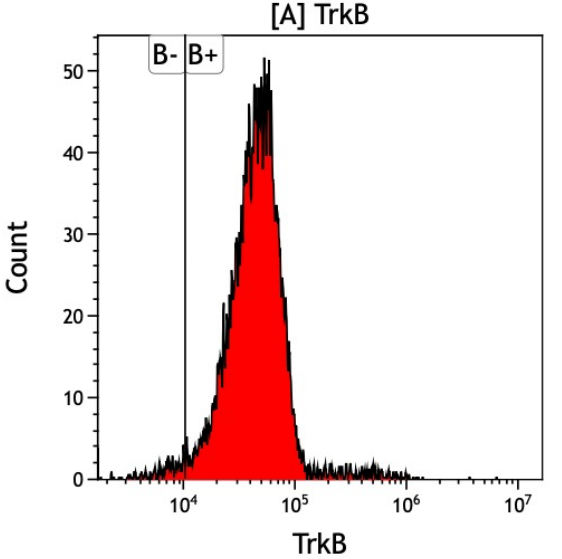

Gate	%Total	%Gated
All	61,25	100,00
B-	1,55	2,53
B+	59,70	97,47

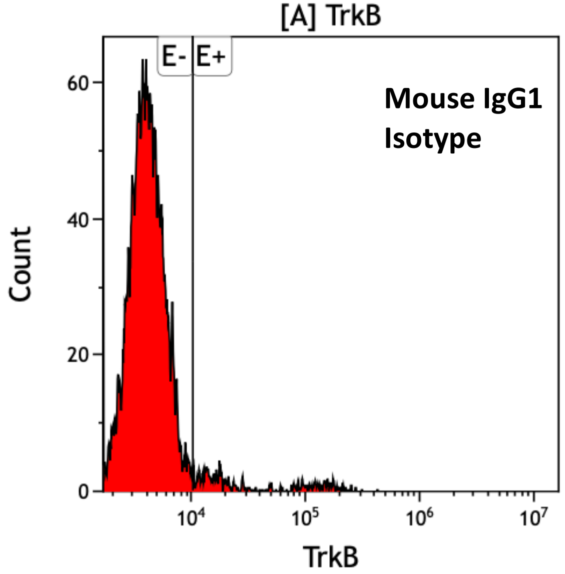

Patient 28

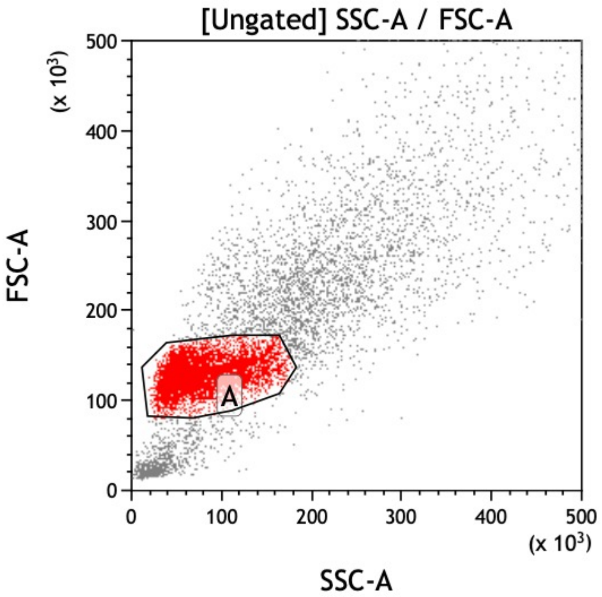

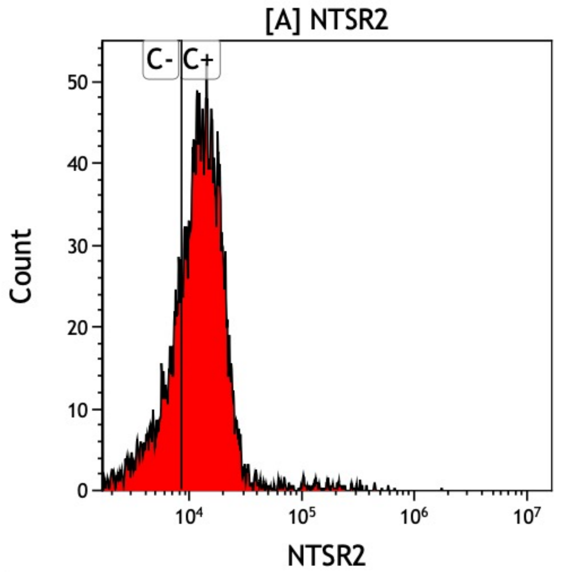

Gate	%Total	%Gated
All	58,84	100,00
C-	14,35	24,39
C+	44,49	75,61

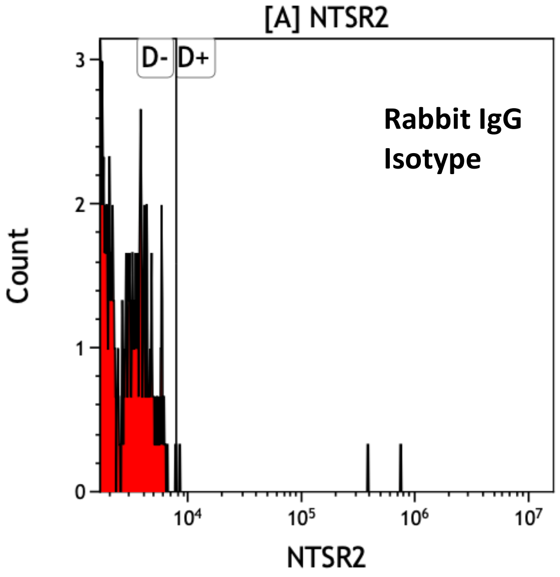

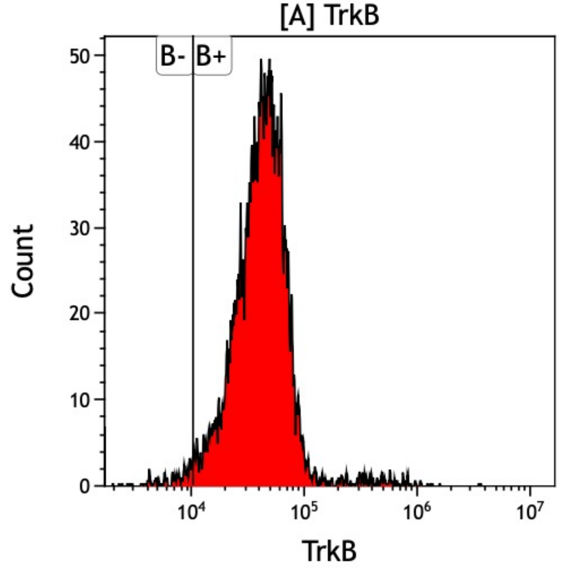

Gate	%Total	%Gated
All	58,84	100,00
B-	1,20	2,04
B+	57,64	97,96

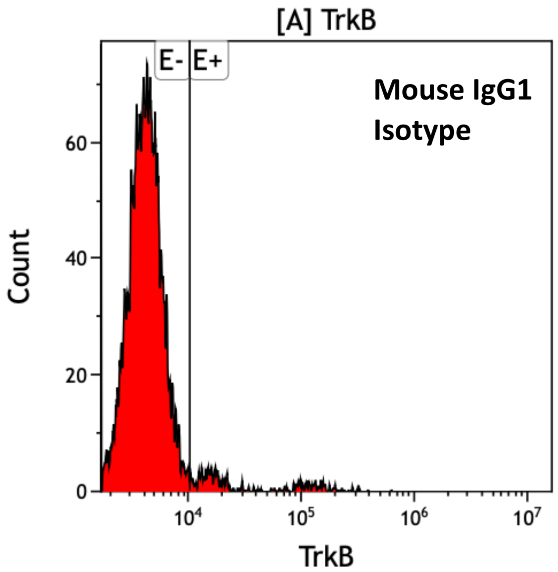

Patient 29

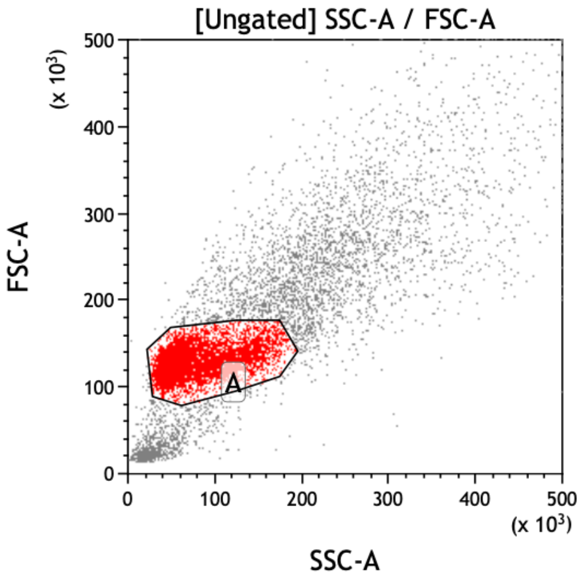

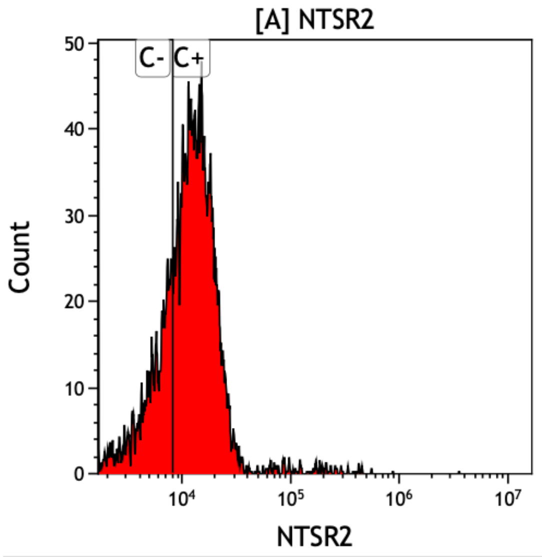

Gate	%Total	%Gated
All	57,37	100,00
C-	15,15	26,41
C+	42,22	73,59

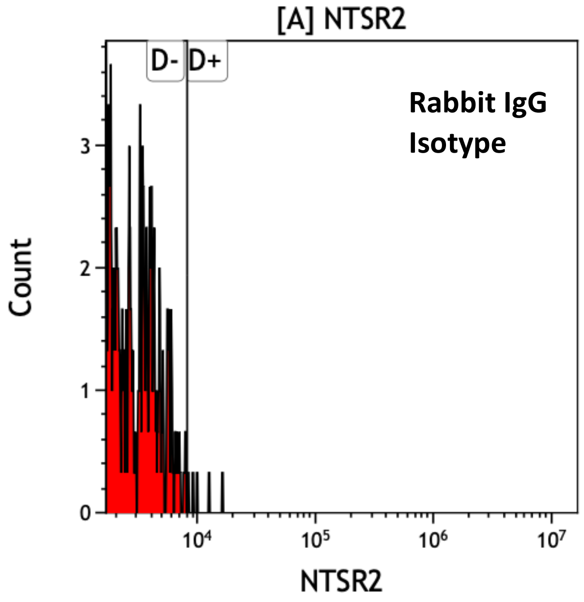

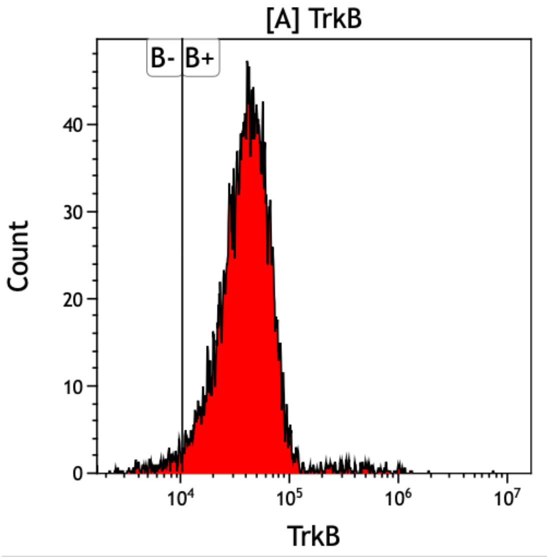

Gate	%Total	%Gated
All	57,37	100,00
B-	1,48	2,58
B+	55,89	97,42

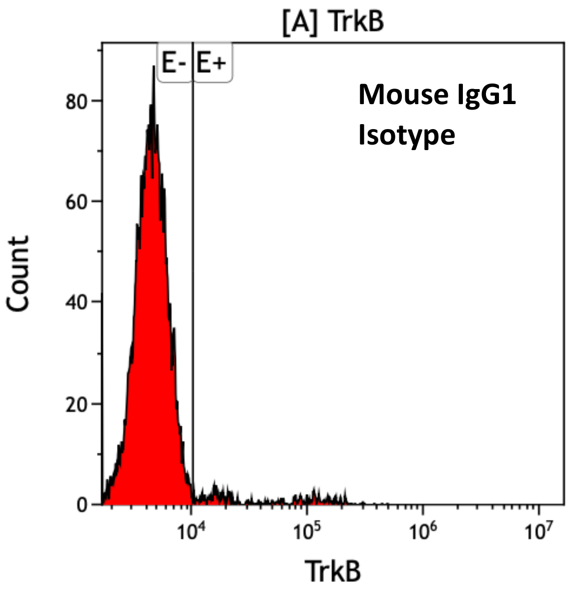

Patient 30

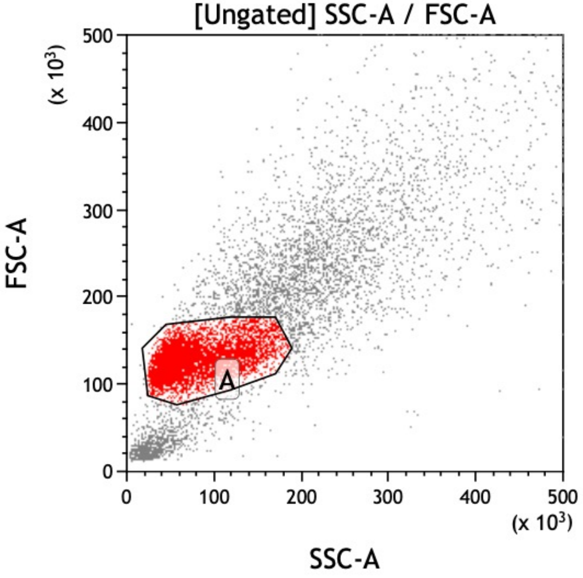

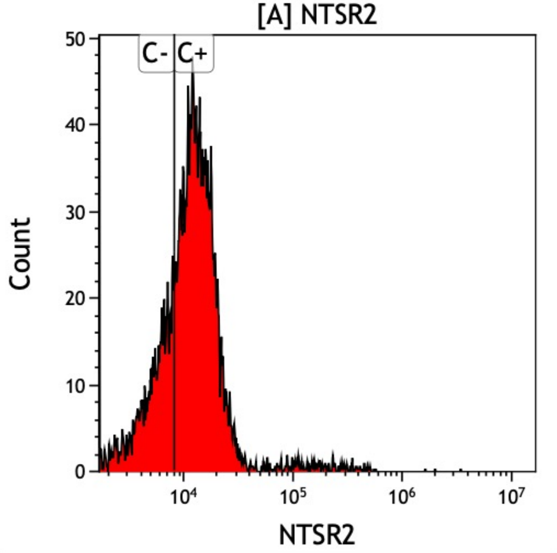

Gate	%Total	%Gated
All	54,41	100,00
C-	14,71	27,04
C+	39,70	72,96

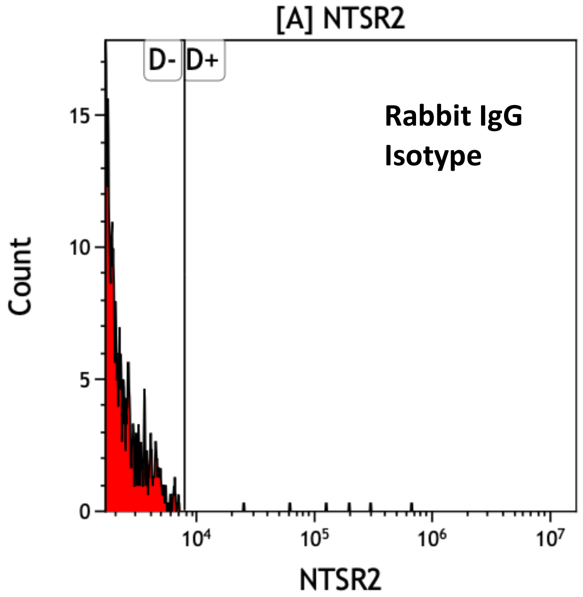

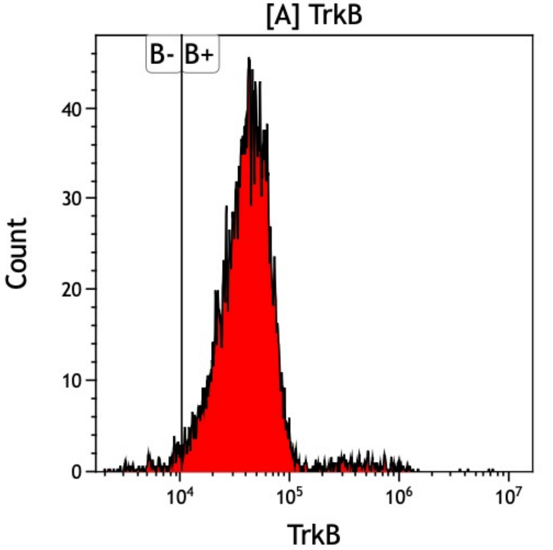

Gate	%Total	%Gated
All	54,41	100,00
B-	1,30	2,39
B+	53,11	97,61

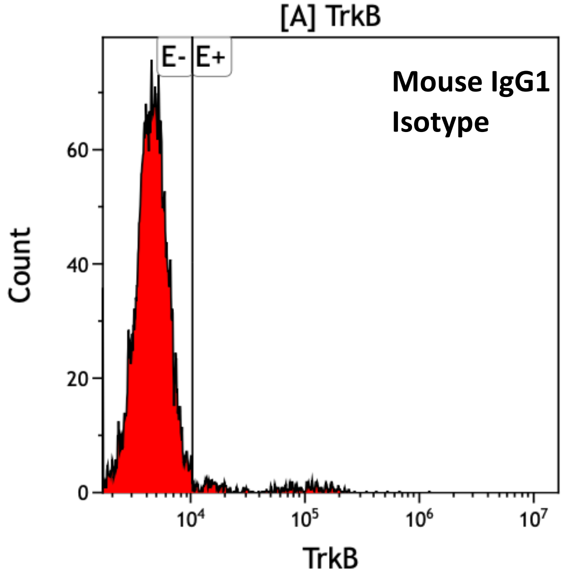

Supplement: Supplementary file 1 — Supplementary Information 1. [file 41598_2024_56663_MOESM1_ESM.pdf]
