# Supplementary material for: Targeting the NTSR2/TrkB oncogenic pathway in chronic lymphocytic leukemia
Source: Sci Rep. 2024 Mar 13;14:6084. doi: 10.1038/s41598-024-56663-5 (PMC10937676; doi:10.1038/s41598-024-56663-5)
Supplement: Supplementary file 2 — Supplementary Information 2. [file 41598_2024_56663_MOESM2_ESM.pdf]

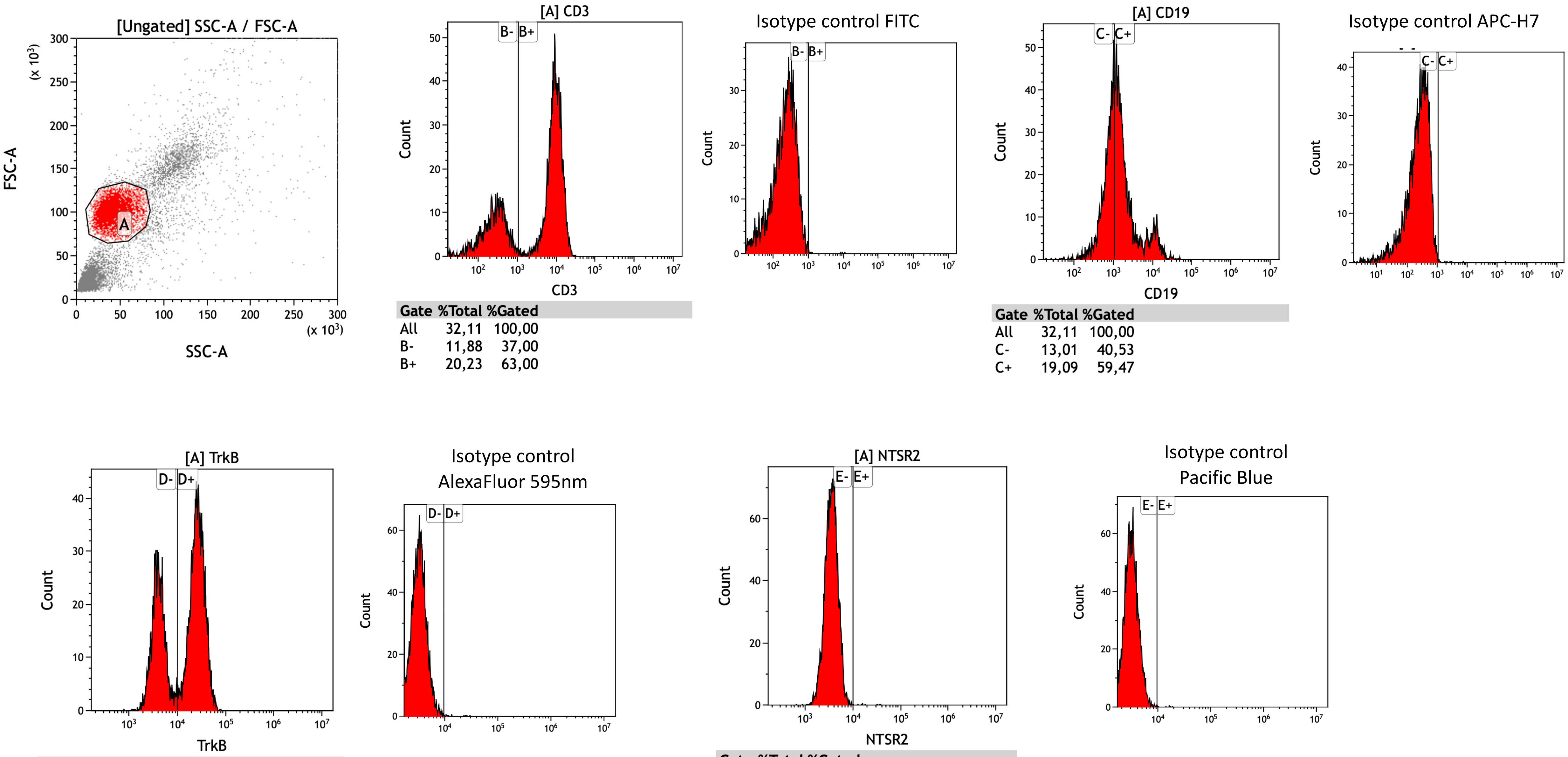


| Gate | %Total | %Gated |
|---|---|---|
| All | 32,11 | 100,00 |
| B- | 11,88 | 37,00 |
| B+ | 20,23 | 63,00 |

| Gate | %Total | %Gated |
|---|---|---|
| All | 32,11 | 100,00 |
| C- | 13,01 | 40,53 |
| C+ | 19,09 | 59,47 |

| Gate | %Total | %Gated |
|---|---|---|
| All | 32,11 | 100,00 |
| D- | 12,33 | 38,41 |
| D+ | 19,77 | 61,59 |

| Gate | %Total | %Gated |
|---|---|---|
| All | 32,11 | 100,00 |
| E- | 32,05 | 99,81 |
| E+ | 0,06 | 0,19 |

**HEALTHY DONOR 1**

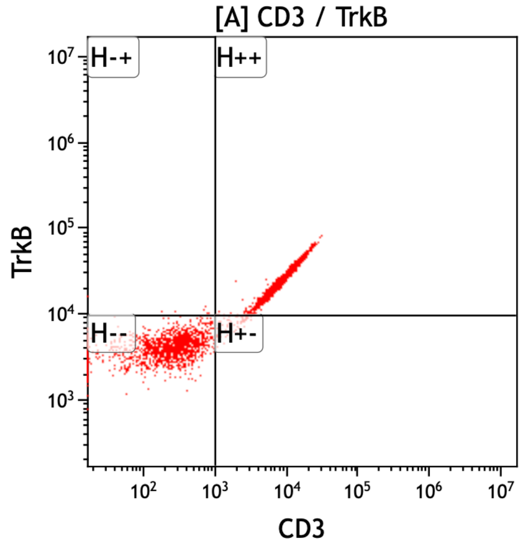


| Gate | %Gated |
| --- | --- |
| All | 100,00 |
| H-- | 36,77 |
| H-+ | 0,15 |
| H+- | 1,52 |
| H++ | 61,57 |

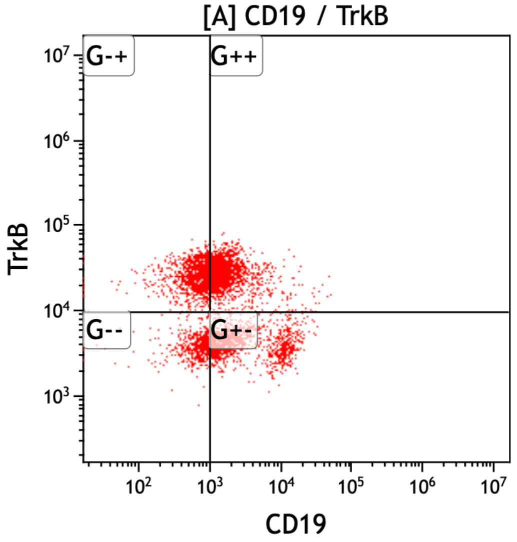


| Gate | %Gated |
| --- | --- |
| All | 100,00 |
| G-- | 11,63 |
| G-+ | 24,63 |
| G+- | 26,66 |
| G++ | 37,08 |

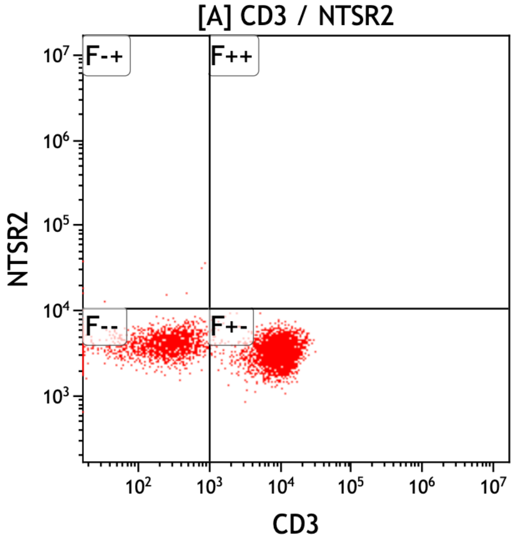


| Gate | %Gated |
| --- | --- |
| All | 100,00 |
| F-- | 36,71 |
| F-+ | 0,17 |
| F+- | 63,12 |
| F++ | 0,00 |

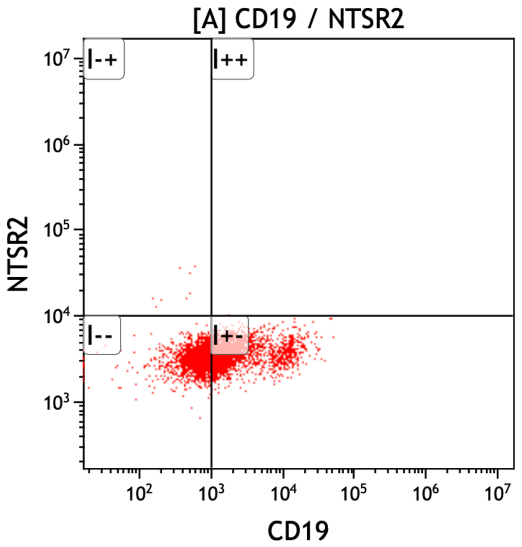


| Gate | %Gated |
| --- | --- |
| All | 100,00 |
| I-- | 36,40 |
| I-+ | 0,17 |
| I+- | 63,41 |
| I++ | 0,02 |

**HEALTHY DONOR 1**

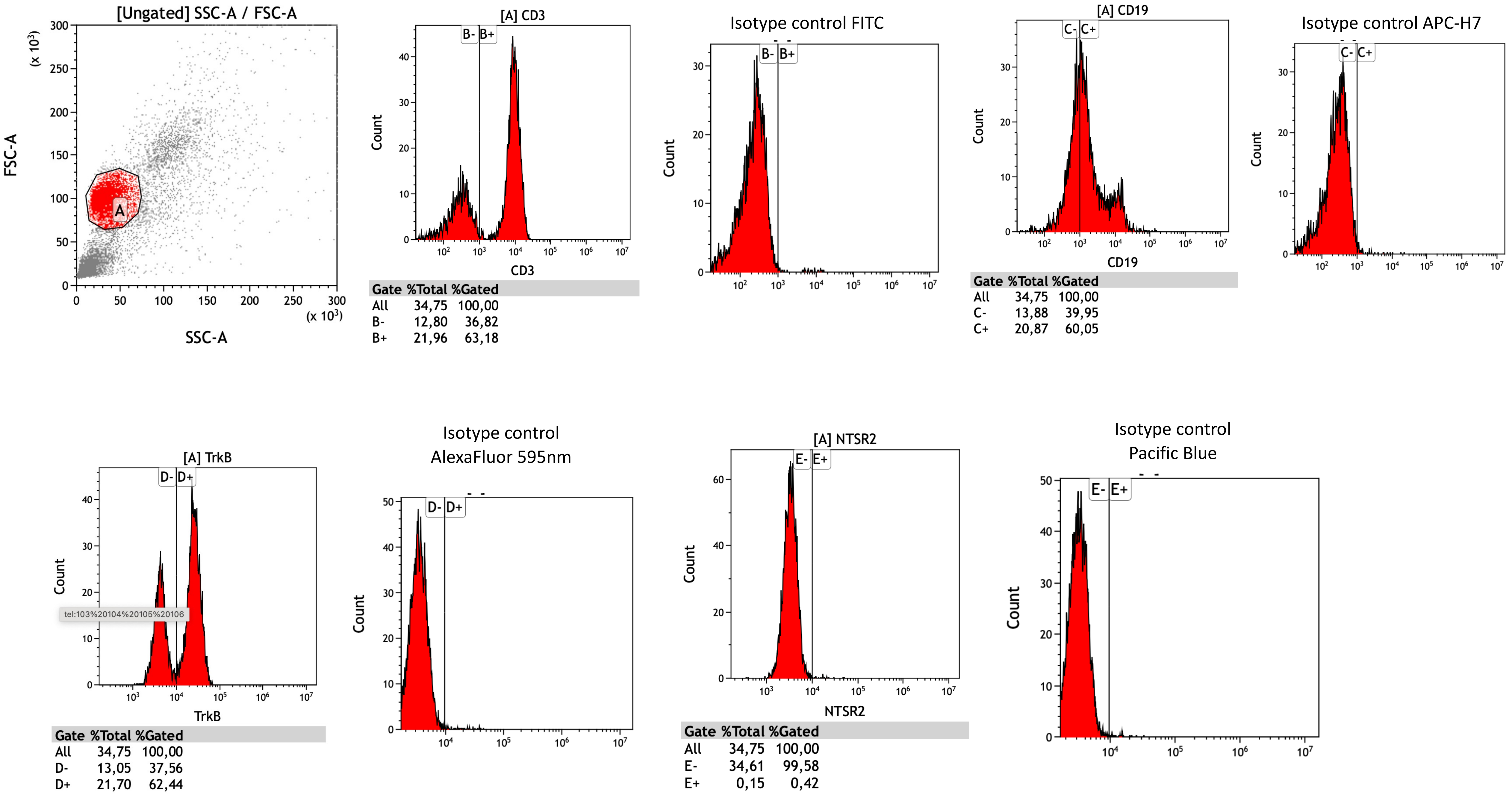


**HEALTHY DONOR 2**

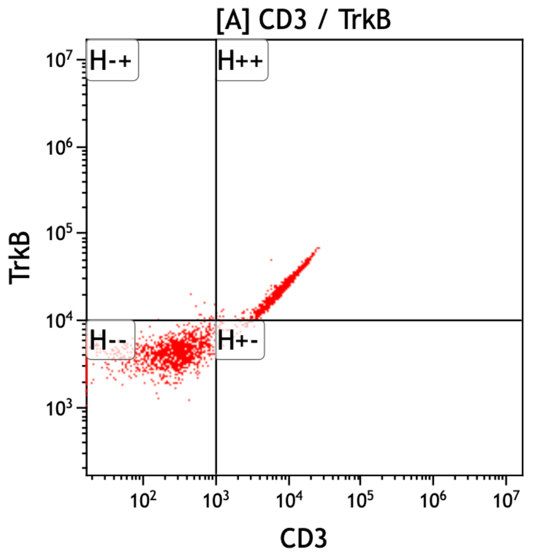


| Gate | %Gated |
|---|---|
| All | 100,00 |
| H-- | 36,52 |
| H-+ | 0,32 |
| H+- | 0,90 |
| H++ | 62,25 |

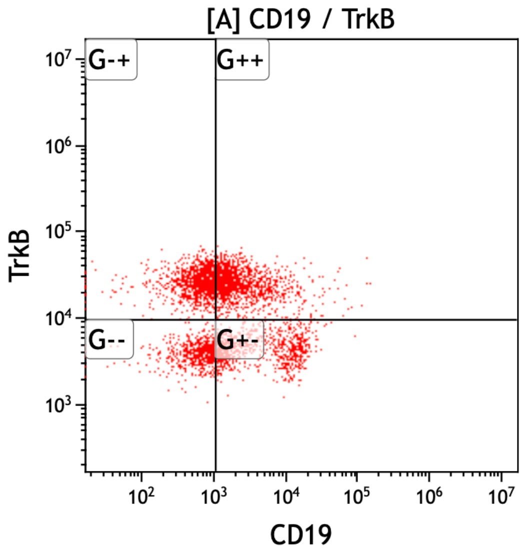


| Gate | %Gated |
|---|---|
| All | 100,00 |
| G-- | 15,21 |
| G-+ | 27,17 |
| G+- | 22,00 |
| G++ | 35,61 |

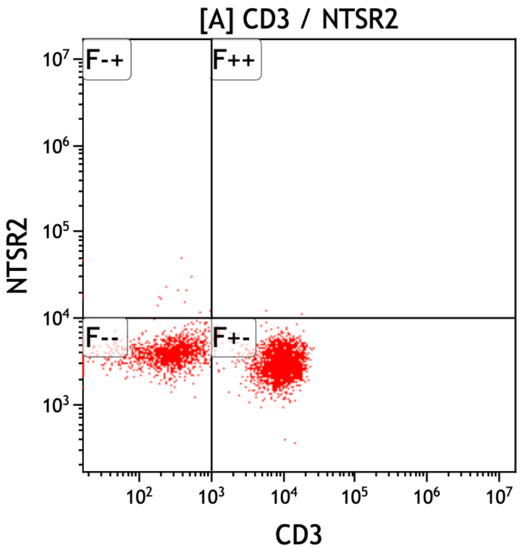


| Gate | %Gated |
|---|---|
| All | 100,00 |
| F-- | 36,49 |
| F-+ | 0,32 |
| F+- | 63,09 |
| F++ | 0,09 |

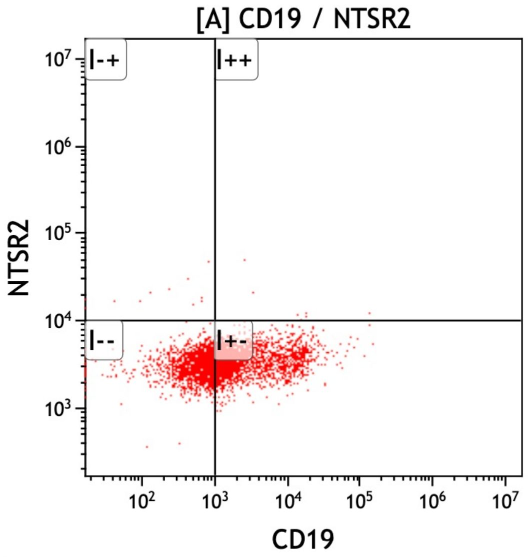


| Gate | %Gated |
|---|---|
| All | 100,00 |
| I-- | 39,60 |
| I-+ | 0,26 |
| I+- | 59,98 |
| I++ | 0,16 |

**HEALTHY DONOR 2**

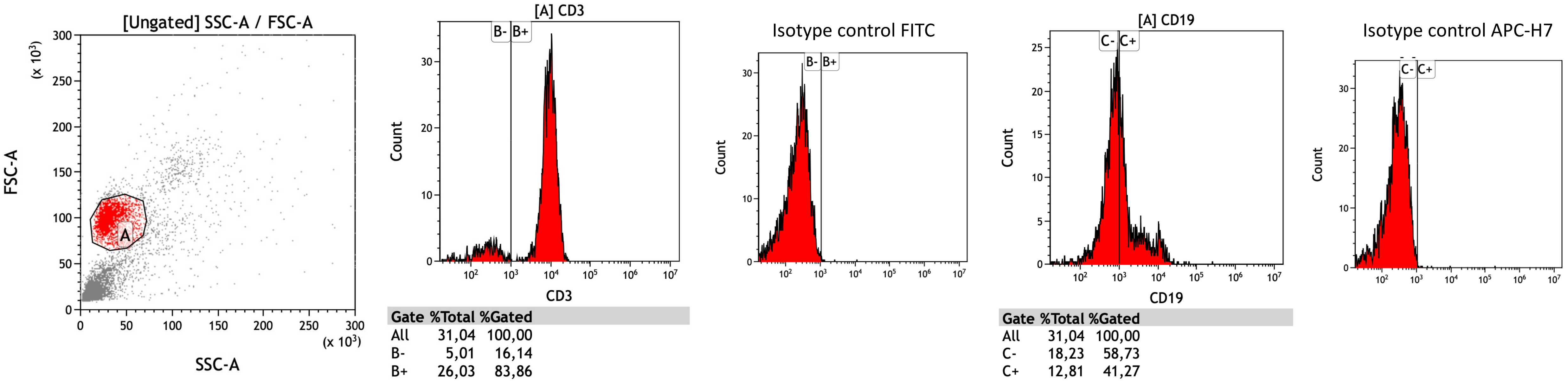


| Gate | %Total | %Gated |
|---|---|---|
| All | 31,04 | 100,00 |
| B- | 5,01 | 16,14 |
| B+ | 26,03 | 83,86 |

| Gate | %Total | %Gated |
|---|---|---|
| All | 31,04 | 100,00 |
| C- | 18,23 | 58,73 |
| C+ | 12,81 | 41,27 |

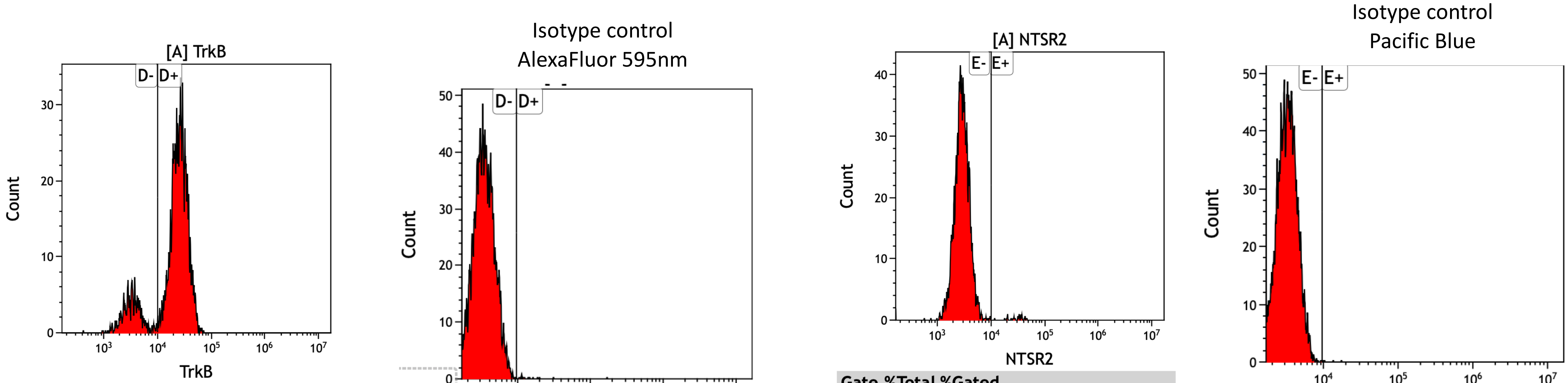


| Gate | %Total | %Gated |
|---|---|---|
| All | 31,04 | 100,00 |
| D- | 5,27 | 16,98 |
| D+ | 25,77 | 83,02 |

| Gate | %Total | %Gated |
|---|---|---|
| All | 31,04 | 100,00 |
| E- | 30,69 | 98,88 |
| E+ | 0,35 | 1,12 |

**HEALTHY DONOR 3**

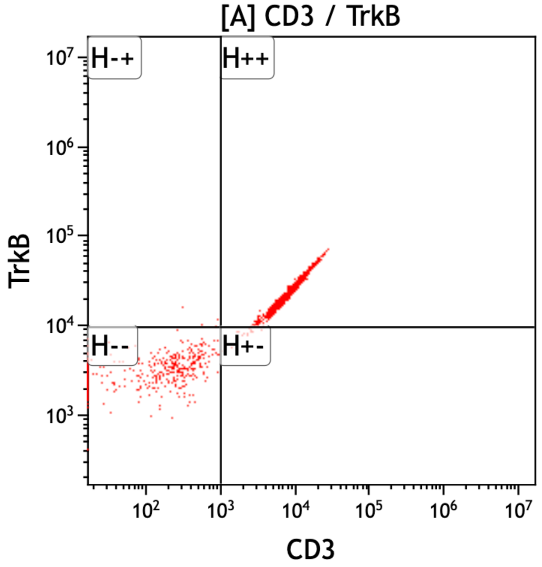


| Gate | %Gated |
|---|---|
| All | 100,00 |
| H-- | 16,02 |
| H-+ | 0,12 |
| H+- | 0,76 |
| H++ | 83,10 |

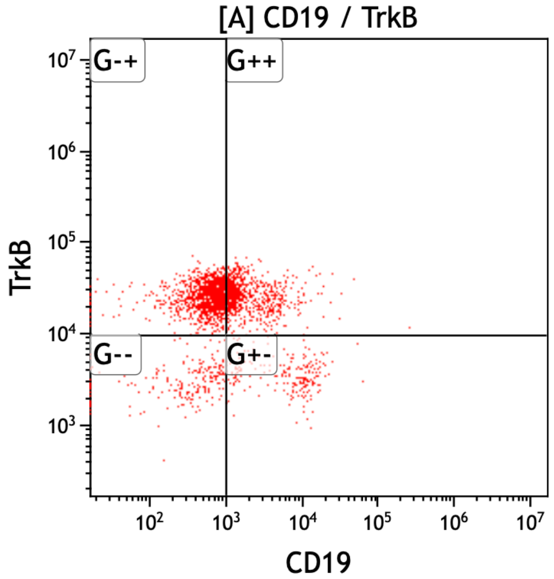


| Gate | %Gated |
|---|---|
| All | 100,00 |
| G-- | 8,27 |
| G-+ | 50,30 |
| G+- | 8,51 |
| G++ | 32,92 |

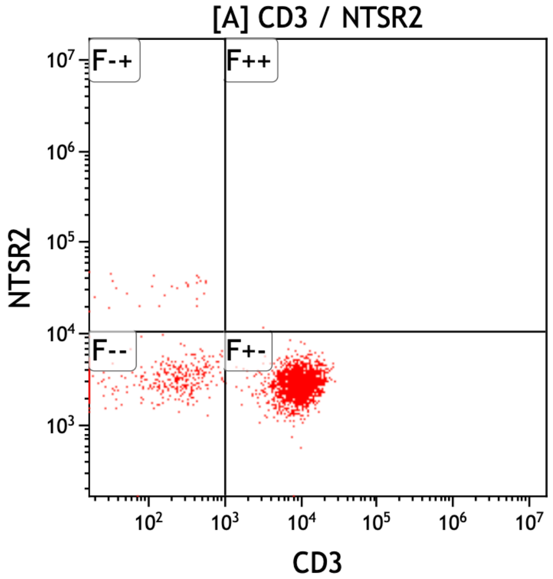


| Gate | %Gated |
|---|---|
| All | 100,00 |
| F-- | 15,05 |
| F-+ | 1,08 |
| F+- | 83,82 |
| F++ | 0,04 |

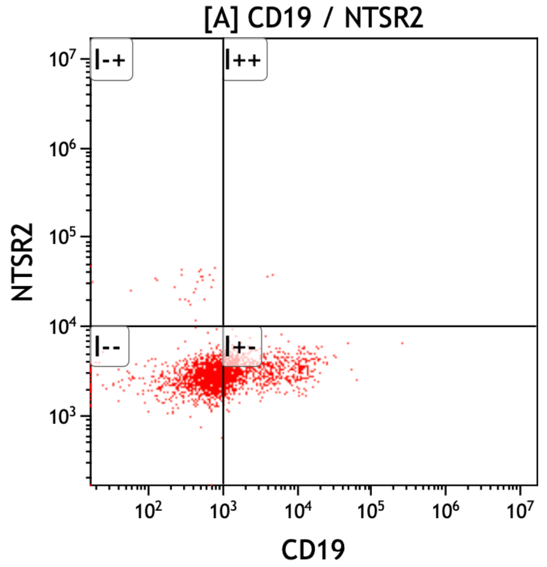


| Gate | %Gated |
|---|---|
| All | 100,00 |
| I-- | 57,89 |
| I-+ | 1,04 |
| I+- | 40,99 |
| I++ | 0,08 |

**HEALTHY DONOR 3**

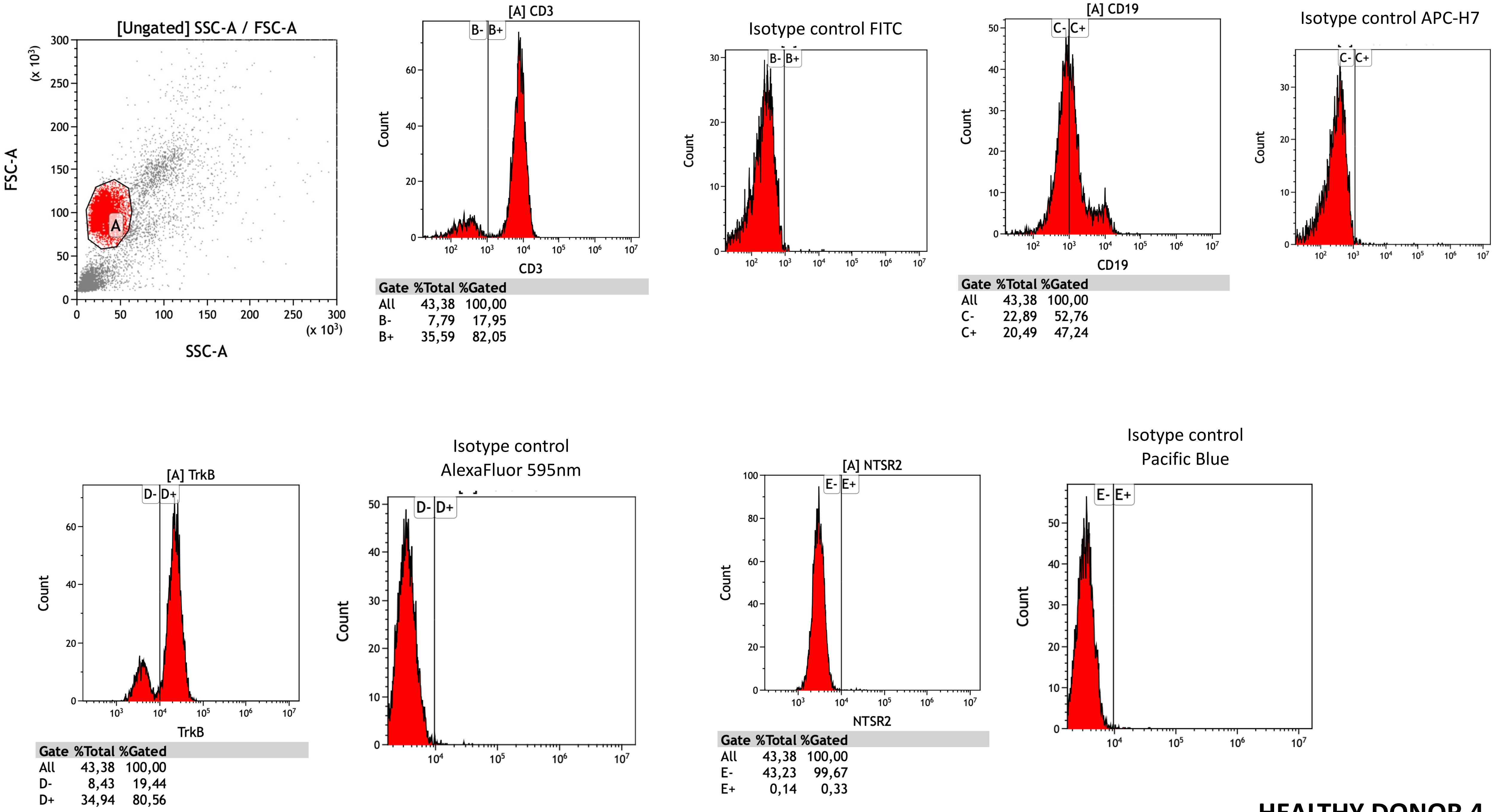


| Gate | %Total | %Gated |
|---|---|---|
| All | 43,38 | 100,00 |
| B- | 7,79 | 17,95 |
| B+ | 35,59 | 82,05 |

| Gate | %Total | %Gated |
|---|---|---|
| All | 43,38 | 100,00 |
| C- | 22,89 | 52,76 |
| C+ | 20,49 | 47,24 |

| Gate | %Total | %Gated |
|---|---|---|
| All | 43,38 | 100,00 |
| D- | 8,43 | 19,44 |
| D+ | 34,94 | 80,56 |

| Gate | %Total | %Gated |
|---|---|---|
| All | 43,38 | 100,00 |
| E- | 43,23 | 99,67 |
| E+ | 0,14 | 0,33 |

**HEALTHY DONOR 4**

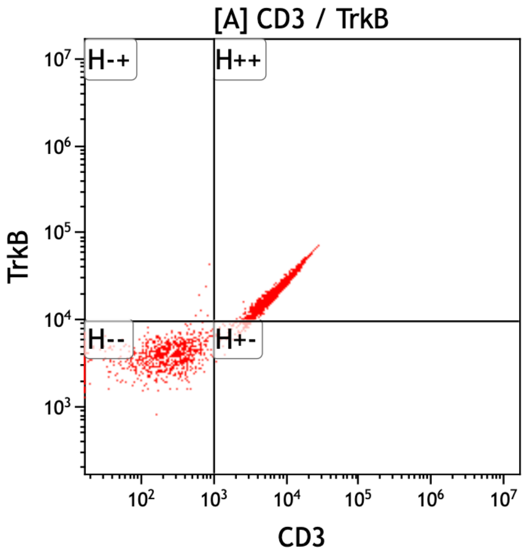


| Gate | %Gated |
|---|---|
| All | 100,00 |
| H-- | 17,84 |
| H-+ | 0,11 |
| H+- | 1,38 |
| H++ | 80,67 |

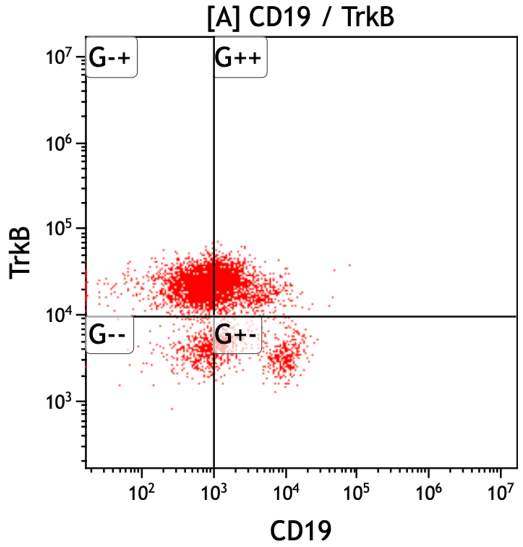


| Gate | %Gated |
|---|---|
| All | 100,00 |
| G-- | 7,14 |
| G-+ | 45,10 |
| G+- | 12,08 |
| G++ | 35,68 |

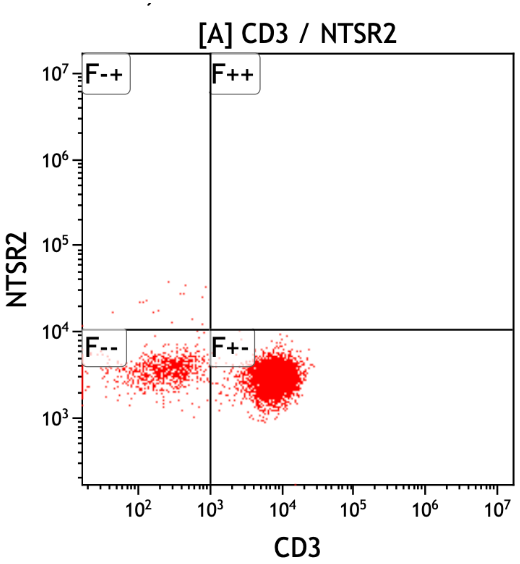


| Gate | %Gated |
|---|---|
| All | 100,00 |
| F-- | 17,64 |
| F-+ | 0,30 |
| F+- | 82,07 |
| F++ | 0,00 |

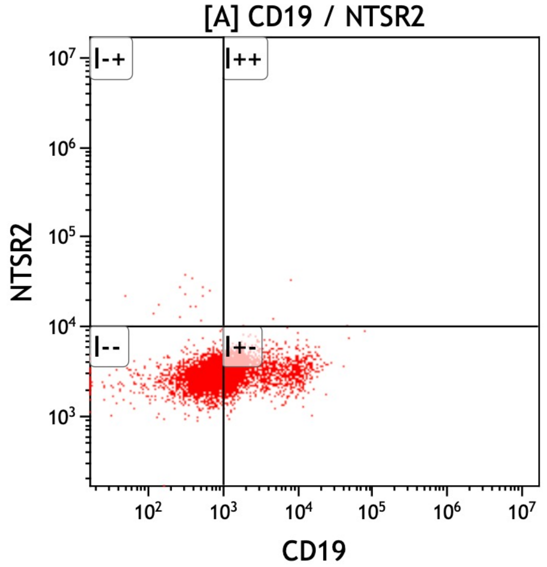


| Gate | %Gated |
|---|---|
| All | 100,00 |
| I-- | 52,43 |
| I-+ | 0,26 |
| I+- | 47,24 |
| I++ | 0,07 |

**HEALTHY DONOR 4**

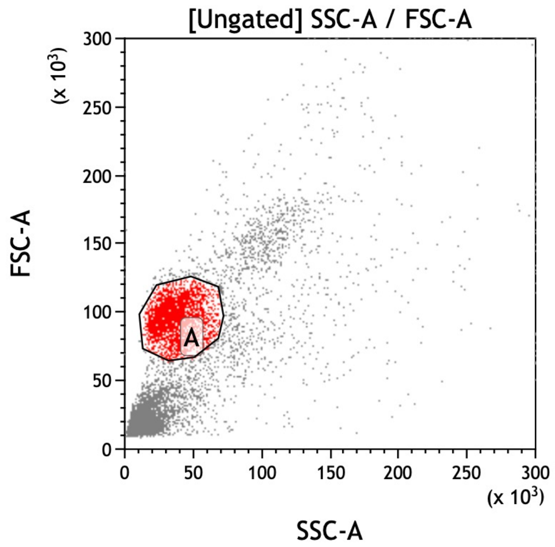


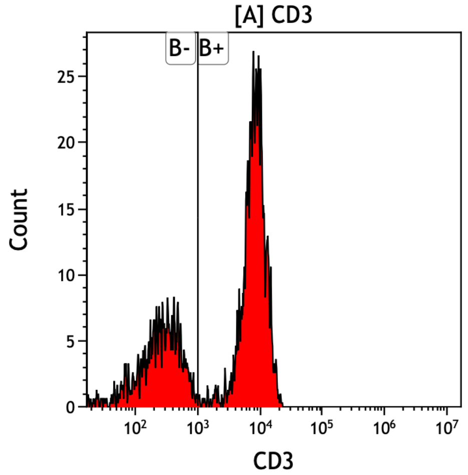


| Gate | %Total | %Gated |
|---|---|---|
| All | 26,55 | 100,00 |
| B- | 10,42 | 39,25 |
| B+ | 16,13 | 60,75 |

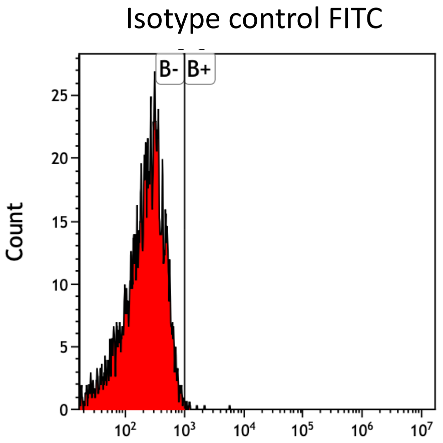


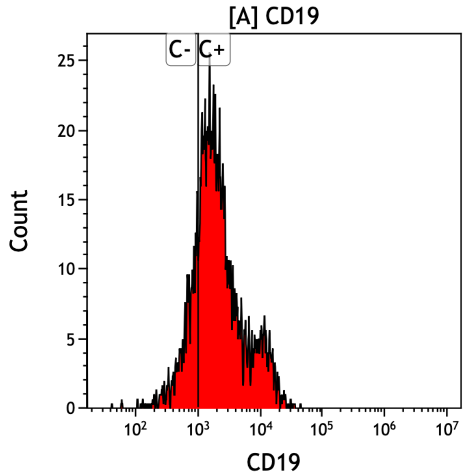


| Gate | %Total | %Gated |
|---|---|---|
| All | 26,55 | 100,00 |
| C- | 4,95 | 18,64 |
| C+ | 21,60 | 81,36 |

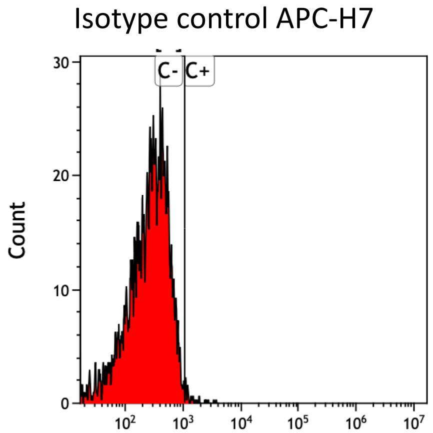


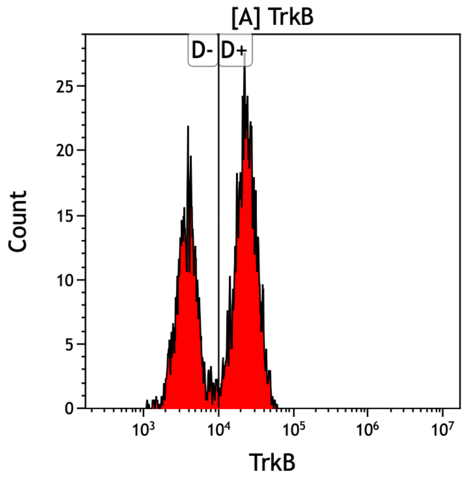


| Gate | %Total | %Gated |
|---|---|---|
| All | 26,55 | 100,00 |
| D- | 10,94 | 41,21 |
| D+ | 15,61 | 58,79 |

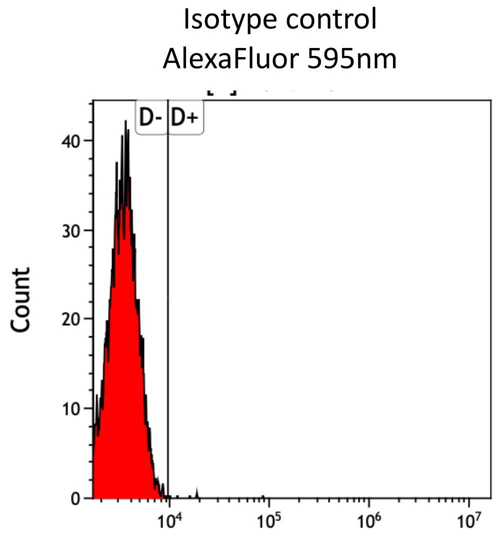


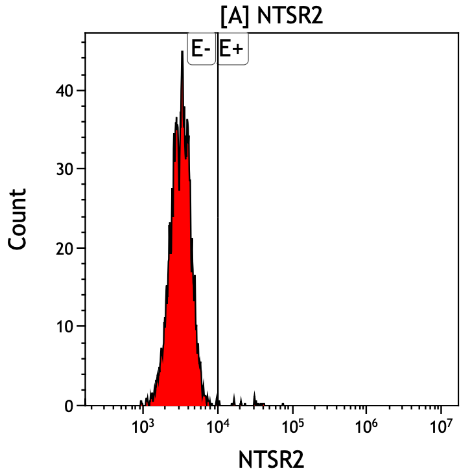


| Gate | %Total | %Gated |
|---|---|---|
| All | 26,55 | 100,00 |
| E- | 26,35 | 99,25 |
| E+ | 0,20 | 0,75 |

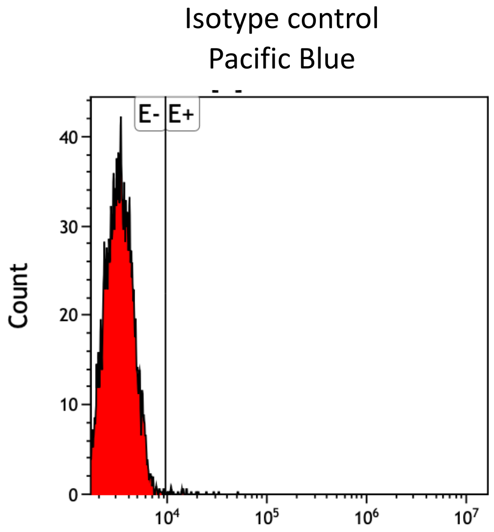


**HEALTHY DONOR 5**

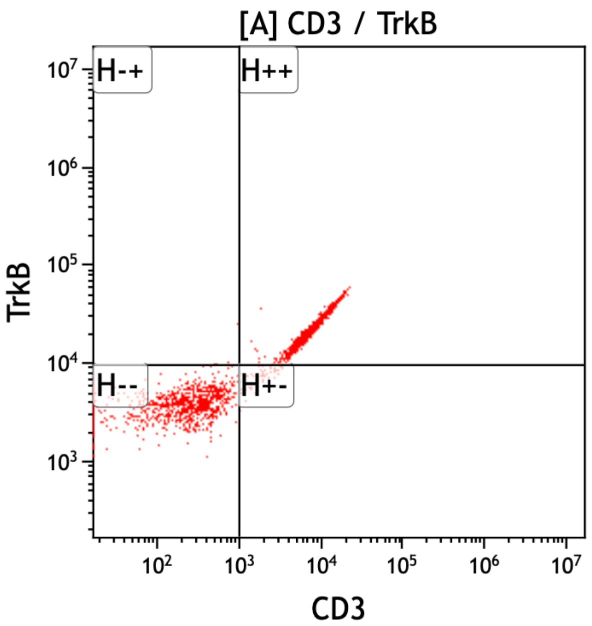


| Gate | %Gated |
|---|---|
| All | 100,00 |
| H-- | 39,17 |
| H-+ | 0,08 |
| H+- | 1,88 |
| H++ | 58,87 |

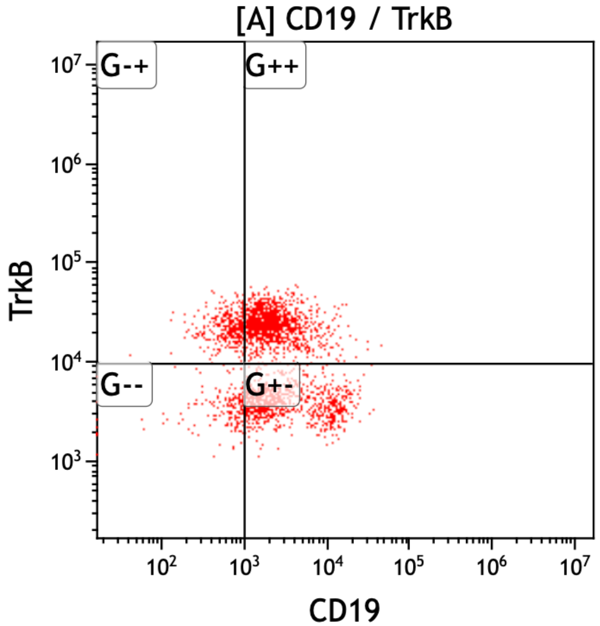


| Gate | %Gated |
|---|---|
| All | 100,00 |
| G-- | 6,33 |
| G-+ | 12,66 |
| G+- | 34,73 |
| G++ | 46,29 |

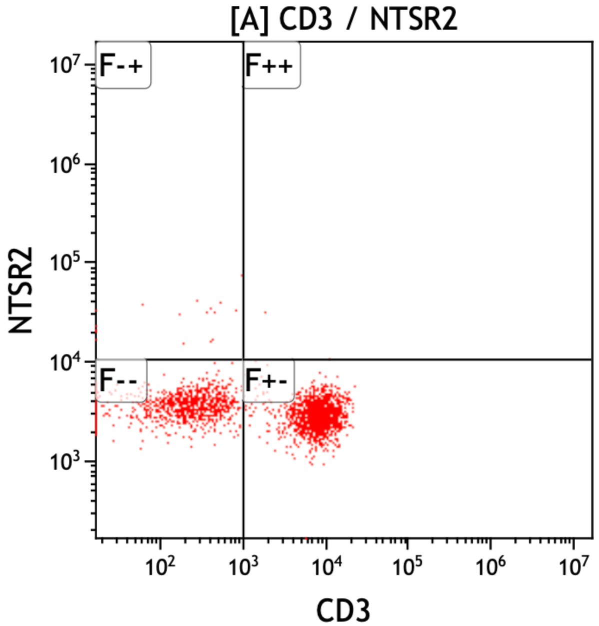


| Gate | %Gated |
|---|---|
| All | 100,00 |
| F-- | 38,61 |
| F-+ | 0,60 |
| F+- | 60,72 |
| F++ | 0,08 |

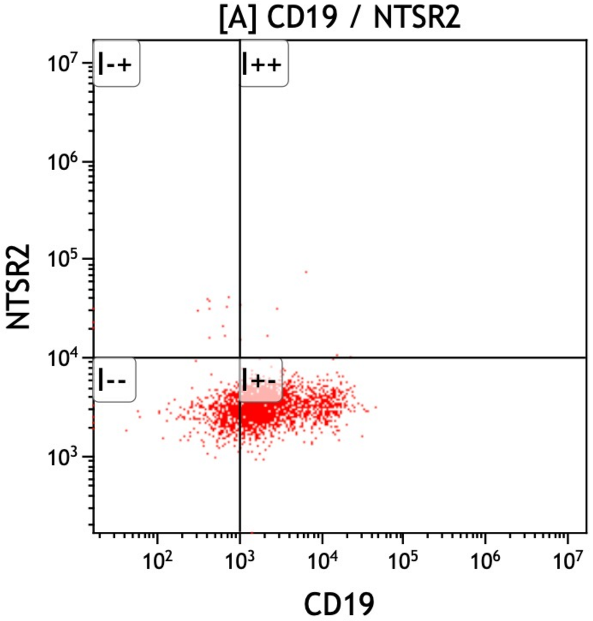


| Gate | %Gated |
|---|---|
| All | 100,00 |
| I-- | 18,68 |
| I-+ | 0,49 |
| I+- | 80,56 |
| I++ | 0,26 |

**HEALTHY DONOR 5**

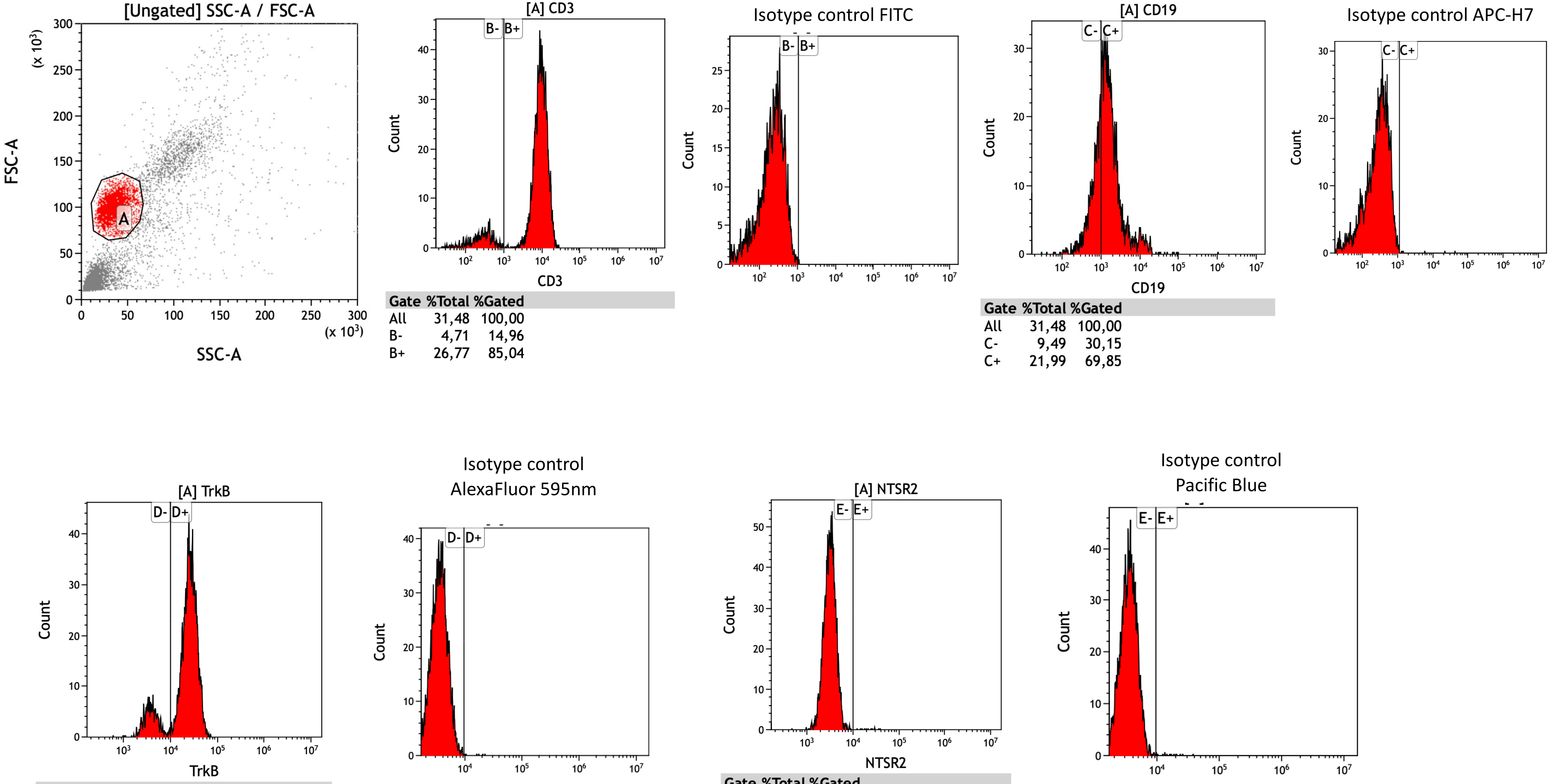


| Gate | %Total | %Gated |
|---|---|---|
| All | 31,48 | 100,00 |
| B- | 4,71 | 14,96 |
| B+ | 26,77 | 85,04 |

| Gate | %Total | %Gated |
|---|---|---|
| All | 31,48 | 100,00 |
| C- | 9,49 | 30,15 |
| C+ | 21,99 | 69,85 |

| Gate | %Total | %Gated |
|---|---|---|
| All | 31,48 | 100,00 |
| D- | 4,93 | 15,66 |
| D+ | 26,55 | 84,34 |

| Gate | %Total | %Gated |
|---|---|---|
| All | 31,48 | 100,00 |
| E- | 31,36 | 99,62 |
| E+ | 0,12 | 0,38 |

**HEALTHY DONOR 6**

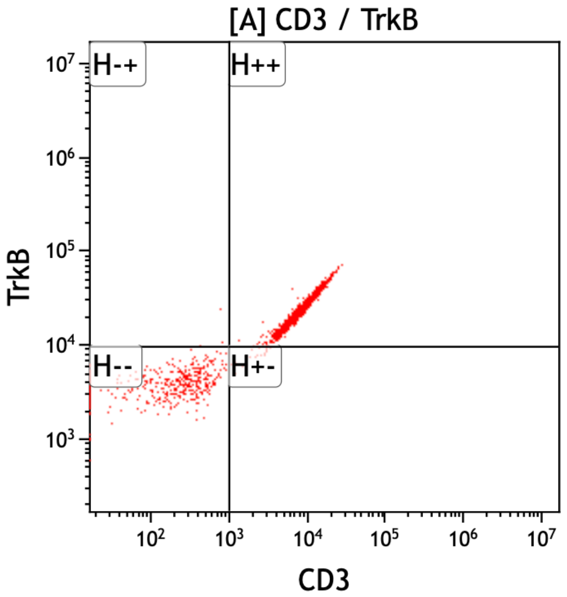


| Gate | %Gated |
|---|---|
| All | 100,00 |
| H-- | 14,90 |
| H-+ | 0,06 |
| H+- | 0,70 |
| H++ | 84,34 |

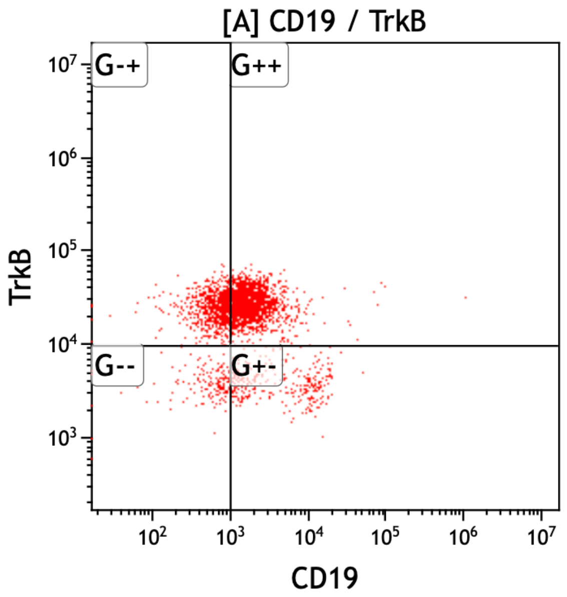


| Gate | %Gated |
|---|---|
| All | 100,00 |
| G-- | 4,89 |
| G-+ | 24,87 |
| G+- | 10,71 |
| G++ | 59,53 |

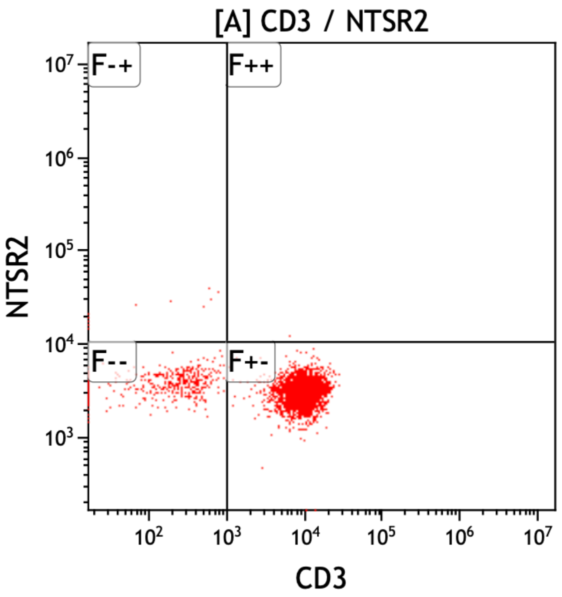


| Gate | %Gated |
|---|---|
| All | 100,00 |
| F-- | 14,61 |
| F-+ | 0,35 |
| F+- | 85,01 |
| F++ | 0,03 |

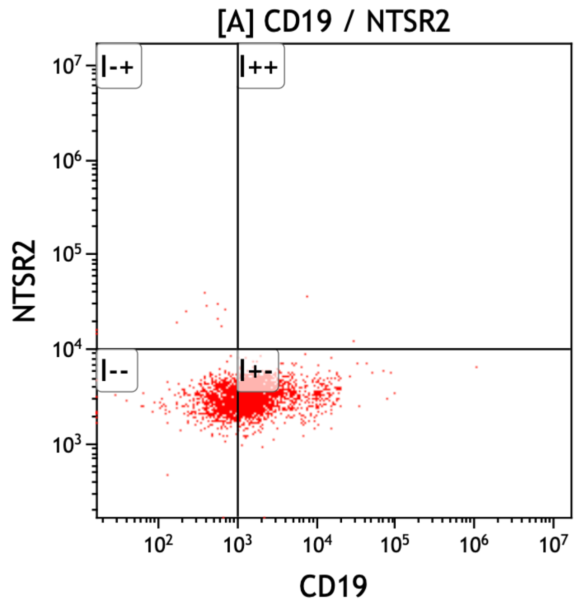


| Gate | %Gated |
|---|---|
| All | 100,00 |
| I-- | 29,80 |
| I-+ | 0,32 |
| I+- | 69,82 |
| I++ | 0,06 |

**HEALTHY DONOR 6**

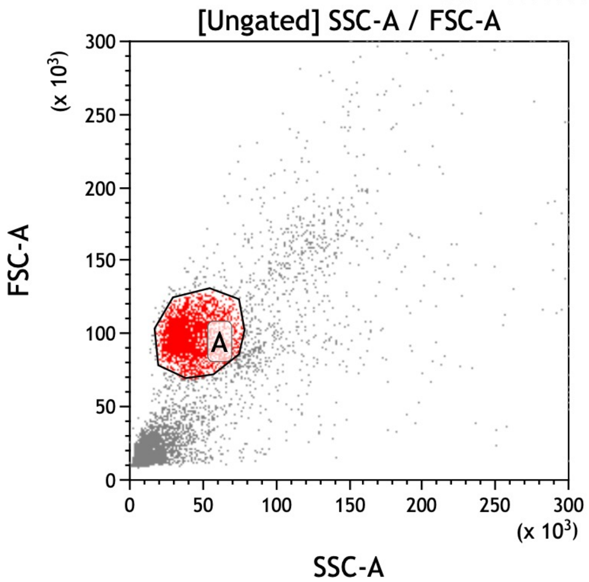


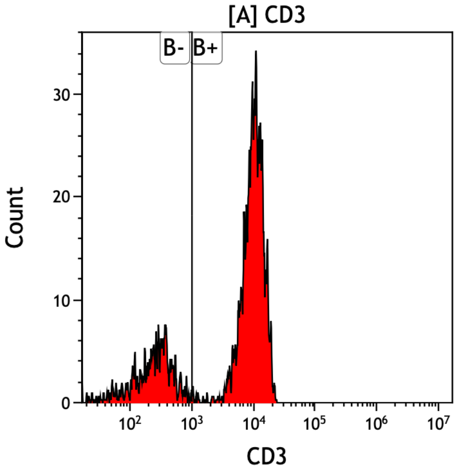


| Gate | %Total | %Gated |
|---|---|---|
| All | 31,82 | 100,00 |
| B- | 9,20 | 28,91 |
| B+ | 22,62 | 71,09 |

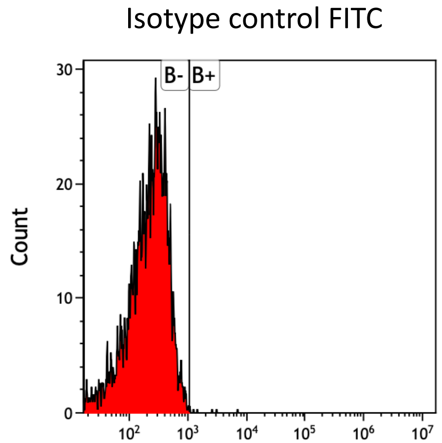


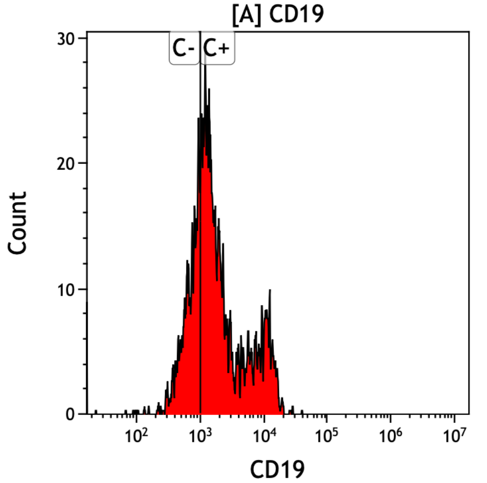


| Gate | %Total | %Gated |
|---|---|---|
| All | 31,82 | 100,00 |
| C- | 9,16 | 28,80 |
| C+ | 22,66 | 71,20 |

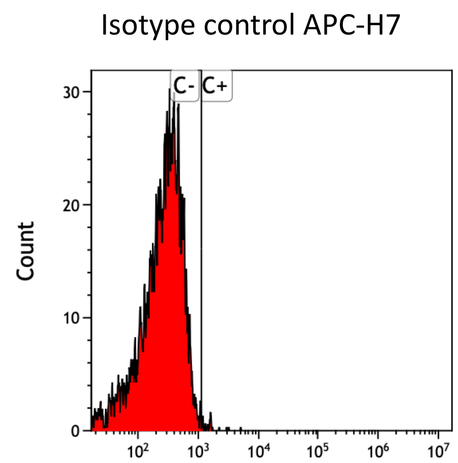


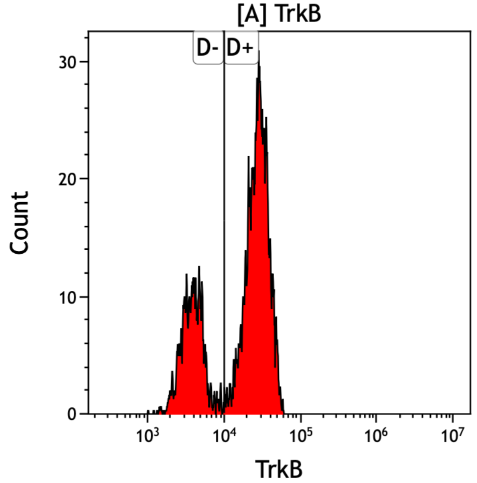


| Gate | %Total | %Gated |
|---|---|---|
| All | 31,82 | 100,00 |
| D- | 9,62 | 30,23 |
| D+ | 22,20 | 69,77 |

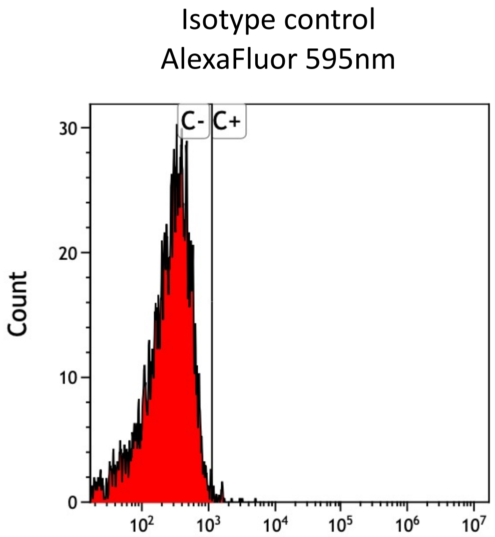


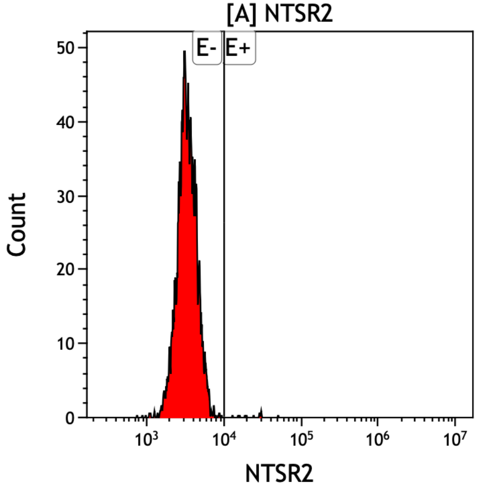


| Gate | %Total | %Gated |
|---|---|---|
| All | 31,82 | 100,00 |
| E- | 31,67 | 99,55 |
| E+ | 0,14 | 0,45 |

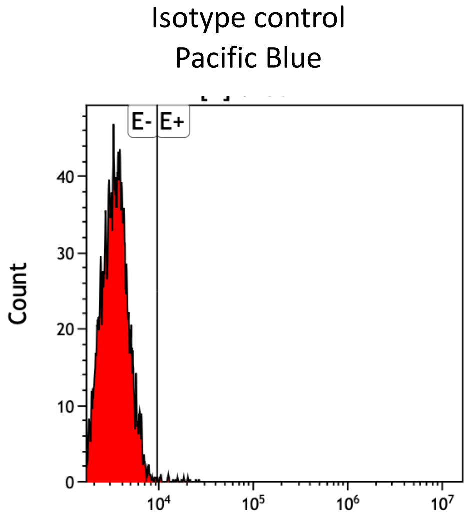


**HEALTHY DONOR 7**

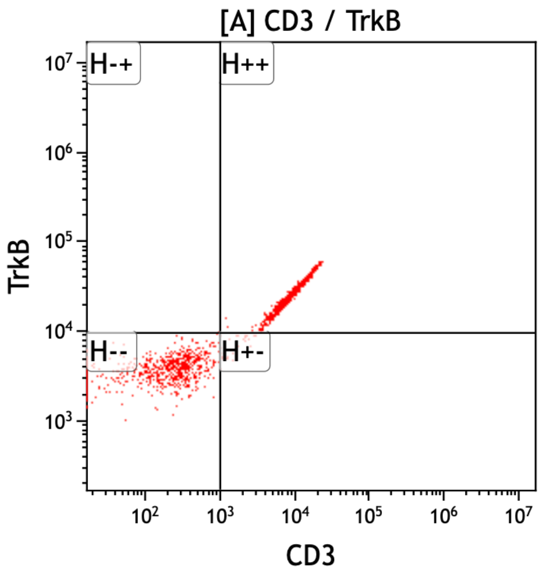


| Gate | %Gated |
|---|---|
| All | 100,00 |
| H-- | 28,95 |
| H-+ | 0,04 |
| H+- | 1,05 |
| H++ | 69,96 |

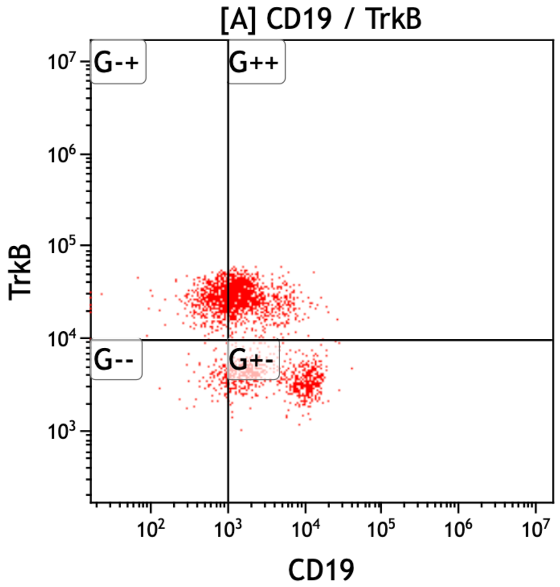


| Gate | %Gated |
|---|---|
| All | 100,00 |
| G-- | 3,38 |
| G-+ | 25,11 |
| G+- | 26,62 |
| G++ | 44,89 |

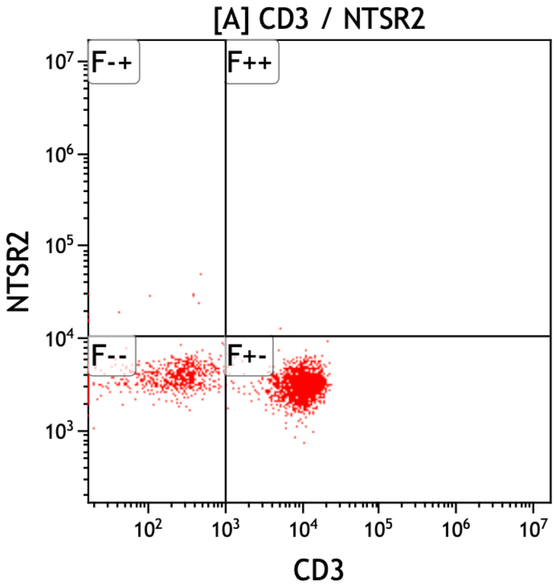


| Gate | %Gated |
|---|---|
| All | 100,00 |
| F-- | 28,50 |
| F-+ | 0,41 |
| F+- | 71,05 |
| F++ | 0,04 |

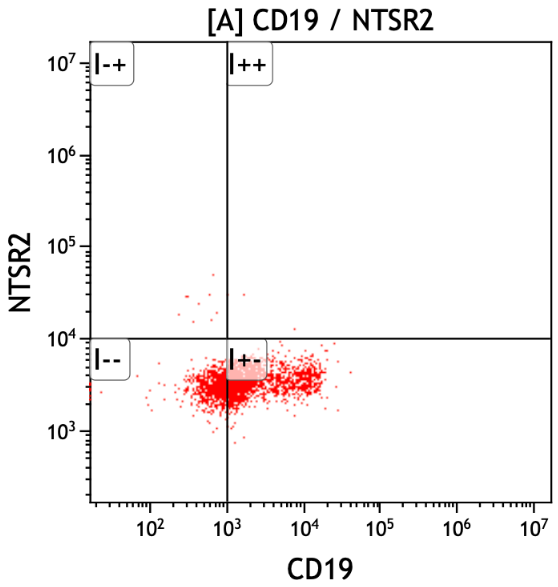


| Gate | %Gated |
|---|---|
| All | 100,00 |
| I-- | 28,38 |
| I-+ | 0,34 |
| I+- | 71,17 |
| I++ | 0,11 |

**HEALTHY DONOR 7**

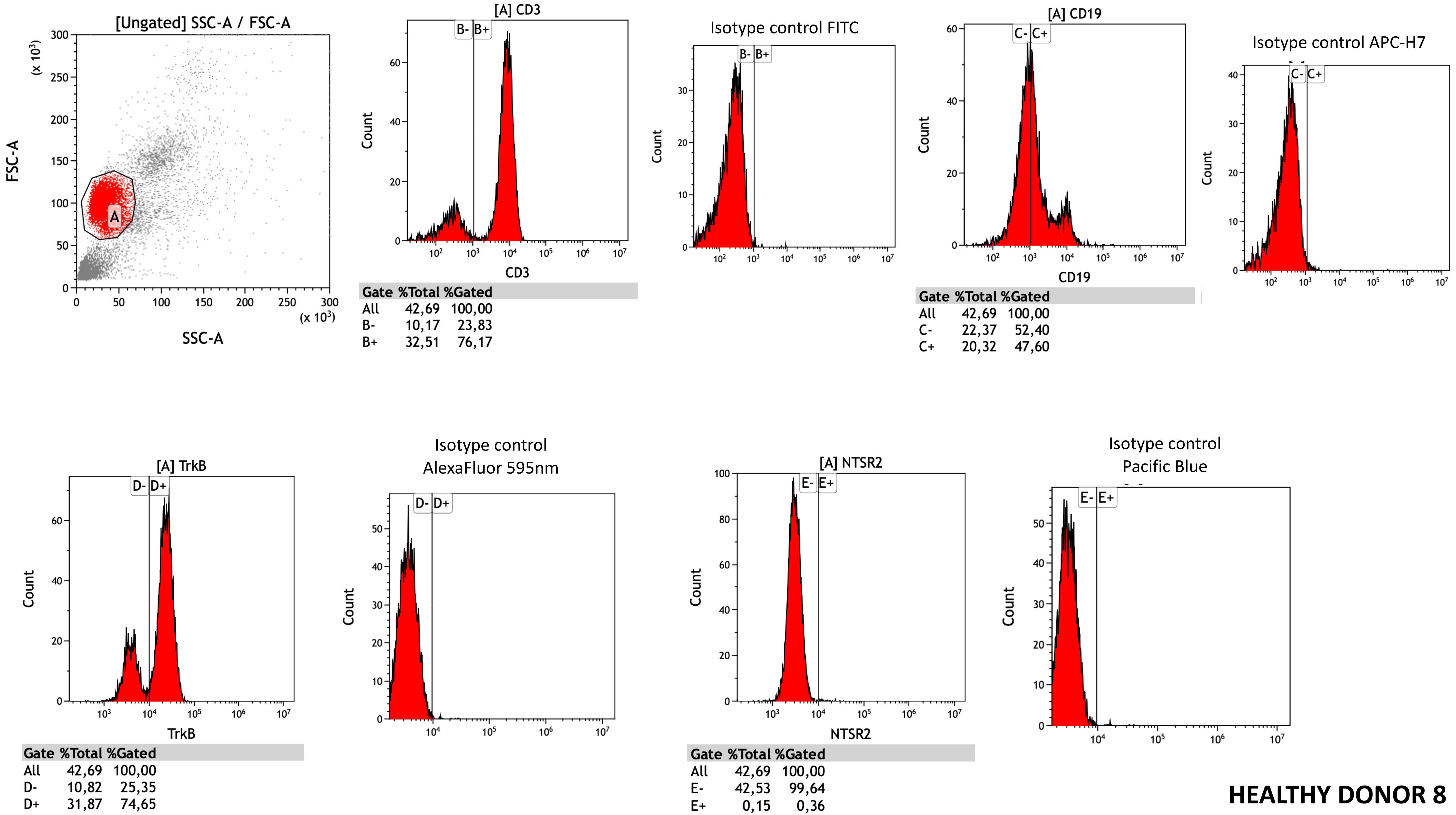
[Ungated] SSC-A / FSC-A
FSC-A
SSC-A
(x 10^3)
A
[A] CD3
CD3
Count
B- B+
Gate %Total %Gated
All 42,69 100,00
B- 10,17 23,83
B+ 32,51 76,17
Isotype control FITC
[A] CD19
CD19
C- C+
Gate %Total %Gated
All 42,69 100,00
C- 22,37 52,40
C+ 20,32 47,60
Isotype control APC-H7
[A] TrkB
TrkB
D- D+
Gate %Total %Gated
All 42,69 100,00
D- 10,82 25,35
D+ 31,87 74,65
Isotype control
AlexaFluor 595nm
[A] NTSR2
NTSR2
E- E+
Gate %Total %Gated
All 42,69 100,00
E- 42,53 99,64
E+ 0,15 0,36
Isotype control
Pacific Blue
HEALTHY DONOR 8

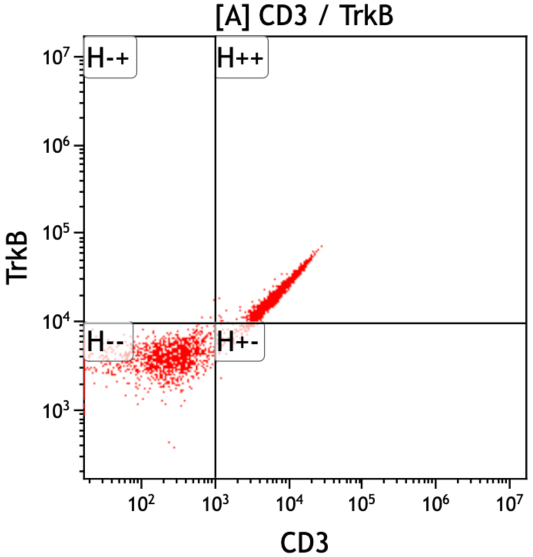


| Gate | %Gated |
|---|---|
| All | 100,00 |
| H-- | 23,74 |
| H-+ | 0,05 |
| H+- | 1,34 |
| H++ | 74,87 |

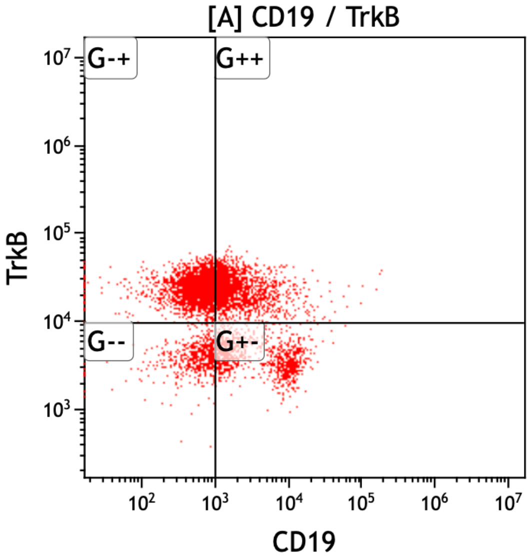


| Gate | %Gated |
|---|---|
| All | 100,00 |
| G-- | 8,82 |
| G-+ | 39,51 |
| G+- | 16,26 |
| G++ | 35,41 |

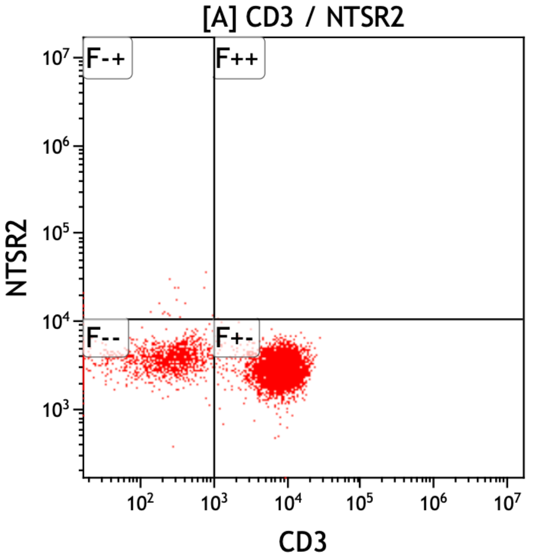


| Gate | %Gated |
|---|---|
| All | 100,00 |
| F-- | 23,43 |
| F-+ | 0,33 |
| F+- | 76,21 |
| F++ | 0,03 |

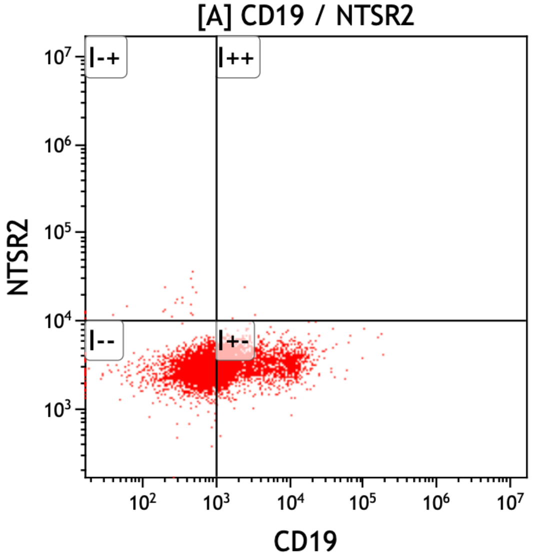


| Gate | %Gated |
|---|---|
| All | 100,00 |
| I-- | 48,34 |
| I-+ | 0,31 |
| I+- | 51,30 |
| I++ | 0,05 |

**HEALTHY DONOR 8**

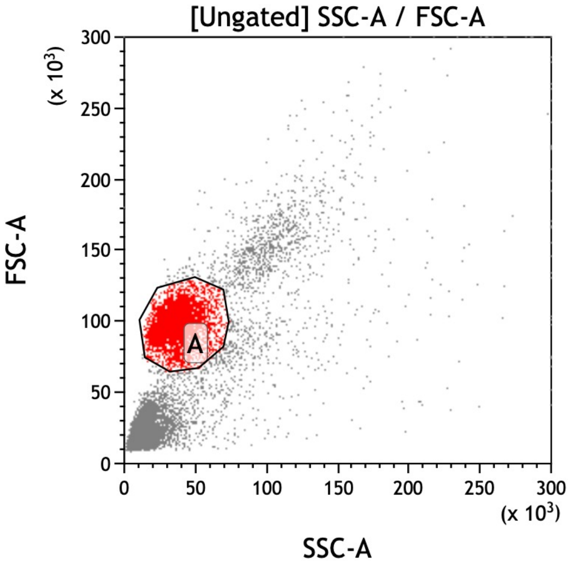


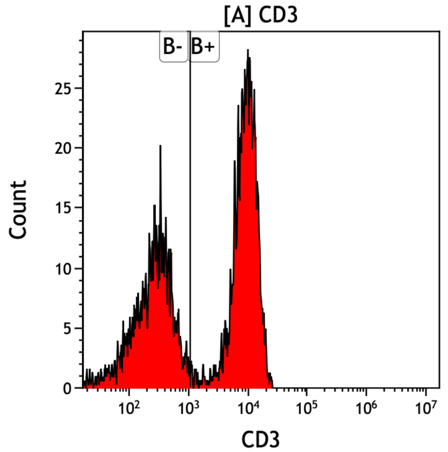


| Gate | %Total | %Gated |
| --- | --- | --- |
| All | 25,66 | 100,00 |
| B- | 12,46 | 48,56 |
| B+ | 13,20 | 51,44 |

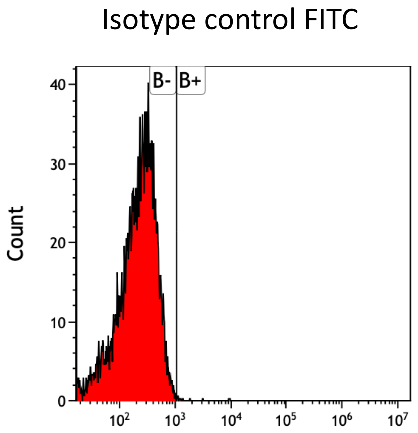


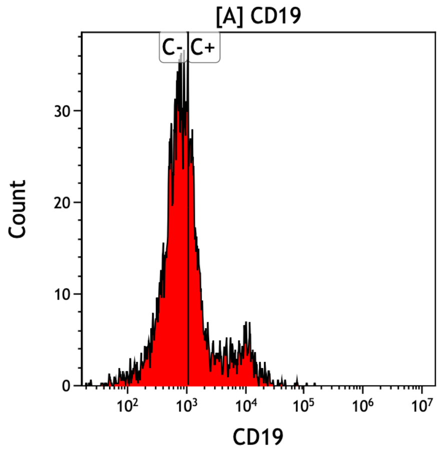


| Gate | %Total | %Gated |
| --- | --- | --- |
| All | 25,66 | 100,00 |
| C- | 16,13 | 62,87 |
| C+ | 9,53 | 37,13 |

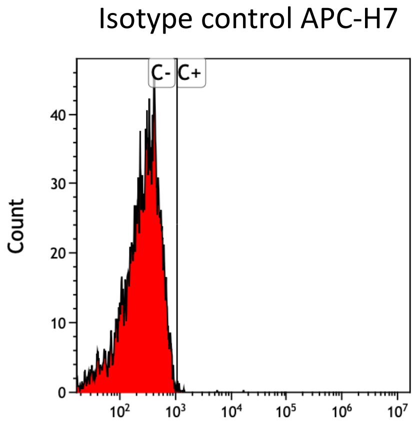


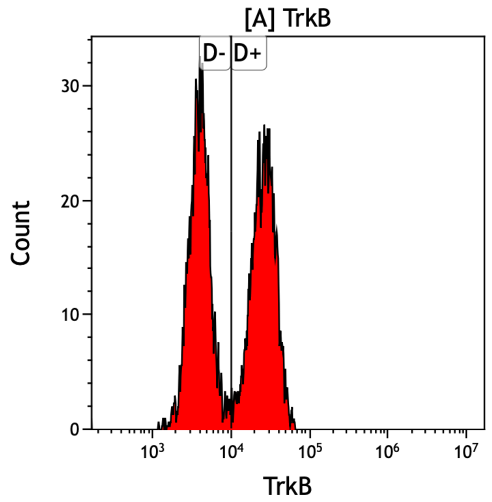


| Gate | %Total | %Gated |
| --- | --- | --- |
| All | 25,66 | 100,00 |
| D- | 12,98 | 50,58 |
| D+ | 12,68 | 49,42 |

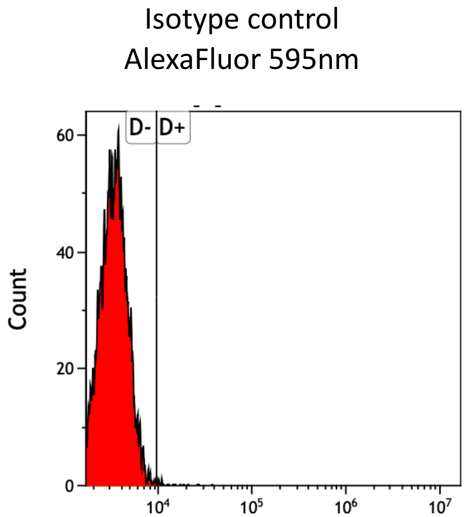


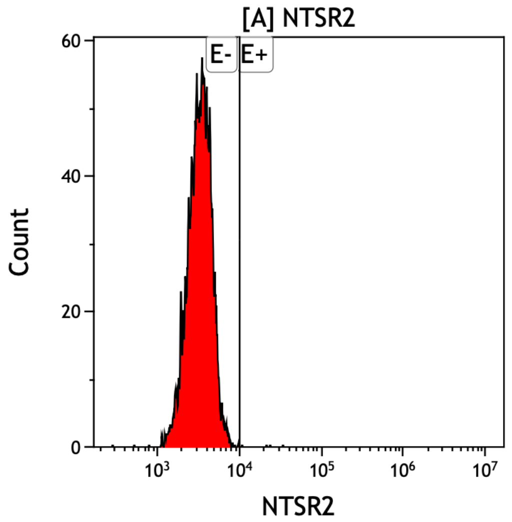


| Gate | %Total | %Gated |
| --- | --- | --- |
| All | 25,66 | 100,00 |
| E- | 25,63 | 99,87 |
| E+ | 0,03 | 0,13 |

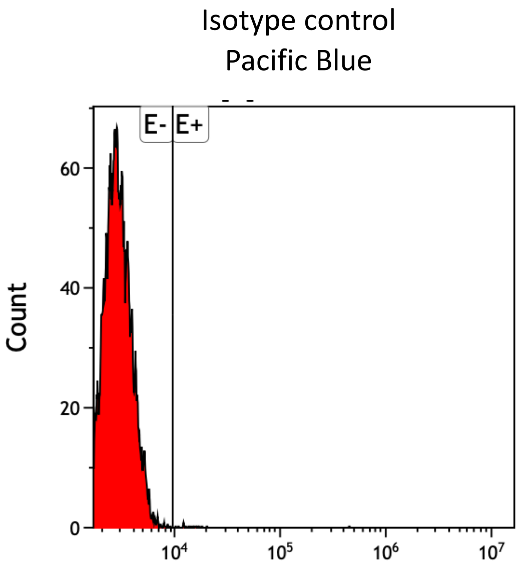


**HEALTHY DONOR 9**

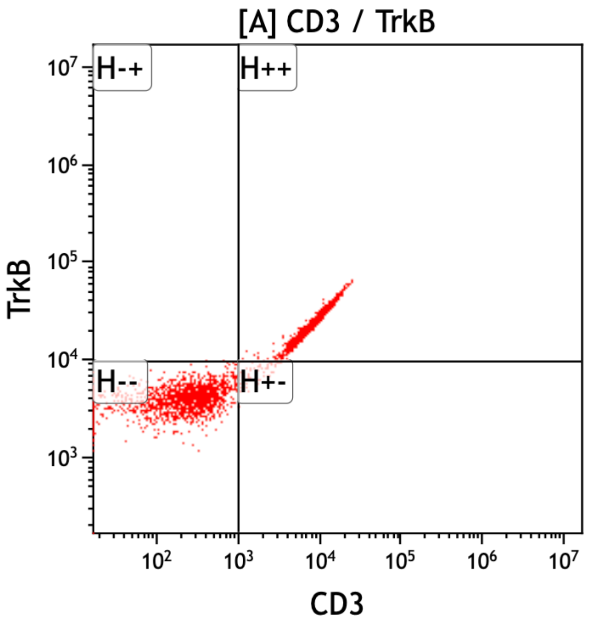


| Gate | %Gated |
|---|---|
| All | 100,00 |
| H-- | 48,48 |
| H-+ | 0,03 |
| H+- | 1,84 |
| H++ | 49,65 |

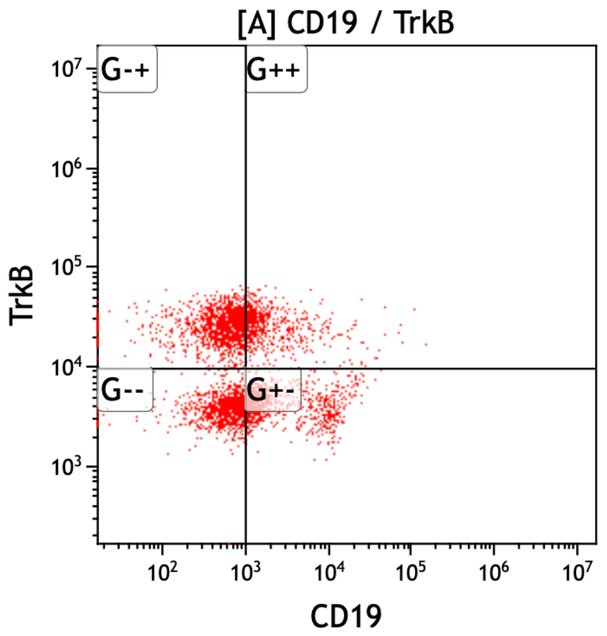


| Gate | %Gated |
|---|---|
| All | 100,00 |
| G-- | 25,82 |
| G-+ | 32,97 |
| G+- | 24,50 |
| G++ | 16,71 |

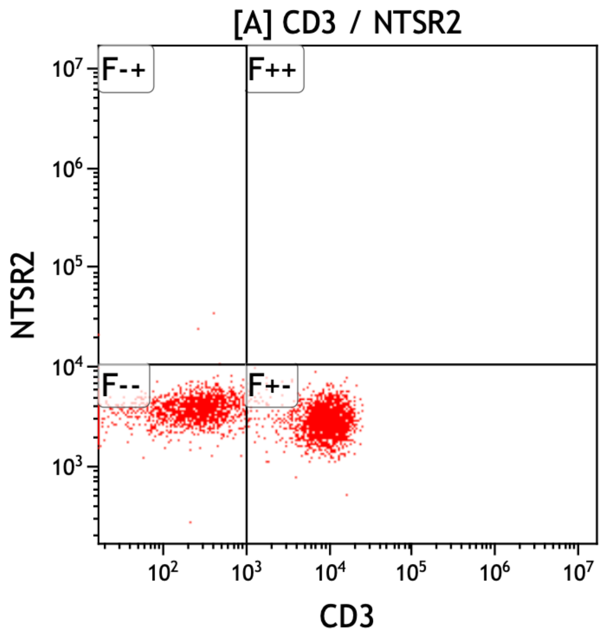


| Gate | %Gated |
|---|---|
| All | 100,00 |
| F-- | 48,32 |
| F-+ | 0,10 |
| F+- | 51,57 |
| F++ | 0,00 |

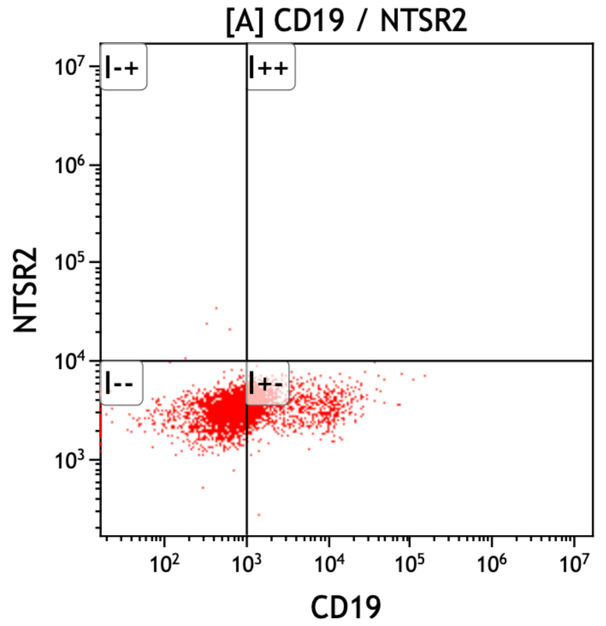


| Gate | %Gated |
|---|---|
| All | 100,00 |
| I-- | 59,16 |
| I-+ | 0,13 |
| I+- | 40,71 |
| I++ | 0,00 |

**HEALTHY DONOR 9**

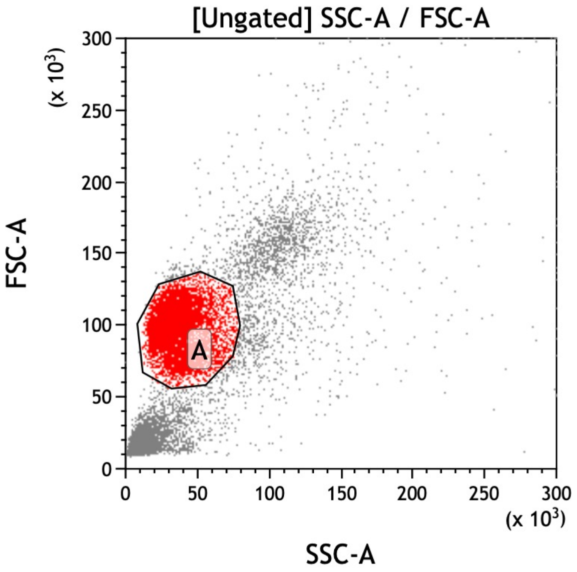


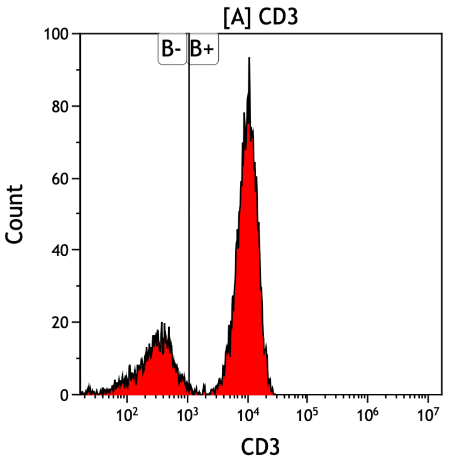


| Gate | %Total | %Gated |
|---|---|---|
| All | 51,45 | 100,00 |
| B- | 14,75 | 28,67 |
| B+ | 36,70 | 71,33 |

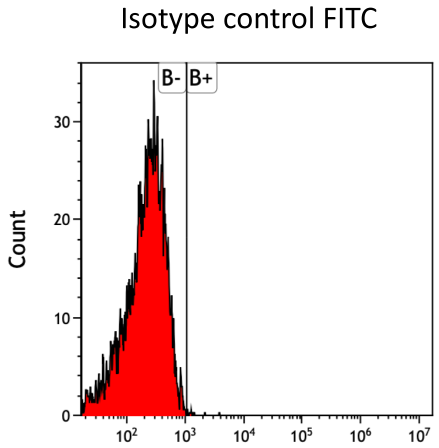


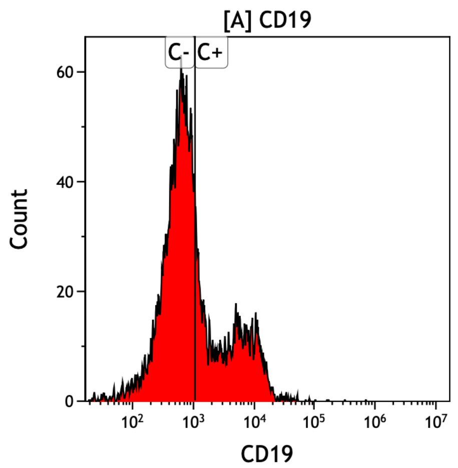


| Gate | %Total | %Gated |
|---|---|---|
| All | 51,45 | 100,00 |
| C- | 34,00 | 66,08 |
| C+ | 17,45 | 33,92 |

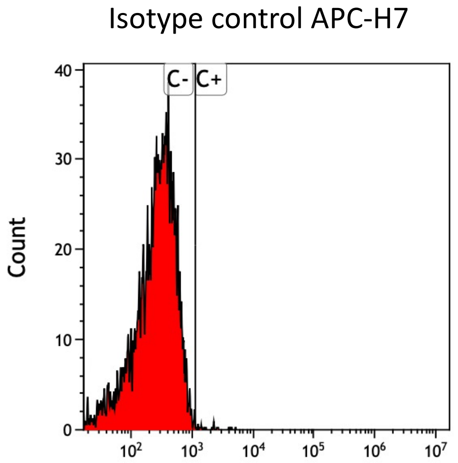


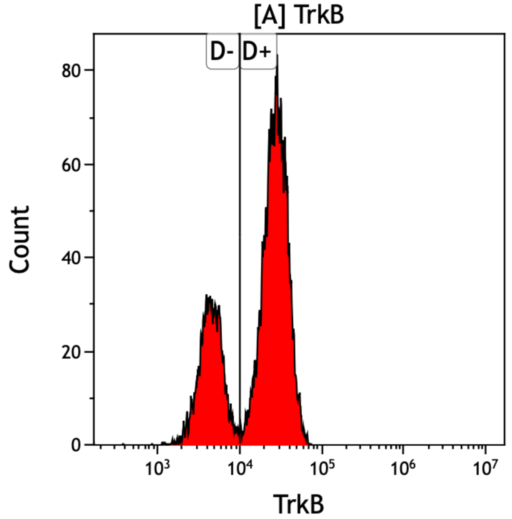


| Gate | %Total | %Gated |
|---|---|---|
| All | 51,45 | 100,00 |
| D- | 15,20 | 29,54 |
| D+ | 36,25 | 70,46 |

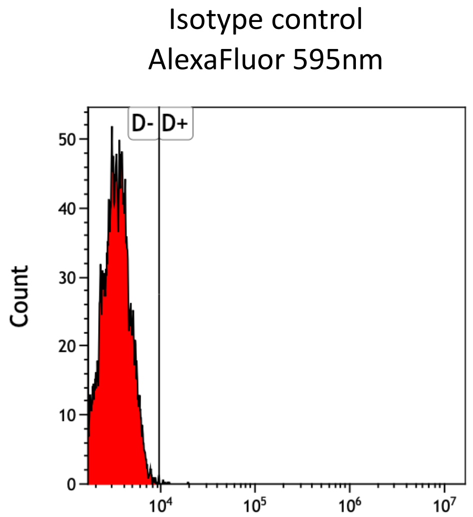


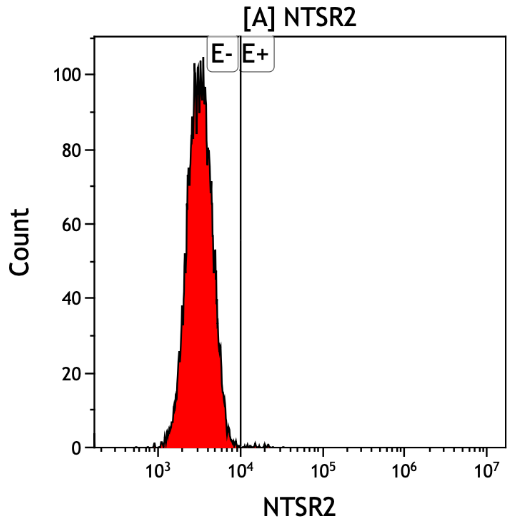


| Gate | %Total | %Gated |
|---|---|---|
| All | 51,45 | 100,00 |
| E- | 51,27 | 99,64 |
| E+ | 0,19 | 0,36 |

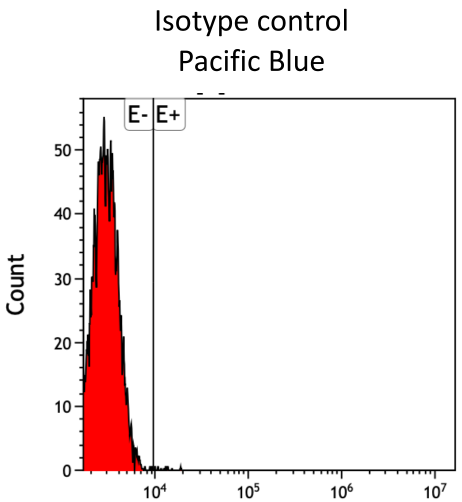


**HEALTHY DONOR 10**

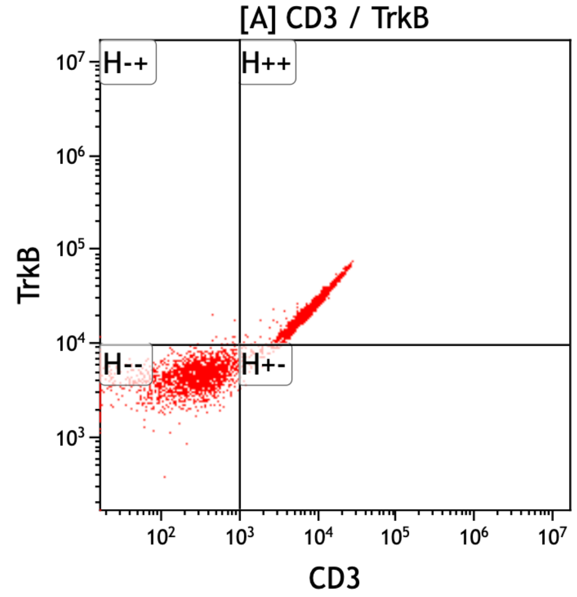


| Gate | %Gated |
|---|---|
| All | 100,00 |
| H-- | 28,45 |
| H-+ | 0,14 |
| H+- | 0,93 |
| H++ | 70,47 |

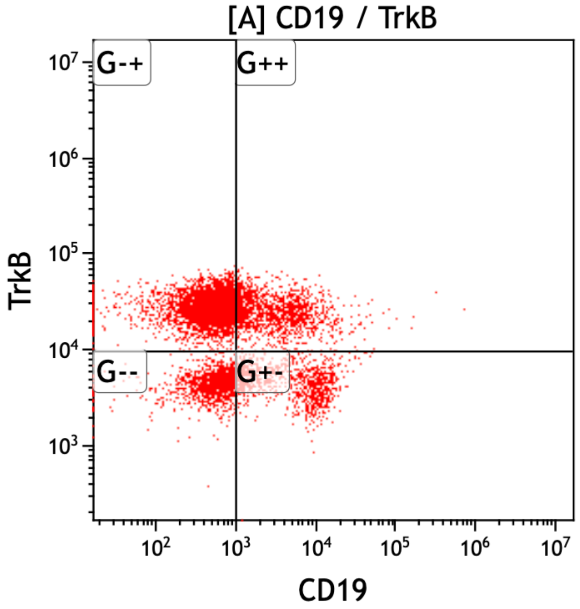


| Gate | %Gated |
|---|---|
| All | 100,00 |
| G-- | 14,99 |
| G-+ | 48,51 |
| G+- | 14,39 |
| G++ | 22,10 |

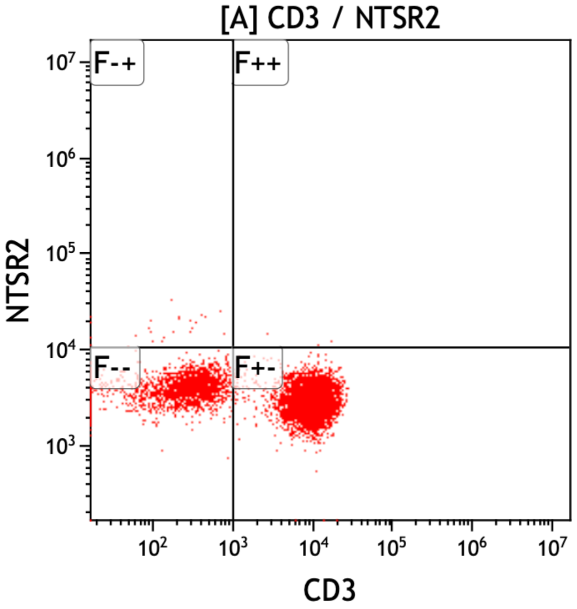


| Gate | %Gated |
|---|---|
| All | 100,00 |
| F-- | 28,25 |
| F-+ | 0,32 |
| F+- | 71,39 |
| F++ | 0,04 |

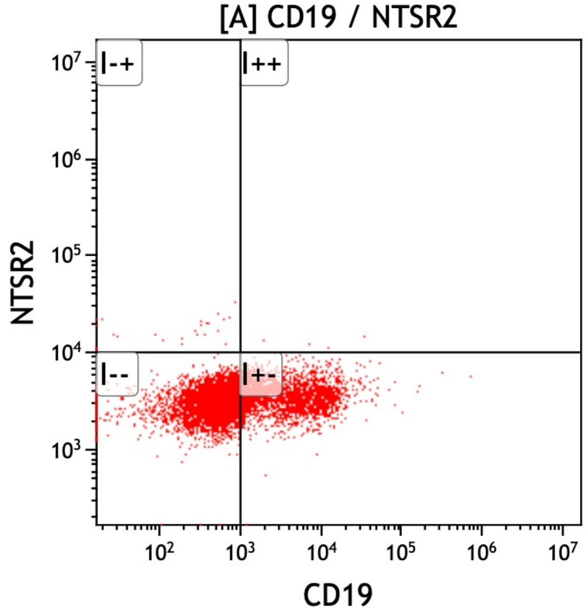


| Gate | %Gated |
|---|---|
| All | 100,00 |
| I-- | 63,47 |
| I-+ | 0,31 |
| I+- | 36,16 |
| I++ | 0,05 |

**HEALTHY DONOR 10**
