# Supplementary material for: Targeting the NTSR2/TrkB oncogenic pathway in chronic lymphocytic leukemia
Source: Sci Rep. 2024 Mar 13;14:6084. doi: 10.1038/s41598-024-56663-5 (PMC10937676; doi:10.1038/s41598-024-56663-5)
Supplement: Supplementary file 3 — Supplementary Information 3. [file 41598_2024_56663_MOESM3_ESM.pdf]

Targeting the NTSR2/TrkB oncogenic pathway in Chronic Lymphocytic Leukemia

Léa Ikhlef[a], May Yassine[a], Boutaîna Chandouri-Faize[a], Léa Rivière[a], Thomas Naves[a], Natalya Dmytruk[b], Nathalie Gachard[c], Marie-Odile Jauberteau[a,d] and Paul-François Gallet[a,#]

[a] *University of Limoges, UMR INSERM 1308, CAPTuR, Limoges, France*
[b] *Department of Clinical Hematology, University Hospital of Limoges, France*
[c] *Hematology laboratory, UMR CNRS7276/ INSERM 1262, University Hospital of Limoges, Limoges, France*
[d] *Immunology laboratory, University Hospital of Limoges, Limoges, France*

Corresponding author: [#] Paul-François GALLET, UMR INSERM 1308 CAPTuR, Faculté de Médecine, 2 rue du Docteur Marcland, 87025 Limoges Cedex, France; email : francois.gallet@unilim.fr

Supplementary tables

Supplementary Table 1. Characteristics of the CLL patients within the cohort.

Patient ID number	Binet stage	% B Cells	% CD19/CD5	cytogenetics	TP53	IGHV mutation	Chromosome 2 aberration	Total prognosis (cytogenetics and/or mutations)
1	C	98.5	100	Favorable del13q	unmutated	mutated, favorable	no	not adverse
2	B	90	99	Adverse del13q del17p	mutated and deleted (R181C ; R342*)	unmutated, adverse	no	adverse
3	A	78	98	ND	ND	ND	ND	/
4	B	92	99	Adverse del11q del6q	unmutated	unmutated, adverse	no	adverse
5	A	85	100	ND	ND	ND	ND	ND
6	C	ND	ND	Adverse +12, del14q	mutated (R273C ; G244D)	unmutated, adverse	XPO1 mutation	adverse
7	C	ND	ND	adverse del17p (TP53) del13q	mutated and deleted (C135Y)	mutated, favorable	no	adverse
8	C	90	96	Favorable del13q	unmutated	unmutated, adverse	no	adverse
9	C	90	97	Intermediate +12	mutated	unmutated, adverse	no	adverse
10	A	88	96	Favorable del13q	ND	mutated, favorable	ND	not adverse
11	C	94	100	Adverse del6q, del13q, del17p	mutated	unmutated, adverse	no	adverse
12	B	97	100	Adverse Complex >3	mutated	unmutated, adverse	no	adverse
13	C	71	100	Adverse Complex >5	ND	unmutated, adverse	ND	ND
14	C	91	100	Intermediate del14q	unmutated	unmutated, adverse	no	intermediate
15	C	36	56	Adverse Complex >5	unmutated	unmutated, adverse	no	adverse
16	C	91	ND	Favorable del13q	unmutated	mutated, subset 2	no	intermediate
17	B	92	100	Favorable del13q	unmutated	mutated, favorable	no	not adverse
18	A	83	95	ND	ND	ND	ND	/
19	A	ND	ND	ND	ND	ND	ND	/
20	B	39	99	Favorable del13q	unmutated	unmutated, adverse	XPO1 mutation	intermediate
21	C	85	85	Favorable del13q	unmutated	unmutated, adverse	no	intermediate
22	A	64	100	Intermediate +12	unmutated	Mutated, favorable	no	not adverse
23	C	46	57	Intermediate del6q	unmutated	ND	no	not adverse
24	A	97	100	ND	ND	ND	ND	/
25	A	ND	ND	ND	ND	ND	ND	/
26	B	ND	ND	Favorable del13q	unmutated	ND	no	not adverse
27	A	65	ND	ND	ND	ND	ND	/
28	A	85	99	ND	ND	ND	ND	/
29	A	96	ND	ND	ND	ND	ND	/
30	A	94	100	ND	ND	unmutated, adverse	ND	/

Supplementary Table 2. List of the antibodies used for immunoblotting.

Target	Reference	Manufacturer
NTSR2	CSB-PA008417	Cusabio
TrkB	610101	BDbioscience
V5 Tag	R96025	ThermoFisher Scientific
P-Tyr	05-321	Sigma-Aldrich
Src	2123	Cell signaling

P-Src (Y416)	2101S	Cell signaling
AKT	2920S	Cell signaling
P-AKT (S473)	4060S	Cell signaling
Bcl-2	16810694	Proteintech
Bcl-xL	sc-8392	Santa Cruz
Mcl-1	4572	Cell signaling
beta-actin	A2066	ThermoFisher
Lyn	2796T	Cell signaling
P-Lyn (Tyr397)	BS-3257R	ThermoFisher
BTK	8547T	Cell signaling
P-BTK (Tyr551)	18805S	Cell signaling

Supplementary Table 3. List of the antibodies used for flow cytometry.

Target	Host species	Reference	Manufacturer
NTSR2	rabbit	CSB-PA008417	Cusabio
TrkB	mouse	610101	BDbioscience
TrkB	goat	AF1494-SP	R&D biosystems
Rabbit IgG Alexa Fluor 488	goat	A-11008	ThermoFisher
Mouse IgG Alexa Fluor 594	goat	A-11005	ThermoFisher
Goat IgG Alexa Fluor 594	donkey	A32758	ThermoFisher
Rabbit IgG Pacific Blue™	goat	P-10994	ThermoFisher
CD3 FITC	mouse	349201	BDbioscience
CD19 APC-H7	mouse	560727	BDbioscience

Supplementary figures

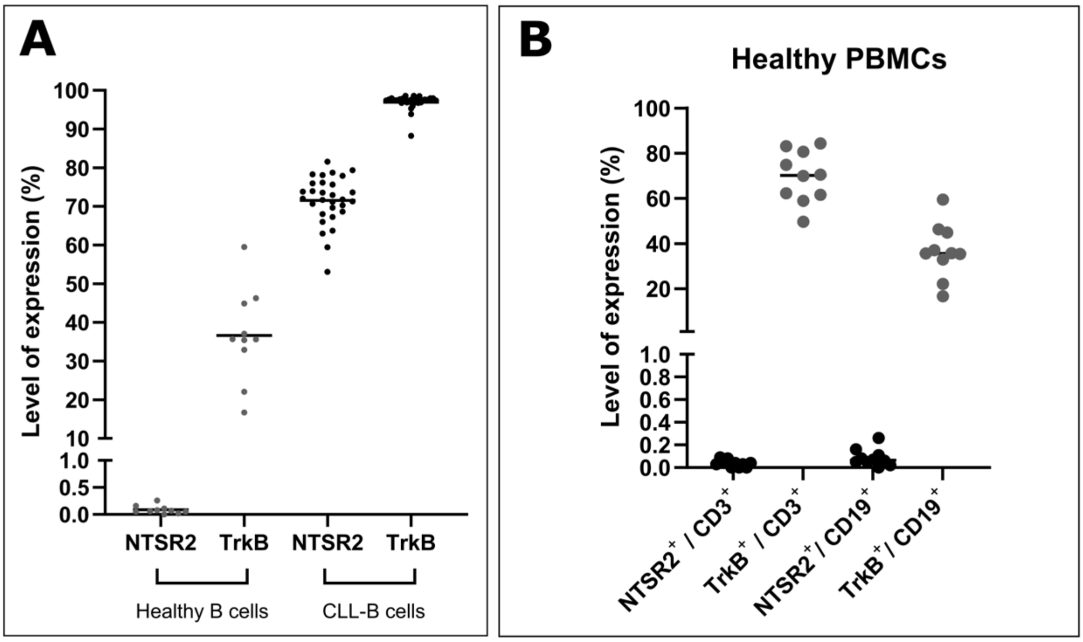

Figure S1. Expression of NTSR2 and TrkB in CLL-B cells and healthy B and T cells. (**A**) 100 000 B CLL cells per well were seeded in 96-V well plates and were first incubated with NTSR2 (#CSB-PA008417, Cusabio) and TrkB (#610101, BD biosciences) primary antibodies and then stained using Alexa Fluor coupled secondary antibodies (#A-11008 & #A-11005, ThermoFisher). Data for healthy B cells is extracted from the graph in B and corresponds to NTSR2^{+}CD19^{+} and TrkB^{+}CD19^{+} cells. (**B**) 100 000 healthy PBMCs per well were seeded in 96-V well plates and were first incubated with NTSR2 (#CSB-PA008417, Cusabio) and TrkB (#AF1494-SP, R&D biosystems) primary antibodies and then with using Alexa Fluor coupled secondary antibodies (#A32758 & #P-10994, ThermoFisher) as well as B cell marker anti-CD19 antibody (#560727, BD biosciences) and T cell marker anti-CD3 antibody (#349201, BD biosciences). They were fixed with 2% PFA for 15min at room temperature. Fluorescence was measured using the CytoFLEX LX device (Beckman Coulter) and data analyzed using the Kaluza software (Beckman Coulter). For analyses on PBMCs, lymphocytes were gated using the Kaluza software and fluorescence was measured within gated cells.

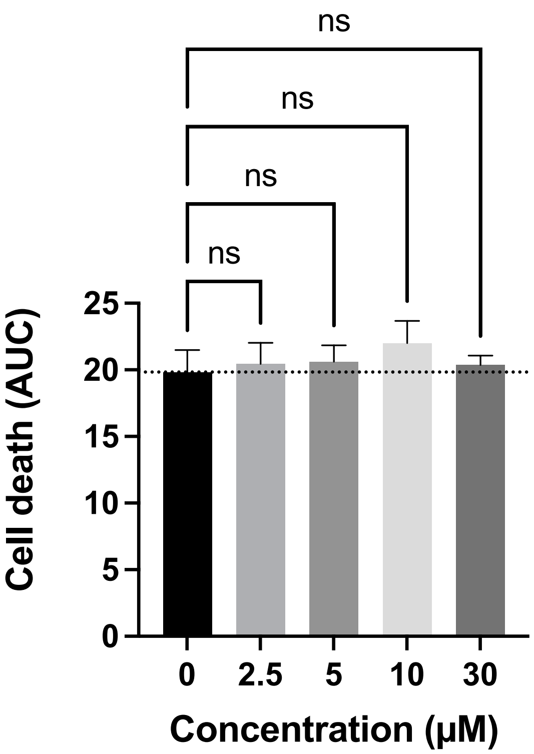

Figure S2. Cytotoxicity of the TAT peptide on patients' B cells. Experiments were conducted on 10 different patients, each time in technical triplicates. Experiments were conducted using the Incucyte live-cell imaging device, cells were incubated with Cytotox Dye (green), fluorescence was monitored for 48h. 'Green Object Count / Phase Object Count' ratio was determined using the Incucyte analysis software to obtain the percentage of dead cells. The area under curve of each condition was then calculated and they were compared with a t-test.

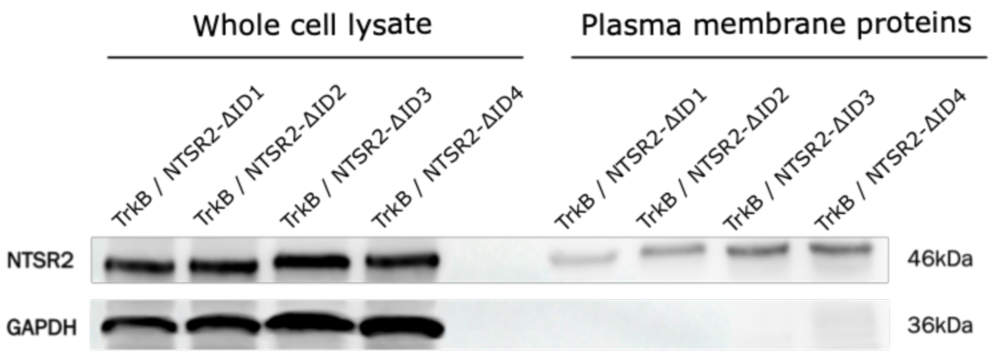

Figure S3. Control of the cellular location of the NTSR2 intracellular mutants. Representative immunoblot of 3 independent experiments. To verify that the absence of interaction between NTSR2 and TrkB was in fact due to the absence of the fourth intracellular domain and not the failure in its adressing to the plasma membrane we used a kit to separate plasma membrane proteins from the whole protein extract. Following transfection of HEK293T cells with the intracellular NTSR2 mutants, whole cell lysate proteins and plasma membrane proteins were separately extracted using a specific kit (Abcam) and analyzed by immunoblot.

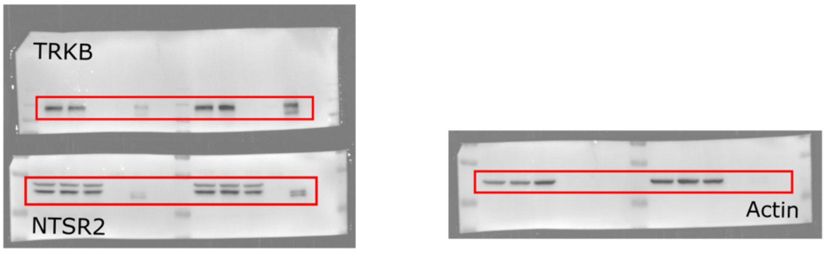

Figure 1B

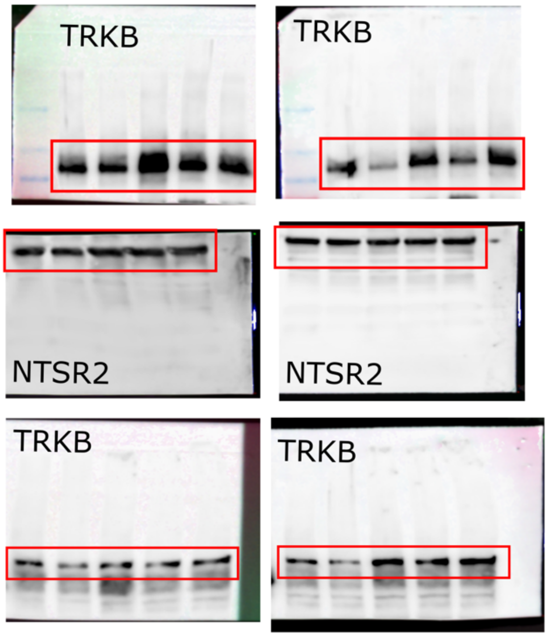

Figure 2B

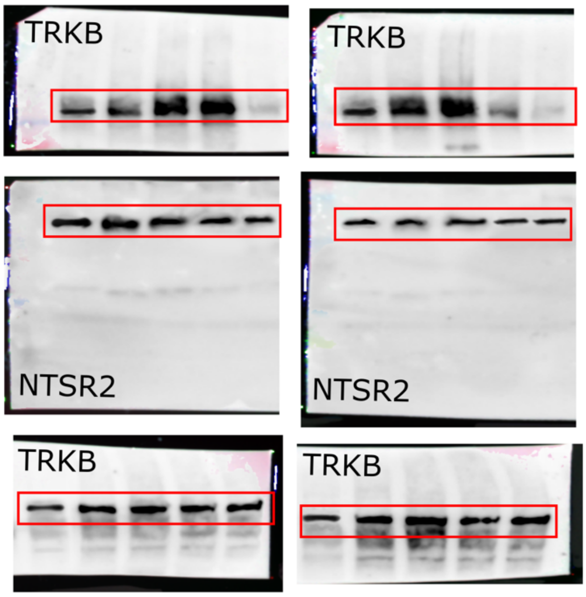

Figure 2D

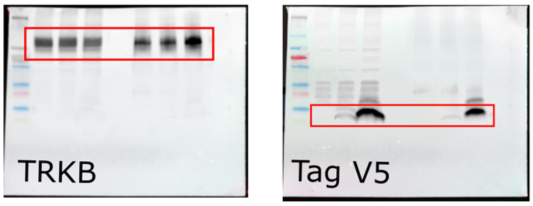

Figure 4B

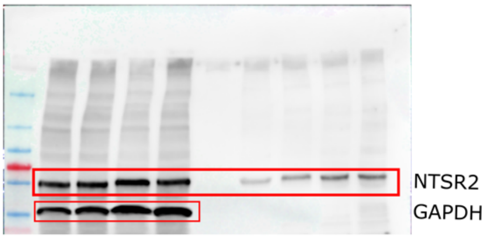

Figure S3

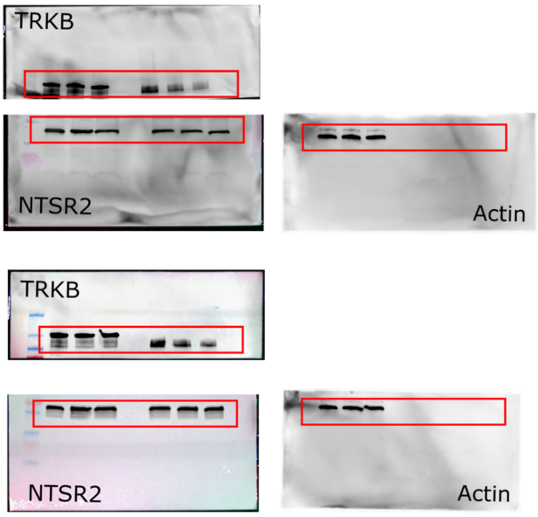

Figure 4C

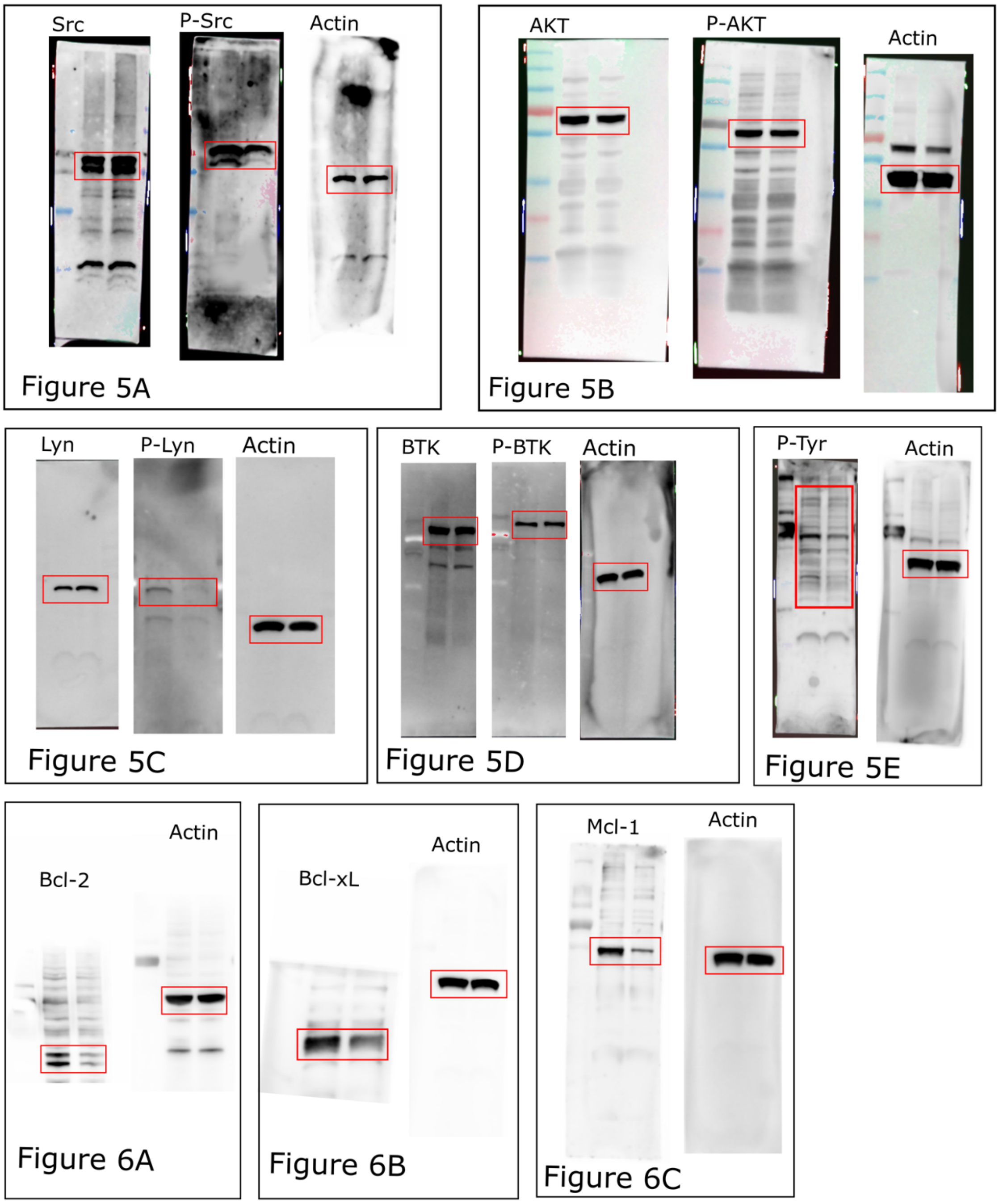

Figure S4. Whole western blot membranes. The cropped versions that are presented in the article are framed in red.
